# Unravelling Size‐Dependent and Coupled Properties in Mechanical Metamaterials: A Couple‐Stress Theory Perspective

**DOI:** 10.1002/advs.202305113

**Published:** 2024-01-02

**Authors:** Shahin Eskandari, Benyamin shahryari, Abdolhamid Akbarzadeh

**Affiliations:** ^1^ Department of Bioresource Engineering McGill University Montreal QC H9X 3V9 Canada; ^2^ Department of Mechanical Engineering McGill University Montreal QC H3A 0C3 Canada

**Keywords:** asymptotic homogenization, couple‐stress theory, mechanical metamaterial, metabeam, metaplate

## Abstract

The lack of material characteristic length scale prevents classical continuum theory (CCT) from recognizing size effect. Additionally, the even‐order material property tensors associated with CCT only characterize the materials' centrosymmetric behavior and overlook their intrinsic chirality and polarity. Moreover, CCT is not reducible to 2D and 1D space without adding couples and higher‐order deformation gradients. Despite several generalized continuum theories proposed over the past century to overcome the limitations of CCT, the broad application of these theories in the field of mechanical metamaterials has encountered significant challenges. These obstacles primarily arise from a limited understanding of the material coefficients associated with these theories, impeding their widespread adoption. Implementing a bottom‐up approach based on augmented asymptotic homogenization, a consistent and self‐sufficient effective couple‐stress theory for materials with microstructures in 3D, 2D, and 1D spaces is presented. Utilizing the developed models, material properties associated with axial‐twist, shear‐bending, bending‐twist, and double curvature bending couplings in mechanical metamaterials are characterized. The accuracy of these homogenized models is investigated by comparing them with the detailed finite element models and experiments performed on 3D‐printed samples. The proposed models provide a benchmark for the rational design, classification, and manufacturing of mechanical metamaterials with programmable coupled deformation modes.

## Graphical abstract

This paper presents a unifying, consistent, and self‐sufficient effective couple‐stress theory for metamaterials in 3D, 2D, and 1D spaces. The developed models are utilized to characterize material properties associated with axial‐twist, shear‐bending, and twist‐bending couplings in mechanical materials/metastructures. The accuracy of these models is corroborated through a comparison with detailed finite element models and experiments performed on 3D printed samples.

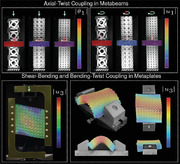

## 1 Introduction

Continuum models describe the average behavior of the material constituents under external stimuli to reduce the complexity of an extensive system composed of different components. In the first decades of the nineteenth century, the French scientists —among them Navier, Cauchy, and Poisson— developed the first linear elasticity continuum theory based on a molecular model that assumed matter as point‐particles with central interaction depending only on the body‐points' distance. However, this theory suggested that homogeneous isotropic materials were characterized by a unique elastic constant, which was not in accord with the experiments^[^ ^1^ ^]^ showing various values of Poisson's ratio for different materials. The model proposed by Green disregarded elastic bodies' molecular structure and assumed a continuous model of matter in which internal actions are derived from a quadratic potential function. His model fitted the experimental data but originated a long debate about the “actual” number of elastic constants and the “correct” model for elasticity.^[^ ^2^, ^3^ ^]^

At the beginning of the twentieth century, Voigt,^[^ ^4^ ^]^ a German physicist, resolved this controversy between the discrete and the continuous model of matter by proposing a linear elasticity theory based on Bravais's crystallographic studies. Voigt highlighted that a molecular description should not consider a single elementary mass but rather aggregations of atoms assembled and oriented in space according to the crystal's symmetries, a representative volume element in the current material design language. These bodies interact in pairs through a system of actions reducible to a force passing through the particles' center of gravity and a couple called an orienting moment that keeps the particles in their crystal layouts. Voigt did not entirely abandon the discrete description of matter suggested by the French pioneers, yet adding a couple to the central force removed unnecessary restrictions in their work. Voigt adopted the definition of internal force stress from previous works and introduced internal couple stresses. The couple‐ and force‐stresses were postulated to be dependent on the distance between the particles' center of gravity and their relative orientation, and they were employed to form a potential function. However, by hypothesizing that the particles rotate equally within a sphere of action, he removed the rotation variation (curvature) from the formulation; consequently, the mutual couples became the moment of forces, and the constitutive relations were written only for the intermolecular force‐stresses. This approach, molecular but not discarding “abstract” energetic techniques, resulted in constitutive relations for linear elasticity that confirmed Green's theory.

Neglecting the variation of interacting particles' rotation in Voigt's discrete method is equivalent to assuming quadratic variation for the strain potential function in Green's pure continuum approach. These assumptions result in the symmetric strain and force‐stress tensors being the sole deformation and stress measures in the classical continuum theory (CCT). However, these assumptions lead to several shortcomings in CCT. One major drawback is the absence of a material length scale in CCT, as it treats a solid body as infinitely divisible into infinitesimal elements with the same behavior as the bulk material. This overlooks the fact that all materials are composed of building blocks and exhibit microstructures at various length scales. While crystallography categorizes crystalline materials into thirty‐two symmetry classes, due to the central nature of stress and strain tensors, CCT can only distinguish nine classes,^[^ ^4^ ^]^ and it does not realize the chirality and polarity of crystals.^[^ ^5^ ^]^ Furthermore, the reduction of CCT as a physical model from 3D to 2D (plate theory) and 1D (beam theory) is impossible without incorporating higher‐order gradients of displacement as deformation measures.^[^ ^6^ ^]^

To date, numerous generalized continuum theories have been proposed to address the above‐mentioned CCT's shortcomings. They deviate from CCT in two ways: 1) Considering additional degrees of freedom associated with each material point (micro‐continuum theories) originated from the works of the Cosserat brothers^[^ ^6^ ^]^ and 2) contributing supplementary deformation measures into the strain energy density function (strain gradient theories). For an elastic material in the small deformation regime, the micropolar theory (MT)^[^ ^7^ ^]^ and indeterminate couple‐stress theory (ICST)^[^ ^8^, ^9^, ^10^ ^]^ have gained much attention among the micro‐continuum and strain gradient theories, respectively. However, none has proved applicable, mainly due to a notorious lack of knowledge of new material coefficients, set aside the unclear physical implication of micro‐rotations in MT^[^ ^11^ ^]^ and the indeterminacy issues in ICST.^[^ ^8^, ^9^, ^10^, ^11^, ^12^ ^]^

Metamaterials serve as artificial counterparts to natural crystalline materials. Through the deliberate design of their internal architecture and constituent material arrangements, metamaterials possess properties that surpass those of ordinary materials. Recent advancements in 3D printing, coupled with the capabilities of metamaterials, have opened up a range of applications. These include deployable materials for various purposes,^[^ ^13^ ^]^ reusable energy absorbers,^[^ ^14^ ^]^ read‐write mechanical memories,^[^ ^15^, ^16^ ^]^ and waveguiding materials,^[^ ^17^, ^18^ ^]^ among others. Since the size of the unit cell in metamaterials ranges from the sub‐micrometer scale to the centimeter scale, they are prone to the emergence of size‐effects. Certain mechanical metamaterials can be understood and characterized using classical continuum theory. These materials demonstrate phenomena such as negative Poisson's ratio,^[^ ^19^ ^]^ negative compressibility,^[^ ^20^ ^]^ negative incremental stiffness,^[^ ^14^, ^21^, ^22^ ^]^ and zero shear stiffness in *pentamode* metamaterials.^[^ ^23^ ^]^ However, there are also mechanical metamaterials that exhibit behaviors that cannot be fully explained by classical continuum theory. These include the size effect,^[^ ^24^ ^]^ compression‐twist coupling,^[^ ^25^, ^26^, ^27^, ^28^ ^]^ and acoustic activity^[^ ^29^, ^30^ ^]^ (rotation of the plane polarization of mechanical wave) in chiral metamaterials. Despite the existence of computational and experimental evidence showcasing non‐classical mechanical behavior, a widely accepted model to characterize the effective material properties associated with these behaviors has not yet been established. Obviously, CCT is not a proper model due to its shortcomings. Some authors^[^ ^25^, ^26^, ^27^, ^28^, ^31^ ^]^ have speculated that the MT is the right continuum model for this purpose, and others^[^ ^30^, ^32^, ^33^ ^]^ assumed the ICST serves the best as an effective model. A few studies^[^ ^26^, ^28^, ^30^ ^]^ just mapped the expected behavior to the predictions from selected theories and estimated the required material properties. Alternatively, some researchers have proposed mathematical methods to determine the additional effective material parameters related to the selected theory.^[^ ^31^, ^32^, ^33^ ^]^

A study leveraging a slender‐beam design and an energy‐equivalent scheme aimed to ascertain effective micropolar material properties, yet its broader application was found to be limited due to its dependence on the Euler–Bernoulli beam theory.^[^ ^31^ ^]^ In another study, a standard mechanics homogenization was implemented with additional load tests to systematically compute the effective material properties of chiral metamaterials pertinent to ICST. However, the continuity of materials in the designed unit cells as well as in the associated modeling procedure was overlooked. An exploration of the couple‐stress moduli for vertebral trabecular bone incorporated mixed boundary conditions and an equivalent strain energy method; however, its adaptability was restricted to centro‐symmetric and orthotropic microstructures.^[^ ^32^ ^]^ Echoing a review on the subject of effective generalized continuum theory for chiral metamaterials, the process of deriving effective‐medium parameters from metamaterial microstructures still poses challenges.^[^ ^27^ ^]^ Despite numerous efforts, the scientific community of rationally‐designed mechanical metamaterials remains in pursuit of a universally applicable method to find the effective generalized continuum theory for metamaterials with coupled deformation modes.

This study aims to develop a generalized continuum theory to describe the effective *quasi* *‐static* (low frequency, long wavelength) behavior of materials with microstructures at *small deformation* in the *linear elastic regime*. Assuming that the classic continuum theory governs the mechanical behavior of the base materials at the microscopic scale, we implement a bottom‐up approach based on augmented asymptotic homogenization (AAH), resulting in sets of generalized continuum theories. Presuming periodicity in all three principal directions results in a standalone consistent couple‐stress theory (CST) in 3D space, while releasing one of the periodicity conditions reduces this theory to a couple‐stress plate theory (CSPT) in 2D space, and an additional subsequent reduction leads to a couple‐stress beam theory (CSBT) in 1D space. The consistency of our couple‐stress theory stems from the deviatoric nature of the couple‐stress tensor, and it is self‐sufficient since all the effective generalized elasticity tensors are derived systematically as a by‐product of our model development.

On the foundation of our theoretical study, we found two distinct couplings in chiral mechanical metamaterials: an *axial‐twist* coupling, which was reported previously,^[^ ^25^, ^26^ ^]^ and a new *shear‐bending* coupling discovered in this study. The material properties associated with these couplings are introduced theoretically and later quantified using AAH. We examine the accuracy of the proposed effective medium theories and provide criteria for their applicability by comparing their results with a combination of experimentation and detailed analysis using the finite element method (FEM). Additionally, by implementing CSPT and the associated proposed homogenization method, the bending behavior of extruded cellular plates is studied. It is shown that a new material property (Mindlin ratio) governs whether the deformation under a one‐way bending moment is synclastic (dome‐shaped) or anticlastic (saddle‐shaped), while it was previously associated with Poisson's ratio. We also studied the bending behavior of so‐called 2D‐chiral^[^ ^34^, ^35^, ^36^ ^]^ plates and showed that these classes of materials have twist‐bending coupling in addition to the previously reported^[^ ^37^ ^]^ shear‐axial coupling. Resorting to coordinate transformation, we have derived a Mohr's circle and found two distinct sets of rotation angles where these couplings disappear and two pure bending shapes appear, one synclastic and the other anticlastic.

## 2 Mathematical Modeling

### 2.1 Augmented Asymptotic Homogenization

**Figure 1:**
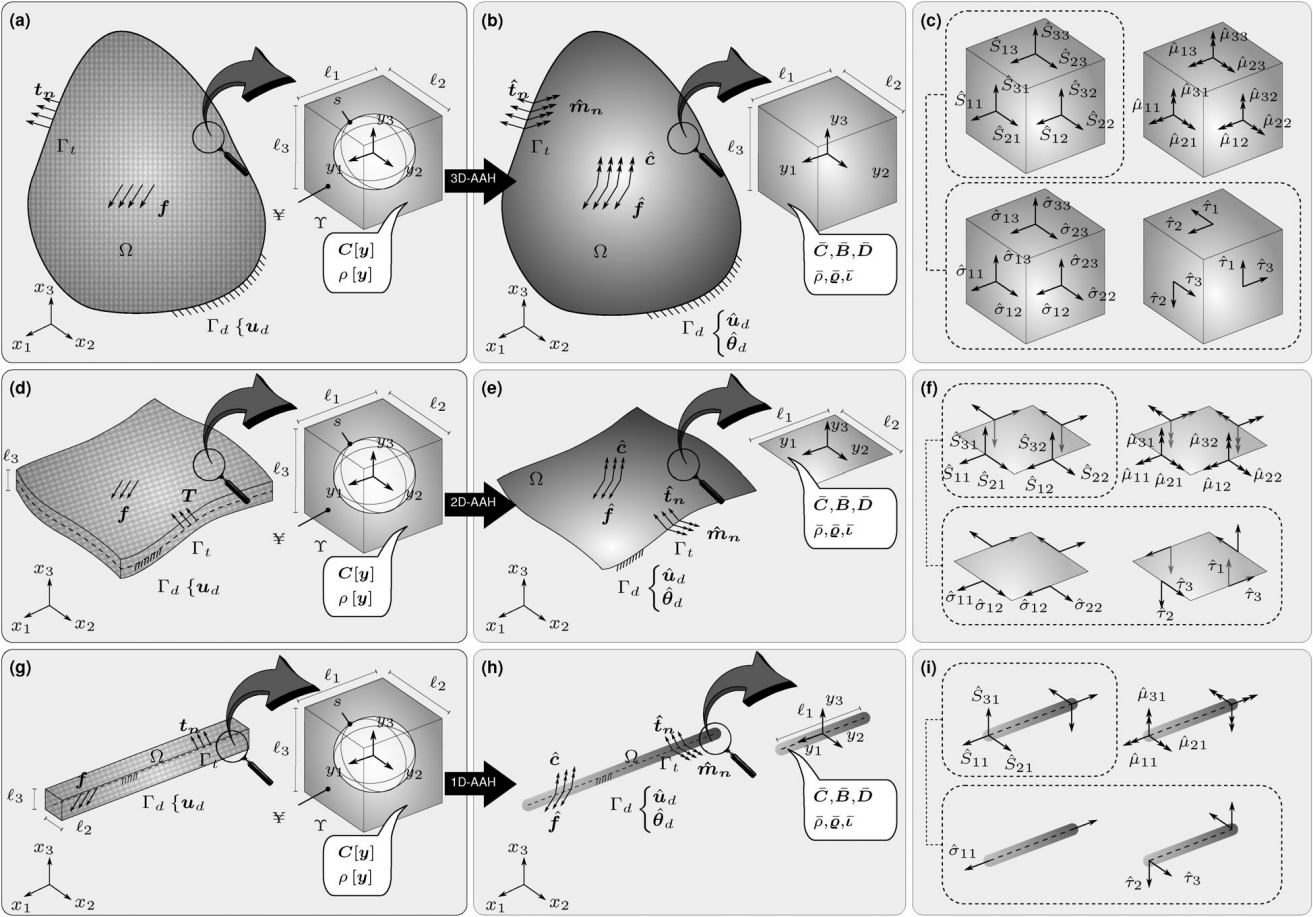
General elasticity problems in a) 3D, d) 2D, and g) 1D for a medium made of a material with a microstructure that are converted to boundary value problems in b) 3D‐CST, e) 2D‐CSPT, and h) 1D‐CSBT using AAH; the components of the stress tensor are presented in c) 3D‐CST, f) 2D‐CSPT, and i) 1D‐CSBT

Let Ω be an open subset of $R^{3}$ with a smooth boundary Γ made of a crystalline material with a periodic microstructure (**Figure** **1**a). Let the primitive unit cell Y be a parallelepiped in $R^{3}$ defined by three lattice vectors ℓ_*j*_ (*j* = 1, 2, 3), consider ¥ as the solid part of the unit cell, and take *s* as the exterior boundary of the unit cell. The problem of finding the displacement field, **u**, subjected to the body force **f**, traction **t**_*n*_ on the boundary Γ_t_ together with prescribed displacements **u**_d_ on Γ_d_ can be stated using the principle of virtual work as

(1) $$\int_{\Omega }\left(\delta u\nabla :C:u\nabla +\rho \delta u\cdot \dot{v}\right)\mathrm{dV}=\int_{\Gamma_{t}}\delta u\cdot t_{n}\mathrm{dA}+\int_{\Omega }\delta u\cdot f\mathrm{dV}$$

where, $v=\dot{u}$ is the velocity vector field; δ**u** is an arbitrary admissible virtual displacement field taking a zero value on the boundary Γ_d_; **C** and ρ are, respectively, the fourth‐order elasticity tensor with all the major (*C*_*ijkl*_ = *C*_*klij*_) and minor (*C*_*ijkl*_ = *C*_*jikl*_ = *C*_*ijlk*_) symmetries and mass density. A unique solution **u** exists for Equation (1) under the assumption that the functions **f** and **t**_*n*_ are sufficiently smooth and the boundaries Γ_t_, and Γ_d_ are regular. While it is possible to solve such a problem using finite element methods, the process of discretizing the body to represent the detailed microstructure can become a cumbersome, even with state‐of‐the‐art computational capacities. Therefore, it is desirable to develop a method that can reflect the microstructure without requiring a detailed examination of all material points in the body, particulary when focusing on the macroscopic behavior.

The partial differential equations governing the response of material with periodic microstructures include rapidly oscillating coefficients. Many researchers^[^ ^38^, ^39^ ^]^ have applied the classical asymptotic homogenization (CAH) method to map such media to a CCT and remove unnecessary details of the problem. In this approach, the displacement field is split into two components: macroscopic and microscopic, represented as $u=\bar{u}+u^{*}$. The macroscopic part $\bar{u}$ is solely governed by the overall state of the system in an average sense, while the microscopic part $u^{*}$ is approximated by its variation on multiple spatial scales due to the existence of a microstructure, including a slow variation in terms of a macroscopic coordinate **x**, and a periodic rapid variation in terms of a microscopic coordinate **y**. The asymptotic expansion of the microscopic part of displacement is $u^{*}=\eta u^{1}+\eta^{2}u^{2}+\cdots$ where **u** ^1^, **u** ^2^, etc. are periodic perturbing displacement fields produced due to the existence of the microstructure and η is the ratio of the microstructure length scale to the macroscopic length scale. Considering this expansion and using the chain rule **∇** = **∇**_**x**_ + **∇**_**y**_/η for manipulating the spatial derivatives of the double (macro and micro) scale functions, one may derive the asymptotic expansion pertinent to all other field variables. Consequently, Equation (1) is decomposed into three problems on different levels: *microscopic*, *mesoscopic*, and *macroscopic*. From the microscopic‐level problem, the variation of macroscopic fields on the microscopic level is evaluated. The mesoscopic‐level problem enables the computation of the microscopic part based on the macroscopic part of the variables. Finally, the macroscopic‐level problem produces effective constitutive and governing equations.

The classical asymptotic homogenization (CAH) and the augmented asymptotic homogenization (AAH) proposed here share the same foundational principles and procedural steps up to a certain point. The point of divergence occurs during the solution of the microscopic‐level problem. Specifically, in the CAH approach, the macroscopic displacement ($\bar{u}$) remains constant within each unit cell concerning the local coordinate (y). In contrast, the AAH method introduces a linear variation of $\bar{u}$ with respect to the local coordinates of each unit cell (y); this variation corresponds to the axial rotation vector. Subsequent steps in both methods remain identical; however, the distinction manifested above, which incorporates an axial rotation vector into the kinematics of the effective medium, leads to two entirely distinct outcomes. The CAH method results in effective Cauchy elasticity (aka CCT), whereas AAH yields effective couple‐stress elasticity (aka CST).

#### 2.1.1 Microscopic Level

Careful examination of the classical AH reveals that only a trivial solution $\bar{u}\nabla_{y}=0$ is considered for the microscopic‐level problem

(2) $$\int_{¥}\left(\delta u\nabla_{y}\right)\;:\;C\;:\;\left(\bar{u}\nabla_{y}\right)\mathrm{dV}=0$$

resulting in constant macroscopic fields over the representative volume element (RVE). The assumption that leads to this solution is the periodicity of macroscopic displacement field $\bar{u}$ with respect to **y** (see page 253 of ref. [40]) which is in contradiction with the existence of any macroscopic deformation. A simple contrary instance is the unidirectional tension of a material in which the opposite boundaries of the RVE move away from each other and the displacement field is obviously not periodic. Removing such an unphysical assumption and using the symmetry of the elasticity tensor **C**, the complete solution of the microscopic problem is found as

(3) $$\bar{u}\nabla_{y}=\hat{\theta }\times I\Rightarrow \bar{u}\left[x,y\right]=\hat{u}\left[x\right]+\hat{\theta }\left[x\right]\times y$$

where $\hat{u}$ and $\hat{\theta }$ are the translation vector and the rotation pseudo‐vector^[^ ^41^ ^]^ describing the macroscopic kinematics of an RVE whose local coordinate origin is situated at coordinate **x**. Applying Whitaker's averaging theorem^[^ ^42^ ^]^ for continuous interfaces, 〈**∇**_**y**_ × **u**〉_Y_ = **∇**_**x**_ × 〈**u**〉_Y_, and using Equation (3), one can write

(4) $$\begin{array}{cc} \hat{u}\left(x\right) & ={\left\langle u\left(x,y\right)\right\rangle }_{Y}\\ \hat{\theta }\left(x\right) & =1/2\nabla_{x}\times \hat{u}\left(x\right) \end{array}$$

provided that the local coordinate system is placed at the geometrical center of each RVE, 〈**y**〉_Y_ = 0, where ${\left\langle □\right\rangle }_{Y}=\frac{1}{\left|Y\right|}\int_{¥}\left(□\right)\mathrm{dV}$ represents the volumetric average over the unit cell. It is worth mentioning that we have used the fact that the volumetric average of the fluctuating part of displacement is zero, ${\left\langle u^{*}\right\rangle }_{Y}=0$. Equation (4) proves that $\hat{\theta }$ is the macroscopic rotation that the unit cell experiences due to the displacement field $\hat{u}$, not an independent micro‐rotation similar to the one introduced in micropolar theory.^[^ ^7^ ^]^

#### 2.1.2 Mesoscopic Level

Inserting the macroscopic displacement into the mesoscopic‐level problem results in the following cell‐problem

(5) $$\int_{¥}\left(\delta u\nabla_{y}\right):C:\left(\bar{\varepsilon }\;+\;\varepsilon^{*}\right)\mathrm{dV}=0$$

where $\bar{\varepsilon }=\hat{\varepsilon }+S:\left({\hat{\kappa }}^{T}\times y\right)$ and $\varepsilon^{*}=S:u^{1}\nabla_{y}$ are the macroscopic and microscopic parts of strain field; $\hat{\varepsilon }=S:\hat{u}\nabla_{x}$ and $\hat{\kappa }=\hat{\theta }\nabla_{x}$ are the second order symmetric strain tensor and deviatoric curvature pseudo‐tensor;^[^ ^41^ ^]^ S is the fourth‐order symmetric identity tensor. Solution to the cell‐problem Equation (5) results in the derivation of all the microscopic fields in terms of the macroscopic variables (see Section S1.3, Supporting Information).

#### 2.1.3 Macroscopic Level

Next, using the solutions derived from the cell‐problem, macroscopic‐level problem leads to the principle of virtual work associated with the effective couple‐stress theory as follows

(6) $$\begin{array}{cc} & \int_{\Omega }\left(\delta \hat{\varepsilon }:\hat{\sigma }+\delta \hat{\kappa }:\hat{\mu }\right)\;\mathrm{dV}+\int_{\Omega }\left(\delta \hat{u}\cdot \dot{\hat{L}}+\delta \hat{\theta }\cdot \dot{\hat{J}}\right)\;\mathrm{dV}\\ & \;=\int_{\Omega }\left(\delta \hat{u}\cdot \hat{f}+\delta \hat{\theta }\cdot \hat{c}\right)\;\mathrm{dV}+\int_{\Gamma_{t}}\left(\delta \hat{u}\cdot {\hat{t}}_{n}+\delta \hat{\theta }\cdot \hat{M}\right)\;\mathrm{dA} \end{array}$$

where the first integral in the left‐hand side is pertinent to the virtual elastic potential energy δ*U*, the second one represents the virtual kinetic energy δ*K*, and the right‐hand side is the virtual external work δ*W*_ext_ associated with the external generalized body forces and tractions. The work conjugates of symmetric strain tensor $\hat{\varepsilon }$ and the deviatoric curvature pseudo‐tensor $\hat{\kappa }$ as the generalized measures of deformation are symmetric part of force‐stress tensor $\hat{\sigma }$ and deviatoric couple‐stress pseudo‐tensor $\hat{\mu }$, respectively, as defined below

(7) $$\hat{\sigma }={\left\langle C:\left(\bar{\varepsilon }+\varepsilon^{*}\right)\right\rangle }_{Y},\;\hat{\mu }={\left\langle y\times C:\left(\bar{\varepsilon }+\varepsilon^{*}\right)\right\rangle }_{Y}$$

The deviatoric state of curvature and couple‐stress originates from the divergence‐free nature of the rotation axial vector, resulting in ${\hat{\kappa }}_{kk}=0$ and the definition of couple‐stress as the derivative of the energy density function *U* with respect to curvature ${\hat{\mu }}_{kk}=\partial U/\partial {\hat{\kappa }}_{kk}=0$. Generalization also takes place in the kinetic energy terms, where $\hat{L}$ and $\hat{J}$ represent the effective linear and angular momentum and are calculated as

(8) $$\hat{L}={\langle \rho \bar{v}\rangle }_{Y},\;\hat{J}={\langle \rho y\times \bar{v}\rangle }_{Y}$$

where $\bar{v}=\hat{v}+y\times \hat{\omega }$ is the total macroscopic velocity and $\hat{v}=\dot{\hat{u}}$ and $\hat{\omega }=\dot{\hat{\theta }}$ are the translational and angular velocities. Moreover, $\hat{f}$ and $\hat{c}$ as the effective body‐force and ‐couple, along with $\hat{T}$ and $\hat{M}$ as the effective force‐ and couple‐traction, and ${\hat{u}}_{d}$ and ${\hat{\theta }}_{d}$ as the predefined displacement and rotation, specify the applied loads and boundary conditions (see Section S1.4, Supporting Information, for more details). A schematic representation of this model is depicted in Figure 1b.

### 2.2 Couple Stress Theory

#### 2.2.1 Governing Equations

Implementing the divergence theorem on Equation (6), the governing equations in the context of couple‐stress theory are derived as

(9) $$\hat{\sigma }\cdot \nabla_{x}+\hat{f}-\dot{\hat{L}}+\frac{1}{2}\nabla_{x}\times \left(\hat{\mu }\cdot \nabla_{x}+\hat{c}-\dot{\hat{J}}\right)=0$$

The laws of conservation of linear and angular momentum in the context of couple‐stress theory are also derived as

(10) $$\hat{S}\cdot \nabla_{x}+\hat{f}=\dot{\hat{L}},\;\hat{\mu }\cdot \nabla_{x}+\hat{c}-\varepsilon :\hat{S}=\dot{\hat{J}}$$

The asymmetric stress tensor can be decomposed into symmetric and antisymmetric parts $\hat{S}=\hat{\sigma }-\varepsilon .\hat{\tau }$, where **ε** is the permutation tensor. Moreover, $\hat{\tau }=-1/2\;\varepsilon :\hat{S}$ is the stress axial‐vector and can be obtained from the conservation of angular momentum as $\hat{\tau }=-1/2\left(\hat{\mu }\cdot \nabla_{x}+\hat{c}-\dot{\hat{J}}\right)$. Inserting that into the conservation of linear momentum reproduces Equation (9). The components of force‐ and couple‐stress tensors are depicted in Figure 1c. It is important to note that only the symmetric part of stress $\hat{\sigma }$ contributes to the energy density function and therefore is determined through constitutive equations, while its antisymmetric part $\hat{\tau }$ is determined from the angular momentum conservation equation. This is similar to the well‐known Love‐Kirchhoff plate theory and Bernoulli–Euler beam theory, where the out of plane shear forces are determined indirectly using the moment equilibrium. Moreover, since the couple‐stress pseudo‐tensor is deviatoric by nature, its spherical part is zero. Therefore, the resulting couple‐stress theory does not suffer from the indeterminacy issue in classical couple‐stress theory.

The Dirichlet and Neumann boundary conditions related to couple‐stress theory can be found in Section S1.5, Supporting Information, and are summarized as follows:

(11a) $$\hat{u}={\hat{u}}_{d}\;\forall x\in \Gamma_{d}$$

(11b) $$\hat{\theta }={\hat{\theta }}_{d}\;\forall y\in \Gamma_{d}$$

(11c) $$n\cdot \hat{S}={\hat{t}}_{n}\;\forall x\in \Gamma_{t}$$

(11d) $$n\cdot \hat{\mu }={\hat{m}}_{n}\;\forall x\in \Gamma_{t}$$

where the defined displacement, rotation, force‐traction, and couple‐traction on the boundary are outlined as follows:

(12a) $$\begin{array}{cc} & {\hat{u}}_{d}={\langle u_{d}\rangle }_{\Gamma_{Y}} \end{array}$$

(12b) $${\hat{\theta }}_{d}=\frac{1}{2}{\left\langle \nabla_{y}\times u_{d}\right\rangle }_{\Gamma_{Y}}$$

(12c) $${\hat{t}}_{n}={\langle t_{n}\rangle }_{\Gamma_{Y}}$$

(12d) m^n=y×tnΓY12

where Γ_Y_ is the boundary of the RVE which belongs to the surface Γ_t_ and |Γ_Y_| is its area. Also, ${\left\langle □\right\rangle }_{\Gamma_{Y}}=\frac{1}{|\Gamma_{Y}|}\int_{\Gamma_{Y}}\left(□\right)d\Gamma_{Y}$ represents the areal average over the boundary of the RVE.

As demonstrated in Equation (12), all boundary conditions pertaining to the effective model are directly derived from the boundary conditions applied to the real model. The rotation boundary condition is obtained from the slope of the actual applied displacement, while the couple‐traction condition arises from the moment of the applied real traction along the boundary.

#### 2.2.2 Constitutive Relations

The constitutive equations are derived from Equations (7) and (8) as

(13a) $$\hat{\sigma }=\bar{C}:\hat{\varepsilon }+\bar{B}:\hat{\kappa }$$

(13b) $$\hat{\mu }={\bar{B}}^{T}:\hat{\varepsilon }+\bar{D}:\hat{\kappa }$$

(13c) $$\hat{L}=\bar{\rho }\;\hat{v}+\bar{\varrho }\times \hat{\omega }$$

(13d) $$\hat{J}=\hat{v}\times \bar{\varrho }+\bar{\iota }\cdot \hat{\omega }$$

where $\bar{C}$ is the classical fourth‐order effective elasticity tensor; $\bar{B}$ is the fourth‐order effective elastic coupling axial‐tensors; $\bar{D}$ is the fourth‐order elastic bending tensor; $\bar{\rho }$ is the relative density, and $\bar{\varrho }$ and $\bar{\iota }$ are the axial‐vector and the second‐order tensor corresponding to the effective first and the second moment of inertia per unit area, respectively.

Upon a uniform scaling of the unit cell by a factor of ℓ, in contrast to the classic elasticity tensor $\bar{C}$ and the relative density $\bar{\rho }$ which are scale invariance (x¬∝y), the elastic coupling pseudo‐tensors $\bar{A}$ and $\bar{B}$, and the first moment of inertia axial‐vector $\bar{\varrho }$ are first‐order scale‐dependent (∝ℓ), while the elastic bending tensor $\bar{D}$ and the second moment of inertia tensor $\bar{\iota }$ are second‐order scale‐dependent (∝ℓ^2^).

#### 2.2.3 Physical Symmetries

Considering the symmetries of $\hat{\sigma }$ and $\hat{\varepsilon }$, deviatoric nature of $\hat{\mu }$ and $\hat{\kappa }$ and the fact that for an elastic material, the elastic strain energy function must be continuously differentiable, this results in the symmetry conditions in material property matrices (see Section S3.1, Supporting Information, for more details). Taking these symmetry conditions into account, at one extreme, the number of independent elastic components for the most generalized case of anisotropy reaches 105 (21 parameters for $\bar{C}$, 48 parameters for $\bar{B}$, and 36 parameters for $\bar{D}$), while on the other end, for isocentral material, the minimum number of independent material parameters becomes four (two parameters for $\bar{C}$, zero parameters for $\bar{B}$, and two parameters for $\bar{D}$). Refer to Section S5.1, Supporting Information, for additional details.

In crystal physics, Neumann's principle asserts that the physical properties of a crystal, when subjected to certain symmetry operations, must remain invariant if the crystal itself possesses those symmetries.^[^ ^43^ ^]^ Interestingly, some physical properties might exhibit greater symmetry than those of the crystal. As a result, not every physical property is effective in elucidating the true point group symmetries inherent to crystals. A notable example is the Cauchy elasticity tensor ($\bar{C}$), which is a centrosymmetric physical property. The imposed minor symmetries on this tensor reduce the classical elasticity tensor categories to nine, as described by Voigt.^[^ ^4^ ^]^ However, within the context of couple stress theory, other physical properties emerge that can distinguish among all symmetry classes. For instance, the elastic bending tensor ($\bar{D}$) possesses major symmetry, characterizing it as a centrosymmetric physical property. This tensor enables the classification of crystals into the eleven distinct Laue classes.^[^ ^44^ ^]^ In contrast, the elastic coupling axial tensor ($\bar{B}$) lacks major symmetry, allowing it to distinguish non‐centrosymmetry, polarity, chirality, and thus all thirty‐two symmetry classes. Furthermore, the first moment of inertia ($\bar{\varrho }$), being a first‐order axial tensor (vector), can identify polarization but remains ineffective at distinguishing the chirality of crystals.

#### 2.2.4 Crystallographic Symmetries

A primitive unit cell is the smallest volume of a crystalline metamaterial, which builds up the whole crystal structure by tessellation along three spatial directions. Parallelepiped is the only regular geometrical body that can fill the space completely without any gap by tessellation; therefore, every imaginable crystal belongs to one of the seven crystal systems that are characterized by three lattice constants (ℓ_1_, ℓ_2_, ℓ_3_) and three angles between them (α_1_, α_2_, α_3_) (see Figure S8a, Supporting Information). Crystalline metamaterials may be additionally categorized into thirty‐two classes based on the intrinsic symmetries of their unit cells. The thirty‐two classes, their symmetries, and the number of independent material parameters required to model them are given in Table S1, Supporting Information, and the graphical representation of these classes, as well as their point, axis, and planes of symmetry, are depicted in Figure S8b, Supporting Information. Based on Neumann's Principle^[^ ^43^ ^]^ if a crystal possesses certain symmetry operations, all of its physical properties must be invariant with regard to the same symmetry operations. By implementing this principle, the form of the total generalized elasticity and density matrices associated with each crystal class can be determined.

#### 2.2.5 Materials Belonging to Chiral Cubic Crystal No.29 (432)

Due to the periodic nature of crystalline metamaterials, they are not able to show isotropic behavior in general in a 3D space. The maximum symmetry possible in such materials belongs to the crystal classes No.28 ($m\bar{3}m$) and No.29 (432). These two crystal classes have the same rotational symmetries, yet the existence of a center of inversion makes crystal class No.28 *centrosymmetric*, while its absence makes crystal class No.29 *chiral*. In these subsections, we provide the constitutive laws, compliance relations, and positive definiteness conditions for these two crystal classes.

One may decompose any 3D second‐order array $\Lambda_{ij}$ (*i* = 1, 2, 3) into spherical (volumetric), symmetric‐deviatoric, and antisymmetric parts as $\Lambda_{ij}=\Lambda_{v}\delta_{ij}+\Lambda_{\left(ij\right)}+\Lambda_{\left[ij\right]}$, where $\Lambda_{v}=1/3\Lambda_{kk}$, $\Lambda_{\left[ij\right]}=1/2\left(\Lambda_{ij}-\Lambda_{ji}\right)$ and $\Lambda_{\left(ij\right)}=1/2\left(\Lambda_{ij}+\Lambda_{ji}\right)-\Lambda_{v}\delta_{ij}$ are antisymmetric and symmetric‐deviatoric parts, respectively. The symmetric‐deviatoric part may additionally break into diagonal and off‐diagonal parts. This decomposition is useful, as it is similar to the classical continuum theory where independent material parameters characterize the axial and shear modes of deformation in materials with cubic symmetry. Similarly, in couple‐stress theory, the torsional and bending modes are also characterized independently. Due to the symmetric nature of $\hat{\sigma }$ and $\hat{\varepsilon }$, and deviatoric nature of $\hat{\mu }$ and $\hat{\kappa }$, we have ${\hat{\sigma }}_{v}=3K{\hat{\varepsilon }}_{v}$ and ${\hat{\mu }}_{\left[ij\right]}=2\gamma \;{\hat{\kappa }}_{\left[ij\right]}$, and the axial‐tensor $\bar{B}$ only couples the symmetric‐deviatoric parts of the force and deformation measures as

(14) $$\begin{array}{cccc} & \text{diagonal}i=j & & \text{off-diagonal}i\neq j\\ & |\begin{array}{cc} & {\hat{\sigma }}_{\left(ij\right)}=2G_{a}{\hat{\varepsilon }}_{\left(ij\right)}+2\beta_{a-t}{\hat{\kappa }}_{\left(ij\right)}\\ & {\hat{\mu }}_{\left(ij\right)}=2\beta_{a-t}{\hat{\varepsilon }}_{\left(ij\right)}+2\eta_{t}{\hat{\kappa }}_{\left(ij\right)} \end{array}, & & |\begin{array}{cc} & {\hat{\sigma }}_{\left(ij\right)}=2G_{s}{\hat{\varepsilon }}_{\left(ij\right)}+2\beta_{s-b}{\hat{\kappa }}_{\left(ij\right)}\\ & {\hat{\mu }}_{\left(ij\right)}=2\beta_{s-b}{\hat{\varepsilon }}_{\left(ij\right)}+2\eta_{b}{\hat{\kappa }}_{\left(ij\right)} \end{array} \end{array}$$

where, *K*, *G*_a_, and *G*_s_ are the classical bulk, axial, and shear moduli; β_a‐t_ and β_s‐b_ are torsional and bending coupling moduli; η_t_, η_b_, and γ are torsional, anticlastic (symmetric) bending, and synclastic (antisymmetric) bending moduli, respectively. The linear and angular momentum may also be determined as $\hat{L}=\bar{\rho }\;\hat{v}$ and $\hat{J}=\bar{\iota }\;\hat{\omega }$, where $\bar{\iota }$ is the isotropic second moment of inertia.

In what follows, by inverting the constitutive relations, we determine the compliance relations in terms of the local field variables. The spherical part of strain ${\hat{\varepsilon }}_{v}={\hat{\sigma }}_{v}/3K$ and the antisymmetric part of curvature ${\hat{\kappa }}_{\left[ij\right]}={\hat{\mu }}_{\left[ij\right]}/2\gamma$ are decoupled from the symmetric‐deviatoric components provided below

(15) $$\begin{array}{cccc} & \text{diagonal}\;i=j & & \text{off-diagonal}\;i\neq j\\ & |\begin{array}{cc} & {\hat{\varepsilon }}_{\left(ij\right)}=\frac{{\hat{\sigma }}_{\left(ij\right)}}{2G_{a}^{\prime }}+\frac{{\hat{\mu }}_{\left(ij\right)}}{2\beta_{a-t}^{\prime }}\\ & {\hat{\kappa }}_{\left(ij\right)}=\frac{{\hat{\sigma }}_{\left(ij\right)}}{2\beta_{a-t}^{\prime }}+\frac{{\hat{\mu }}_{\left(ij\right)}}{2\eta_{t}^{\prime }} \end{array}, & & |\begin{array}{cc} & {\hat{\varepsilon }}_{\left(ij\right)}=\frac{{\hat{\sigma }}_{\left(ij\right)}}{2G_{s}^{\prime }}+\frac{{\hat{\mu }}_{\left(ij\right)}}{2\beta_{s-b}^{\prime }}\\ & {\hat{\kappa }}_{\left(ij\right)}=\frac{{\hat{\sigma }}_{\left(ij\right)}}{2\beta_{s-b}^{\prime }}+\frac{{\hat{\mu }}_{\left(ij\right)}}{2\eta_{b}^{\prime }} \end{array} \end{array}$$

where the primed material parameters are modified due to the chirality as follows

(16) $$\begin{array}{cc} G_{a}^{\prime }=G_{a}\left(1-\alpha_{a-t}\right), & G_{s}^{\prime }=G_{s}\left(1-\alpha_{s-b}\right),\\ \eta_{t}^{\prime }=\eta_{t}\left(1-\alpha_{a-t}\right), & \eta_{b}^{\prime }=\eta_{b}\left(1-\alpha_{s-b}\right),\\ \beta_{a-t}^{\prime }=\beta_{a-t}\left(1-\alpha_{a-t}^{-1}\right), & \beta_{s-b}^{\prime }=\beta_{s-b}\left(1-\alpha_{s-b}^{-1}\right) \end{array}$$

where $\alpha_{a-t}=\left({\beta_{a-t}}^{2}\right)/\left(G_{a}\eta_{t}\right)$ and $\alpha_{s-b}=\left({\beta_{s-b}}^{2}\right)/\left(G_{s}\;\eta_{b}\right)$ are the measures of torsional and bending chirality of materials, and we name them as Lake's chirality ratios in the honor of *R. S. Lakes*, who was the first to study the effect of non‐centrosymmetry in the context of generalized continuum mechanics^[^ ^45^ ^]^ and realized the first 3D chiral mechanical metamaterial.^[^ ^25^ ^]^ The following relations exist among the compliance and constitutive parameters

(17) $$\begin{array}{c} \begin{array}{cccccccccccc} E & = & \frac{9G_{a}^{\prime }K}{3K+G_{a}^{\prime }}, & \nu & = & \frac{3K-2G_{a}^{\prime }}{2(3K+G_{a}^{\prime })}, & K & = & \frac{E}{3(1-2\nu )}, & G_{a}^{\prime } & = & \frac{E}{2(1+\nu )},\\ I & = & \frac{4\eta_{b}^{\prime }\gamma }{\eta_{b}^{\prime }+\gamma }, & \varsigma & = & \frac{\gamma -\eta_{b}^{\prime }}{\eta_{b}^{\prime }+\gamma }, & \gamma & = & \frac{I}{2(1-\varsigma )}, & \eta_{b}^{\prime } & = & \frac{I}{2(1+\varsigma )} \end{array} \end{array}$$

where *E* and ν are the Young's modulus and Poisson's ratio, while we name *I* and *ς* Cosserat bending modulus and Mindlin's bending ratio after Cosserat brothers and *R*. *D. Mindlin* for their significant contributions to generalized continuum mechanics.

By setting the eigenvalues of the total generalized elasticity matrix greater than zero, the positive definiteness conditions for chiral cubic crystal No.29 are determined as *G*_a_ > 0, *G*_s_ > 0, *K* > 0, η_t_ > 0, η_b_ > 0, γ > 0, $0\leq \alpha_{a-t}<1$, and $0\leq \alpha_{s-b}<1$ in terms of stiffness material parameters and as *E* > 0, −1 < ν < 0.5, *I*> 0, and −1 < ς < 1 in terms of compliance material parameters.

### 2.3 Couple Stress Plate Theory

The couple stress plate theory is constructed on the basis of the CST by assuming zero macroscopic variation for the field variables in the direction of the thickness (here we consider ∂/∂*x*_3_ = 0)—that is, the macroscopic displacement field varies only with *x*_1_, *x*_2_. Additionally, like any other plate theories, the top and bottom planes that are perpendicular to *x*_3_ are free surfaces—that is, no force‐ and couple‐stress components act on them (${\hat{S}}_{i3}=0$ and ${\hat{\mu }}_{i3}=0$ for *i* = 1⋅⋅⋅3). In this theory, the material is made of the periodic tessellation of unit cells in only two planar directions. Consequently, the microscopic displacement field u* varies in all three microscopic directions but is only periodic in *y*_1_ and *y*_2_. Hence, the periodic boundary conditions used in homogenization would only exist in these two planar directions. A representative configuration of the 2D‐AAH is depicted in Figure 1d. Considering the above‐mentioned variations for field variables, some of the deformation measures (${\hat{\varepsilon }}_{33}$, ${\hat{\kappa }}_{13}$, ${\hat{\kappa }}_{23}$, and ${\hat{\kappa }}_{33}$) do not contribute to the energy density function, and, hence, the stress measures associated with them (${\hat{\sigma }}_{33}$, ${\hat{\mu }}_{13}$, ${\hat{\mu }}_{23}$, and ${\hat{\mu }}_{33}$) become zero. Additionally, in the homogenization procedure, because of the absence of the periodic condition in the *y*_3_ direction, the macroscopic displacement gradients $\partial {\hat{u}}_{3}/\partial x_{1}$ and $\partial {\hat{u}}_{3}/\partial x_{2}$ do not produce any deformation and only induce rigid rotation in the unit cell, as demonstrated in Figure S6, Supporting Information. Consequently, the shear strains ${\hat{\varepsilon }}_{13}$ and ${\hat{\varepsilon }}_{23}$ are not included in the energy density function, and therefore force‐stresses ${\hat{\sigma }}_{13}$ and ${\hat{\sigma }}_{23}$ are zero, that is, ${\hat{\tau }}_{1}={\hat{S}}_{32}$ and ${\hat{\tau }}_{2}=-{\hat{S}}_{31}$. Accordingly, one can also write ${\hat{\theta }}_{1}={\hat{u}}_{3,2}$, and ${\hat{\theta }}_{2}=-{\hat{u}}_{3,1}$ leading to ${\hat{\kappa }}_{22}=-{\hat{\kappa }}_{11}$, and consequently ${\hat{\mu }}_{22}=-{\hat{\mu }}_{11}$ which is in accordance with the deviatoric nature of curvature and couple‐stress. The components of all the vectors and tensors associated with the CSPT are compared to those of the CST in Table S2, Supporting Information.

#### 2.3.1 Governing Equations

A schematic representation of the boundary value problem in the context of CSPT is depicted in Figure 1e, along with the components of force‐ and couple‐stresses in Figure 1f. Considering the nonzero components of force‐ and couple‐stress and replacing **∇_x_** = (∂/∂*x*_1_, ∂/∂*x*_2_, 0) in Equation (10), one can write the linear and angular conservation laws in the context of CSPT in 2D space. The conservation laws and the governing equations associated with the CSPT are compared to those of the CST in Tables S3 and S4, Supporting Information, respectively. By eliminating the in‐plane couple‐stress components (${\hat{\mu }}_{31}$ and ${\hat{\mu }}_{32}$), which implies the assumption of symmetric in‐plane force‐stresses (${\hat{S}}_{12}={\hat{S}}_{21}$), the governing equations of the CSPT can be simplified and transformed into the governing equations of Love–Kirchhoff plate theory.

#### 2.3.2 Constitutive Equations

Taking into account the reduced number of macroscopic deformation measures, one can derive all the constitutive relations associated with CSPT by using AAH (see Section S2, Supporting Information). Accordingly, the generalized elasticity tensor fro CSPT in Voigt notation becomes

(18) $$\left[\begin{array}{c} {\hat{\sigma }}_{11}\\ {\hat{\sigma }}_{22}\\ {\hat{\sigma }}_{12}\\ {\hat{\mu }}_{11}\\ {\hat{\mu }}_{22}\\ {\hat{\mu }}_{12}\\ {\hat{\mu }}_{21}\\ {\hat{\mu }}_{31}\\ {\hat{\mu }}_{32} \end{array}\right]=\left[\begin{array}{ccccccccc} {\bar{C}}_{11} & {\bar{C}}_{12} & {\bar{C}}_{14} & {\bar{B}}_{11} & {\bar{B}}_{12} & {\bar{B}}_{14} & {\bar{B}}_{15} & {\bar{B}}_{17} & {\bar{B}}_{19}\\ & {\bar{C}}_{22} & {\bar{C}}_{24} & {\bar{B}}_{21} & {\bar{B}}_{22} & {\bar{B}}_{24} & {\bar{B}}_{25} & {\bar{B}}_{27} & {\bar{B}}_{29}\\ & & {\bar{C}}_{44} & {\bar{B}}_{41} & {\bar{B}}_{42} & {\bar{B}}_{44} & {\bar{B}}_{45} & {\bar{B}}_{47} & {\bar{B}}_{49}\\ & & & {\bar{D}}_{11} & {\bar{D}}_{12} & {\bar{D}}_{14} & {\bar{D}}_{15} & {\bar{D}}_{17} & {\bar{D}}_{19}\\ & & & & {\bar{D}}_{22} & {\bar{D}}_{24} & {\bar{D}}_{25} & {\bar{D}}_{27} & {\bar{D}}_{29}\\ & & & & & {\bar{D}}_{44} & {\bar{D}}_{45} & {\bar{D}}_{47} & {\bar{D}}_{49}\\ & & & & & & {\bar{D}}_{55} & {\bar{D}}_{57} & {\bar{D}}_{59}\\ & & & & & & & {\bar{D}}_{77} & {\bar{D}}_{79}\\ & & & & & & & & {\bar{D}}_{99} \end{array}\right]\cdot \left[\begin{array}{c} {\hat{\varepsilon }}_{11}\\ {\hat{\varepsilon }}_{22}\\ 2{\hat{\varepsilon }}_{12}\\ {\hat{\kappa }}_{11}\\ {\hat{\kappa }}_{22}\\ {\hat{\kappa }}_{12}\\ {\hat{\kappa }}_{21}\\ {\hat{\kappa }}_{31}\\ {\hat{\kappa }}_{32} \end{array}\right]$$

Similarly, by disregarding the in‐plane couple‐stress components (${\hat{\mu }}_{31}$ and ${\hat{\mu }}_{32}$) and curvature components (${\hat{\kappa }}_{31}$ and ${\hat{\kappa }}_{32}$), the constitutive relations of CSPT can be simplified to match those of Love–Kirchhoff plate theory. It is worth noting that asymptotic homogenization for the Love–Kirchhoff plate theory has already been established in previous works.^[^ ^46^, ^47^ ^]^ By using the same argument presented in Section 2.2.3, the maximum number of independent elastic components for the most general case of anisotropy for CSPT becomes 36 (6 parameters for **C**, 15 parameters for **B**, and 15 parameters for **D**), while on the other end, for isocentral material, the minimum number of independent material parameters becomes five (two parameters for **C**, zero parameters for **B**, and three parameters for **D**). Refer to Section S5.2, Supporting Information, for additional details.

#### 2.3.3 Plates Belonging to Chiral Tetragonal Classes No.15 (422)

The microstructured plates can not have cubic symmetry since they are periodic only in two directions. Here we consider the tetragonal symmetry class No.15. Similar to a 3D second‐order array, one may decompose a 2D second‐order array $\Lambda_{ij}$ (*i* = 1, 2) into circular (areal), symmetric‐deviatoric and antisymmetric parts as $\Lambda_{ij}=\Lambda_{a}\delta_{ij}+\Lambda_{\left(ij\right)}+\Lambda_{\left[ij\right]}$, where $\Lambda_{a}=1/2\Lambda_{kk}$ is the circular part. Due to the symmetric nature of $\hat{\sigma }$ and $\hat{\varepsilon }$, and the deviatoric nature of $\hat{\mu }$ and $\hat{\kappa }$, similar to the CST in 3D space, we have decoupled circular stress (${\hat{\sigma }}_{a}=2K{\hat{\varepsilon }}_{a}$) and the antisymmetric couple‐stress (${\hat{\mu }}_{\left[ij\right]}=2\gamma \;{\hat{\kappa }}_{\left[ij\right]}$), and the axial‐tensor $\bar{B}$ only couples the symmetric‐deviatoric parts as presented in Equation (14). The circular part of strain ${\hat{\varepsilon }}_{a}={\hat{\sigma }}_{a}/2K$ and antisymmetric part of curvature ${\hat{\kappa }}_{\left[ij\right]}={\hat{\mu }}_{\left[ij\right]}/2\gamma$ are decoupled from the symmetric‐deviatoric components for which Equation (15) holds. The primed material parameters are modified due to the chirality, according to Equation (16). The relations among the compliance and constitutive parameters associated to elastic bending tensor $\bar{D}$ are the same as the ones defined for CST Equation (16), yet they change for elasticity tensor $\bar{C}$ to the following

(19) $$\begin{array}{c} E=\frac{4G_{a}^{\prime }K}{K+G_{a}^{\prime }},\;\nu =\frac{K-G_{a}^{\prime }}{K+G_{a}^{\prime }},\;K=\frac{E}{2(1-\nu )},\;G_{a}^{\prime }=\frac{E}{2(1+\nu )} \end{array}$$

The positive definiteness condition for tetragonal crystal class No.15 are determined as *G*_a_ > 0, *G*_s_ > 0, *K* > 0, η_t_ > 0, η_b_ > 0, γ > 0, 0 ⩽ α_a‐t_ < 1, and 0 ⩽ α_s‐b_ < 1 in terms of stiffness material parameters and as *E* > 0, −1 < ν < 1, *I* > 0, and −1 < ς < 1 in terms of compliance material parameters.

### 2.4 Couple Stress Beam Theory

Further restriction on the variation of field variables reduces the couple‐stress plate theory to the couple‐stress beam theory in 1D space. In CSBT, the beam is made of periodic tessellation of 3D unit cells in only one direction (here we consider *x*_1_), and the macroscopic fields are only functions of one coordinate in the direction of the beam. Additionally, similar to other beam theories, the planes on the boundaries perpendicular to *x*_2_ and *x*_3_ are free surfaces —that is, no force‐ and couple‐stress components act on these planes (${\hat{S}}_{i2}=0$, ${\hat{S}}_{i3}=0$, ${\hat{\mu }}_{i2}=0$, and ${\hat{\mu }}_{i3}=0$ for *i* = 1⋅⋅⋅3). Moreover, the microscopic displacement field u* varies in all three microscopic directions but is only periodic in *y*_1_. Hence, the periodic boundary conditions used in homogenization would only exist in one direction. The schematic presentation of such a 1D‐AAH is depicted in Figure 1g. It is worth mentioning that the cross‐sectional dimensions of the beam are the same as the dimensions of the unit cell (ℓ_2_ and ℓ_3_). Considering the above‐mentioned notes, the additional deformation measures (${\hat{\varepsilon }}_{12}$, ${\hat{\varepsilon }}_{22}$, ${\hat{\kappa }}_{12}$, ${\hat{\kappa }}_{22}$, and ${\hat{\kappa }}_{32}$) lose their contribution to the energy density function, and, hence, the stress measures associated with them (${\hat{\sigma }}_{12}$, ${\hat{\sigma }}_{22}$, ${\hat{\mu }}_{12}$, ${\hat{\mu }}_{22}$, and ${\hat{\mu }}_{32}$) become zero directly. Similar to the CSPT, due to the absence of periodic boundary conditions in the **y**_2_ and **y**_3_ directions in the homogenization procedure, the macroscopic displacement gradients $\partial {\hat{u}}_{2}/\partial x_{1}$ and $\partial {\hat{u}}_{3}/\partial x_{1}$ do not produce any deformation but a rigid body rotation in the unit cell (see Figure S7, Supporting Information). Consequently, the shear strains ${\hat{\varepsilon }}_{13}$ and ${\hat{\varepsilon }}_{12}$ are not included in the energy density function, and therefore their conjugate symmetric force‐stress components ${\hat{\sigma }}_{13}$ and ${\hat{\sigma }}_{12}$ are zero, that is, ${\hat{\tau }}_{2}=-{\hat{S}}_{31}$ and ${\hat{\tau }}_{3}={\hat{S}}_{21}$. Accordingly, one can also write ${\hat{\theta }}_{1}≁\hat{u}$, ${\hat{\theta }}_{2}=-{\hat{u}}_{3,1}$, and ${\hat{\theta }}_{3}=-{\hat{u}}_{2,1}$. Independence of the torsional rotation ${\hat{\theta }}_{1}$ from the displacement vector, increases the number of independent field variables to four (i.e., ${\hat{u}}_{1}$, ${\hat{u}}_{2}$, ${\hat{u}}_{3}$, and ${\hat{\theta }}_{1}$). Additionally, this will lift the restriction on the curvature to be deviatoric. The components of all the vectors and tensors associated with the CSBT are compared to those of the CST and CSPT in Table S2, Supporting Information.

#### 2.4.1 Governing Equations

A schematic representation of boundary value problem in the context of CSBT is depicted in Figure 1h, along with the components of force‐ and couple‐stresses in Figure 1i. Considering the nonzero components of force‐ and couple‐stress and by replacing **∇_x_** = (∂/∂*x*_1_, 0, 0) in Equation (10), one can write the linear and angular conservation laws in the context of CSBT in 1D space. The conservation laws and the governing equations associated with the CSBT are compared to those of the CST and CSPT in Tables S3 and S4, Supporting Information, respectively. It is worth mentioning that the governing equations of CSBT coincide with those of Euler–Bernoulli beam theory.

#### 2.4.2 Constitutive Equations

Taking into account the reduced number of macroscopic deformation measures and the nonzero components of the macroscopic strain field and solving the cell‐problem Equation (5) with only one set of periodic boundary conditions results in the derivation of the microscopic fields in terms of the nonzero macroscopic measures of deformation. Consequently, one can derive all the constitutive relations associated with CSBT by implementing Equations (7) and (8). Accordingly, the generalized elasticity tensor for 1D problem in Voigt notation becomes

(20) $$\left[\begin{array}{c} {\hat{\sigma }}_{11}\\ {\hat{\mu }}_{11}\\ {\hat{\mu }}_{21}\\ {\hat{\mu }}_{31} \end{array}\right]=\left[\begin{array}{cccc} {\bar{C}}_{11} & {\bar{B}}_{11} & {\bar{B}}_{15} & {\bar{B}}_{17}\\ & {\bar{D}}_{11} & {\bar{D}}_{15} & {\bar{D}}_{17}\\ & & {\bar{D}}_{55} & {\bar{D}}_{57}\\ & & & {\bar{D}}_{77} \end{array}\right]\cdot \left[\begin{array}{c} {\hat{\varepsilon }}_{11}\\ {\hat{\kappa }}_{11}\\ {\hat{\kappa }}_{21}\\ {\hat{\kappa }}_{31} \end{array}\right]$$

The maximum number of independent elastic components for the most comprehensive anisotropy case of CSBT is 10 (with one parameter for **C**, three for **B**, and six for **D**). Conversely, in the case of isocentral material, the fewest number of independent material parameters is three, with one parameter for **C**, none for **B**, and two for **D**.

#### 2.4.3 Beams Belonging to Chiral Tetragonal Classes No.15

The microstructured beams can not have cubic symmetry since they are periodic only in one direction. Here, we consider the regular prisms belonging to symmetry class No.15. The form of constitutive relations can be derived from those presented for CSPT. As a result, the bending modes become decoupled, and they are solely defined based on their pertinent curvature as ${\hat{\mu }}_{21}=I{\hat{\kappa }}_{21}$ and ${\hat{\mu }}_{31}=I{\hat{\kappa }}_{31}$. However, the axial stress and torsional couple‐stress are coupled, and the constitutive and compliance relations among them are

(21) $$\begin{array}{cccc} & |\begin{array}{cc} & {\hat{\sigma }}_{11}=E{\hat{\varepsilon }}_{11}+\beta_{a-t}{\hat{\kappa }}_{11}\\ & {\hat{\mu }}_{11}=\beta_{a-t}{\hat{\varepsilon }}_{11}+\eta_{t}{\hat{\kappa }}_{11} \end{array}, & & |\begin{array}{cc} & {\hat{\varepsilon }}_{11}={\hat{\sigma }}_{11}/E^{\prime }+{\hat{\mu }}_{11}/\beta_{a-t}^{\prime }\\ & {\hat{\kappa }}_{11}={\hat{\sigma }}_{11}/\beta_{a-t}^{\prime }+{\hat{\mu }}_{11}/\eta_{t} \end{array} \end{array}$$

where similar to $\eta_{t}^{\prime }$ and $\beta_{a-t}^{\prime }$ defined in Equation (16), *E*′ = *E*(1 − α_a‐t_) is the effective Young's modulus, modified by the Lake's chirality ratio $\alpha_{a-t}=\beta_{a-t}^{2}/\left(E\eta_{t}\right)$. The positive definiteness conditions for crystal class No.15 are guaranteed as *E* > 0, *I* > 0, η_t_ > 0, and 0 ⩽ α_a‐t_ < 1.

## 3 Results

In this section, we apply the theories developed throughout the paper to explore two significant phenomena in separate subsections: chirality and the Mindlin ratio. For the investigation of chirality, a chiral unit cell has been intricately tessellated to create meta‐beams and plates. We delve deep into understanding the axial‐twist coupling evident in the meta‐beams and explore both axial‐twist and shear‐bending aspects of the chiral meta‐plates. On the other hand, our focus shifts to extruded unit cells from diverse symmetry classes in the subsequent subsection. Here, the emphasis is on discerning the influence of architecture and symmetry class on the introduced Mindlin ratio.

### 3.1 Chirality

**Figure 2:**
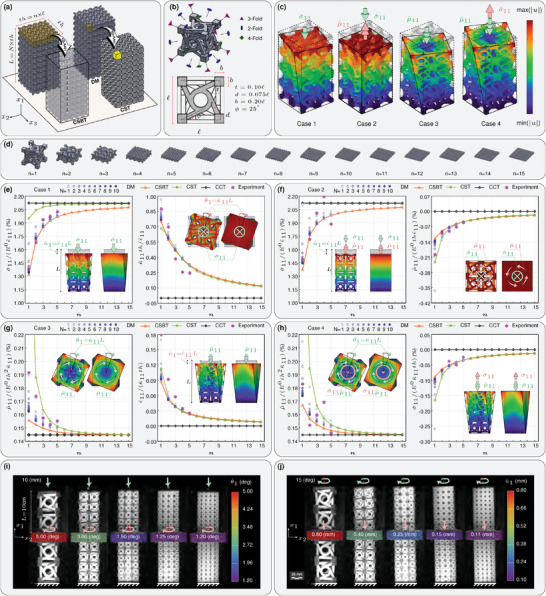
Modeling a metabeam made of a chiral metamaterial. a) Schematic representation of the models along with dimensions of the beam and the 1D and 3D RVEs. b) Architecture of the primitive chiral unit cell belonging to the crystal class No.29 (432). c) Four loading cases. d) Arrangements of primitive unit cells in 1D RVE. e–h) Comparison of the results of different models for four alternative load cases. i,j) Digital image correlation (DIC) for experimental validation of deformation for load cases (1) and (3), respectively.

**Figure 3:**
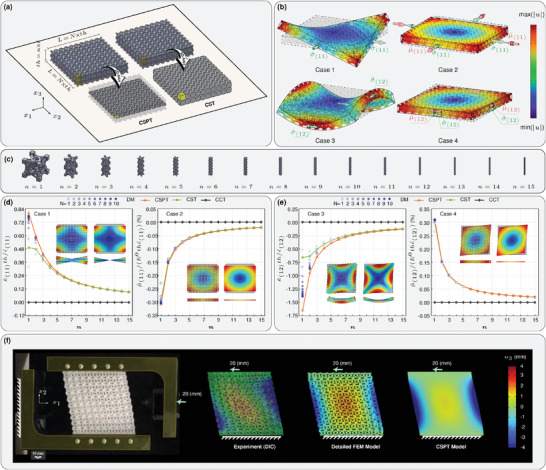
Modeling a metaplate made of a chiral metamaterial unit cell. a) Schematic representation of the models along with dimensions of the metaplate and the 2D and 3D RVEs. b) The four loading cases applied to a chiral metaplate. c) Arrangements of primitive unit cells in 2D RVE. d,e) Comparison of the results of different models for four alternative loading cases, and f) comparison of deformation of a chiral metaplate under a direct shear load, evaluated by experimentation using digital image correlation (DIC), detailed FEM model (DM), and couple‐stress plate theory.

Consider a beam (**Figure** **2**a) and plate (**Figure** **3**a) with length of *L*, thickness of *th*, and aspect ratio of *N* = *L*/*th* made of microstructured materials with chiral unit cells of size ℓ × ℓ × ℓ (Figure 2b). It is well‐known that the material characteristic lengths are in the same order as the unit cell size. Consequently, the ratio of the smallest length scale in the problem (*th*) to the material length scale (ℓ) would characterize the size effect. This ratio equals the number of unit cells fitted in the thickness *th*, *n* = *th*/ℓ (see Figures 2d and 3c). By keeping the thickness constant (*th*), we study the effect of different aspect ratios (*N*) and length‐scale ratios (*n*) on the response of chiral metabeams and ‐plates against several load cases and assess the accuracy of the proposed continuum models in comparison to the detailed modeling and experimental results as benchmarks. We approach the problems in four different ways: detailed FEM modeling (DM), an effective 3D model based on CCT, an effective 3D model based on CST, and an effective 1D and 2D model based on CSBT and CSPT.

The 3D detailed model is created in Solidworks software and imported into COMSOL Multiphysics software for the analysis. Assuming an isotropic linear elastic model for the base material, only two elastic (*E* ^0^ and ν^0^) and one inertial (ρ^0^) material parameters are required for DM. This model is the most accurate among the above models and is considered the benchmark. However, the computation cost is very high compared to the other modeling methods, and even an available high‐performance computer (HPC) with 256 GB memory and an Intel Xeon Gold 6138 CPU with 20 cores could not handle the detailed finite element modeling of metabeams with *n* greater than five when the discretization is considered fine enough to ensure accuracy. Therefore, the results of DM are reported only for *n* = 1, 2, ⋅⋅⋅, 5.

The 3D‐AAH is implemented on one primitive chiral unit cell shown in Figure 2b, made of TPU with *E* ^0^  =  50 MPa, ν^0^  =  0.49, ρ^0^  =  1085 kg m^−3^, to calculate the effective material properties required for the CST. Due to the symmetries of the unit cell, Only eight elastic and two inertia‐independent material parameters are required to characterize this metamaterial in the context of CST as presented in **Table** **1**. In the first row of Table 1, the three classic elastic parameters and the relative density are presented, all of which are scale‐independent; these material parameters are the same as the ones that can be computed through classic asymptotic homogenization (CAH). The effective material properties defined explicitly within the scope of CST are presented in the second and third rows of Table 1. In the second row, the axial‐twist and shear‐bending coupling parameters are presented, which depend linearly on the length‐scale (∝ℓ). In the third row, three elastic parameters representing the resistance of the material against curvature together with the second moment of inertia are presented, all of which are proportional to the square of the length‐scale (∝ℓ^2^). The introduced size‐dependent material parameters vanish as the unit cell size tends to zero.

**Table 1:** Effective CST material properties of the chiral unit cell shown in Figure 2e.

	Elasticity				Inertia
	Spherical	Deviatoric			
		Symm.		Anti‐Symm.	
∝/ℓ	$K^{\text{3D}}\left(\%E^{0}\right)$	$G_{a}^{\text{3D}}\left(\%E^{0}\right)$	$G_{s}^{\text{3D}}\left(\%E^{0}\right)$	−	$\rho^{\text{3D}}\left(\%\rho^{0}\right)$
	0.933	0.943	1.027		20.998
∝ℓ	−	$\beta_{a-t}^{\text{3D}}\left(\%E^{0}\ell \right)$	$\beta_{s-b}^{\text{3D}}\left(\%E^{0}\ell \right)$	−	
		−0.239	0.146	
∝ℓ^2^	−	$\eta_{t}^{\text{3D}}\left(\%E^{0}\ell^{2}\right)$	$\eta_{b}^{\text{3D}}\left(\%E^{0}\ell^{2}\right)$	$\gamma^{\text{3D}}\left(\%E^{0}\ell^{2}\right)$	$\iota^{\text{3D}}\left(\%\rho^{0}\ell^{2}\right)$
		0.365	0.220	0.359	4.303

In contrast to the 3D models, where the primitive unit cell is tessellated in three directions to build up the metamaterial and is considered the RVE, in 1D CSBT and 2D CSPT models, the building block is only tessellated in one and two directions, respectively. Therefore, the RVE includes the microstructure forming the whole cross‐section for 1D‐AAH and the whole thickness for 2D‐AAH. Consequently, the 1D‐ and 2D‐AAH must be implemented for each value of *n*. The effective material properties determined by conducting 1D‐ and 2D‐AAH are presented in Section S6.1, Supporting Information, for *n* varying from one to 15. It is observed that as *n* increases, the effective CSBT and CSPT material properties converge to the properties of a beam and a plate in the context of classical Euler–Bernoulli and Love–Kirchhoff plate theory, made of a material with properties computed by the application of CAH on the chiral unit cell, respectively.

#### 3.1.1 Metabeam

To compare the introduced numerical models and verify their outputs with the experimental investigation, we examine four unique load cases applied to a chiral metabeam. These cases involve two loading conditions in combination with two boundary conditions. In all instances, the bottom face of the beam is fixed against displacement and rotation, while a relatively rigid plate is attached to the top face (see Figure S15, Supporting Information). In the first two load cases, axial strain is applied to the beam by displacing the top face toward the bottom face, while the top face can be free (Case 1) or fixed (Case 2) against rotation. In the subsequent two load cases, an axial curvature is applied by rotating the top face, while again, the top face can be free (Case 3) or fixed (Case 4) against axial displacement (Figure 2c).

Figure 2e–h depicts dimensionless stresses (${\hat{\sigma }}_{11}$ and ${\hat{\mu }}_{11}$) and deformations (${\hat{\varepsilon }}_{11}$ and ${\hat{\kappa }}_{11}$) induced in the beam due to the application of load cases (1) to (4) for different values of *n*, respectively. Each figure contains two sub‐figures: the left‐side sub‐figure depicts the stress pertinent to the applied deformation, while the right hand‐side sub‐figure illustrates the stress (force or couple) or deformation (strain or curvature) induced due to the chirality of the material. Moreover, each sub‐figure compares the results calculated by using the four methods introduced earlier, that is, DM, CCT, CST, and CSBT. The load cases and the dimensionless results are chosen in such a way that the aspect ratio does not play a role in the results of effective CSBT, CST, and CCT models. However, as depicted in all of the aforementioned figures, for each value of *n*, as the aspect ratio (*N*) increases, the results of DM converge to those of CSBT, which is expected to be the most accurate model for a beam. This convergence is due to the satisfaction of the one‐directional periodic boundary condition assumed in 1D‐AAH. It is observed that for larger values of *n* the required aspect ratio to achieve a relatively accurate result decreases, which is due to the fact that the actual number of 1D RVEs in the beam is equal to *n* × *N*. Assuming the number of required 1D‐RVEs to achieve a desired accuracy to be constant, the required *N* becomes smaller as *n* increases.

In the first two cases (Figure 2e,f), a beam made of the chiral metamaterial is compressed on the top face and undergoes axial compressive strain. Implementing Equation (21), the induced stress for Cases 1 and 2 are ${\hat{\sigma }}_{11}/{\hat{\varepsilon }}_{11}=E^{\text{1D}}\left(1-\alpha_{\text{a-t}}\right)$ and ${\hat{\sigma }}_{11}/{\hat{\varepsilon }}_{11}=E^{\text{1D}}$, respectively. Due to the absence of a center of symmetry, the beam intends to twist (${\hat{\kappa }}_{11}/{\hat{\varepsilon }}_{11}=-\beta_{\text{a-t}}^{\text{1D}}/\eta_{t}^{\text{1D}}$) when it is free to rotate on its top (Case 1), while otherwise, it requires a torque (${\hat{\mu }}_{11}/{\hat{\varepsilon }}_{11}=\beta_{\text{a-t}}^{\text{1D}}$) at the resisting support on the top (Case 2). As $\beta_{\text{a-t}}^{\text{1D}}<0$, the beam twists in the same direction as the applied load in Case 1, while in Case 2, the resisting torque has the opposite direction. Therefore, in these examples, since the strain is negative (compression) the curvature is also negative, and the resisting twist is positive. In both load cases, as the value of *n* increases, the dimensionless averaged axial force‐stress required to induce axial strain (which represents the effective 1D Young's modulus) also increases. This 1D Young's modulus is larger for the case where the top face is fixed against rotation (Case 2) compared to that of Case 1 since the Lake's chirality measure (α_a‐t_) is always greater than or equal to zero.

In the following two instances (Figure 2g,h), we observe the twisting of the same beam along its top face while an axial twisting curvature is applied. By utilizing Equation (21), we can calculate the twisting couple‐stress induced in Cases 3 and 4 as ${\hat{\mu }}_{11}/{\hat{\kappa }}_{11}=\eta_{t}^{\text{1D}}\left(1-\alpha_{\text{a-t}}\right)$ and ${\hat{\mu }}_{11}/{\hat{\kappa }}_{11}=\eta_{t}^{\text{1D}}$, respectively. Notably, when the top face is allowed to vertically displace, the same phenomenon responsible for inducing curvature in response to axial stress also generates axial strain (${\hat{\varepsilon }}_{11}/{\hat{\kappa }}_{11}=-\beta_{\text{a-t}}^{\text{1D}}/E^{\text{1D}}$) with the same sign as the applied twist. Conversely, if the top face is fixed against displacement, an axial stress (${\hat{\sigma }}_{11}/{\hat{\kappa }}_{11}=\beta_{\text{a-t}}^{\text{1D}}$) with an opposite sign is necessary.

Since the CCT is size‐independent, the predicted force‐stress required to compress the beam, and the couple‐stress needed to twist it remains constant when the microstructure size is changing. Additionally, chirality is not recognized in CCT, and no coupling effect is captured. On the other hand, CST involves size‐dependent material parameters and recognizes chiral symmetries. CST can effectively predict the coupling effects resulting from chirality; however, it faces challenges in accurately capturing the stiffening behavior of the beam under compression and its softening behavior under torsion. This limitation stems from the assumption of three‐directional periodicity in the associated 3D‐AAH.

In Case 2, where the curvature is not permitted due to boundary conditions, the stress–strain relationship in CST is the same as that in CCT and is size‐independent. In Case 1, where the beam is free to rotate at the top face, the inclusion of the coupling effect in CST results in a lower effective stress compared to the prediction of CCT. However, as the size of the unit cell decreases, this effect diminishes, and a stiffening behavior is observed. A similar explanation is valid for the case of torsion, except that CST predicts much higher torsional stiffness than CSBT. It is due to the fact that the difference between the torsional stiffness of the unit cell with and without a periodic boundary condition is much higher than that of the axial stiffness. Clearly, two contradictory criteria are competing in the effectiveness of the CST model. On the one hand, the material is required to satisfy the periodicity condition, that is, *n* tends to infinity, to allow the CST model to be accurate. On the other hand, the size effect is much more considerable when the ratio of the metamaterial length scale (ℓ) to the smallest macroscopic length scale (*th*) is minimum, that is, *n* is equal to one. By increasing the value of *n*, the accuracy of CST increases, and at the same time, the size effect disappears. To address this challenge, we have introduced CSBT. In this model, the metamaterial length scales can be comparable to the metabeam length scales (i.e., cross‐sectional dimensions of the beam), while the accuracy of the beam model can be ensured by increasing the number of RVEs in the axial directions (*n* × *N*).

In addition to the above‐mentioned adopted numerical analyses, experimental tests have also been conducted, and the results are presented in Figure 2. For loading cases 1 and 3, the associated forces and torsional moments are captured using the testing machine, while for loading cases 2 and 4 the coupled deformations are captured using digital image correlation (DIC, Figure 2i,j). As observed in Figure 2, the experimental results corroborate the homogenization‐based and detailed numerical predictions.

#### 3.1.2 Metaplate

Similar to the study performed in the previous section for chiral metabeams, we examine four unique load cases applied to a chiral metaplate. In the first two load cases, a unit deviatoric strain is applied to the plate by displacing the edges of the plate, while the edges are free to displace out‐of‐plane (Case 1) or fixed against it (Case 2). In the subsequent two load cases, a shear strain is applied by displacing the edges, while the edges can be free (Case 3) or fixed (Case 4) against out of plane displacements (Figure 3b). These load cases were chosen because they allow for straightforward solutions to the partial differential equations (PDEs) of the couple stress plate theory.

Figure 3d,e depicts dimensionless stresses and deformations induced in the metaplate due to the application of load cases (1) to (4) for different values of *n* (see Figure 3b). Each figure contains two sub‐figures: the left‐side sub‐figure depicts the induced curvature when the plate is free to deform out‐of‐plane, while the sub‐figure on the right side illustrates the couple‐stress induced due to the chirality of the metamaterial at the boundaries. Moreover, each sub‐figure compares the results obtained using DM, CSPT, CST, and CCT. Similar to the cases studied for the metabeam, the load cases and the dimensionless results are chosen in such a way that the aspect ratio (*N*) does not play a role in the results of effective models (CSPT, CST, and CCT). It is shown that for each value of *n*, as the aspect ratio (*N*) increases, the results of DM converge to that of CSPT. This convergence is due to the satisfaction of the two‐directional periodicity adopted in 2D‐AAH. For larger values of *n*, the required aspect ratio to achieve a relative accurate result decreases, which is due to the fact that the actual number of 2D RVEs in the plate is equal to *n* × *N* × *N*. Assuming the number of required 2D‐RVEs to achieve the desired accuracy to be constant, the required *N* becomes smaller as *n* increases. For example, when *n* = 1, the required *N* to have an accurate result is almost *N* = 10, while the result of *n* = 5 is even more accurate when *N* = 1.

In the first two loading cases (Figure 3d), a chiral metamaterial metaplate undergoes compression on the top and bottom faces while being extended from the left and right faces, resulting in a deviatoric biaxial strain. As explained in the theory section, only the deviatoric part of strain and force‐stress couples to the deviatoric curvature and couple‐stress. Therefore, we only apply the deviatoric strain to the metaplate. By implementing the constitutive law for chiral metaplates introduced earlier (Equation (14) with indices ranging from 1 to 2), the metaplate tends to twist (${\hat{\kappa }}_{\left(11\right)}/{\hat{\varepsilon }}_{\left(11\right)}=-\beta_{\text{a-t}}^{\text{2D}}/\eta_{t}^{\text{2D}}$) in the same direction as the applied load when the faces are free to rotate (Case 1). Alternatively, it induces a torque (${\hat{\mu }}_{\left(11\right)}/{\hat{\varepsilon }}_{\left(11\right)}=\beta_{\text{a-t}}^{\text{2D}}$) with the opposite direction of the applied load at the resisting supports (Case 2). The induced torsional curvature and couple‐stress associated with load cases 1 and 2 are plotted in Figure 3d.

In the subsequent two cases, the same metaplate is subjected to shear deformation on the boundaries. Again, by utilizing the relevant constitutive laws, the metaplate bends in an anticlastic shape (${\hat{\kappa }}_{\left(12\right)}/{\hat{\varepsilon }}_{\left(12\right)}=\beta_{\text{s-b}}^{\text{2D}}/\eta_{b}^{\text{2D}}$) with an opposite sign to the applied load when the faces are free to rotate (Case 3). Conversely, when the resisting supports prevent rotation, it induces symmetric bending (${\hat{\mu }}_{\left(12\right)}/{\hat{\varepsilon }}_{\left(12\right)}=\beta_{\text{s-b}}^{\text{2D}}$) with the same sign as the applied load (Case 2). Please refer to Figure 3e for detailed illustrations of these cases.

As expected, chirality is not considered in CCT, resulting in no curvature or couple‐stress being induced in the metaplate under a biaxial load. However, CST accounts for size dependence and properly acknowledges chiral symmetries. Similar to the metabeam case, CST successfully predicts deformations and forces resulting from couplings. Nevertheless, for accurate stiffness prediction and to satisfy periodic boundary conditions, a sufficiently high number of unit cells (*n*) in the plate's thickness is required. On the other hand, CSPT is specifically developed for scenarios where periodic boundary conditions exist in two directions. Figure 3d,e demonstrates that CSPT accurately models the behavior of chiral metaplates.

This article presents, for the first time, the analysis of the out‐of‐plane deformation of a metaplate undergoing shear deformation. To validate the proof of concept, an experimental study was conducted on the chiral metaplate shown in Figure 3. One side of the metaplate was clamped, while a shear deformation was applied to the other side using specially designed and 3D printed parallel rigid grips capable of off‐axis relative movements. The out‐of‐plane deformation of the samples was measured using the digital image correlation (DIC) technique and compared with the predictions obtained from the FEM solution of detailed and effective CSPT models, as shown in Figure 3f.

It is imperative to note that while the FEM solution of the CSPT model provided notable insights into the out‐of‐plane deformation trends of the chiral metaplate, certain disparities in the maximum *u*_3_ values were observed when juxtaposed with the detailed FEM model. This discrepancy originates from the usage of the quadratic Lagrange shape functions in the preliminary FEM model based on CSPT, which do not satisfy the requisite *C* ^1^ continuity for the developed theory.^[^ ^48^, ^49^ ^]^

### 3.2 Mindlin Ratio

In the context of classical Love–Kirchhoff plate theory, a homogeneous plate made of regular non‐auxetic (ν > 0) materials under a bending moment in one direction deforms into a saddle‐like (anticlastic) shape. In contrast, a plate made of auxetic materials (ν < 0) bends like a dome (synclastic) shape. This classification has been generalized to microstructured materials like extruded honeycombs.^[^ ^50^, ^51^ ^]^ In this paper, within the framework of CSPT, we have introduced a novel material parameter called Mindlin's ratio (ς), which governs the formation of double curvature in bending plates. When ς > 0, the plate exhibits a saddle‐like (anticlastic) shape, while ς < 0 corresponds to a dome‐like (synclastic) shape.

**Figure 4:**
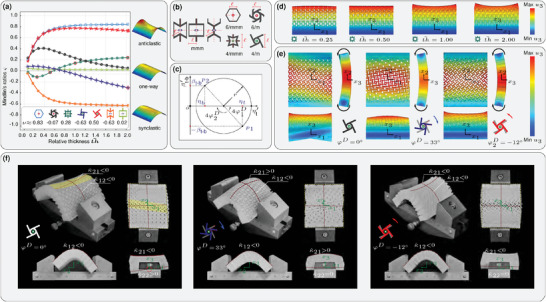
Mindlin's ratio in metaplates. a) Variation of Mindlin's ratio in metaplates with relative thickness ($\bar{th}=th/\ell$) for various extruded unit cells. b) Architecture of the primitive unit cells and their symmetries considered to form metaplates (more detail on the architecture of the selected unit cells is available in Figure S12, Supporting Information). c) Mohr's‐circle for variation of bending‐torsion coupling with the transformation angle (φ). d) Detailed FEM for the unidirectional bending of metaplates composed of 10 × 10 of auxetic star‐shaped (4/*mmm*) unit cells with alternative relative thicknesses. e) Detailed FEM analysis results and f) experimental validation of the deformation behavior during unidirectional bending of 3D‐printed samples comprising 10 × 10 2D‐tetrachiral (4/*m*) unit cells, with a side‐length of ℓ = 1 cm and thickness $\bar{th}=2$, for different orientations.

To investigate Mindlin's ratio, we employed the 2D‐AAH method on unit cells belonging to five different symmetry classes, namely No.21 (6/*mmm*), No.24 (6/*m*), No.14 (4/*mmm*), No.17 (4/*m*), and No.6 (*mmm*), as illustrated in **Figure** **4**b. The constitutive relations associated with these symmetry classes can be found in Section S5.2, Supporting Information. Despite each unit cell having a nearly constant Poisson's ratio, the evaluation of Mindlin's ratio using the 2D‐AAH method reveals its dependence on the relative thickness ($\bar{th}=th/\ell$) of the plate, as shown in Figure 4a. This observation challenges the prevailing understanding, which associates transverse curvature directly with Poisson's ratio. Notably, as the relative thickness of the plate approaches infinity, Mindlin's ratio tends to Poisson's ratio, indicating a size dependency in CSPT.

The distinction between the prediction of double curvature using Poisson's and Mindlin's ratios becomes more pronounced in the case of star re‐entrant (4/*mmm*), 2D‐hexachiral (6/*m*), and 2D‐tetrachiral (4/*m*) unit cells, where even the signs of Mindlin's and Poisson's ratios differ for specific relative thicknesses. For instance, the Poisson's ratio of the star re‐entrant unit cell is approximately 0.28. However, for $\bar{th}=0.25$, 0.50, 1.00, and 2.00, the corresponding Mindlin's ratios are evaluated as ς = −0.11, −0.03, 0.14, and 0.26, respectively. To further illustrate the deformation behavior, Figure 4d displays a transverse view of the deformed shape of metaplates composed of star re‐entrant unit cells, obtained through a detailed FEM analysis. These deformations align with Mindlin's ratios, determined by employing the developed 2D‐AAH method.

The absence of in‐plane mirror symmetries in the (6/*m*) and (4/*m*) symmetry classes prevents the occurrence of chirality in these materials. Consequently, there is no coupling between force‐ and couple‐stresses within these materials. However, despite the absence of chiral properties, these materials are often referred to as 2D‐hexa‐ and 2D‐tetrachiral in the literature due to the lack of in‐plane mirror symmetries.

Within the (4/*m*) symmetry class, which characterizes the 2D‐tetrachiral unit cell, two intriguing couplings exist: one between shear and deviatoric stresses (β_a‐s_), and another between torsion and symmetric bending couple‐stress (β_t‐b_). In Section S5.2.2, Supporting Information, it is demonstrated that these couplings vanish after a right‐handed rotation (φ) of the axes around the *x*_3_‐axis. A Mohr's circle associated with this coordinate transformation is depicted in Figure 4c, illustrating the disappearance of the coupling between torsion and symmetric bending couple‐stress (β_t‐b_). Specifically, rotating the coordinates by $\varphi_{1}^{D}>0$ or by $\varphi_{2}^{D}<0$ around the *x*_3_‐axis ($\varphi_{1}^{D}-\varphi_{2}^{D}=\pi /4$) eliminates this coupling. It should be noted that rotating the coordinates by φ around an axis is equivalent to rotating the unit cell by −φ around the same axis. Figure 4a presents the variation of Mindlin's ratio for each principal orientation versus the relative thickness. It is noteworthy that the principal directions differ at each relative thickness.

The deformation of metaplates composed of 10 × 10 2D‐tetrachiral unit cells, with a side‐length of ℓ = 1 cm and thickness $\bar{th}=2$, arranged in three different directions (φ^*D*^ = 0°, 33°, −12°) under unidirectional bending, is determined using a detailed FEM model, as shown in Figure 4e. Additionally, 3D‐printed samples with the same dimensions are systematically placed in a gripper to experimentally validate the predicted deformations under unidirectional bending, as depicted in Figure 4f. The results demonstrate that when φ^D^ = 0, the plate undergoes twisting during unidirectional bending, while it exhibits anticlastic and synclastic shapes for φ^*D*^ = 33° and φ^D^ = −12°, respectively, which complies with the values of ς determined using 2D‐AAH in the context of CSPT. See Section S6.3, Supporting Information for a comparison of the double curvature ratio in 2D‐tetrachiral metaplates using 2D‐AAH, FEM simulations, and experimental data. Additional detailed results regarding the variation of Mindlin's ratio can be found in Section S6.2, Supporting Information.

## 4 Conclusion

The remarkable multifunctional properties exhibited by mechanical metamaterials can be attributed to their intricate underlying architectures. Traditionally, the rational design of metamaterials with exceptional properties has relied on intuition and inspiration from nature^[^ ^52^, ^53^ ^]^ or ancient arts.^[^ ^54^, ^55^ ^]^ However, in order to enable a systematic approach for tailoring the properties of metamaterials, a comprehensive understanding of their classification and size‐dependent characteristics is essential. This study has primarily focused on exploring these key aspects, aiming to provide insights into the systematic design of metamaterials rather than relying solely on intuition or inspiration.

For this purpose, we have developed a consistent and self‐sufficient couple‐stress theory by implementing a bottom‐up approach based on augmented asymptotic homogenization for mechanical metamaterials. The inconsistency problem in ICST is effectively resolved by demonstrating the deviatoric nature of the couple‐stress tensor aligning with the homogenization results. Unlike the MT, which introduces a micro‐rotation vector as an additional degree of freedom to the macroscopic displacement vector, in CST, the rotation field is determined by the curl of the macroscopic displacement vector, which simplifies the application of boundary conditions. Furthermore, while the micropolar theory incorporates rotation and the antisymmetric part of stress in the energy density function and constitutive relations, the antisymmetric part of stress in the couple‐stress theory is derived from the conservation of angular momentum (couple equilibrium). This key distinction becomes evident in the current investigation when we reduce the CST to the CSPT and CSBT, where the out‐of‐plane shear is also determined from couple equilibrium, analogous to classical beam and plate theories. These models are able to completely classify mechanical metamaterials and ‐plate and ‐beams based on the symmetry class of their RVE and are capable of realizing non‐centrosymmetric behavior in these architected materials/structures.

Our introduced methodology provides the appropriate size‐dependent models for metamaterial and determines their unique and reproducible effective material properties that can be used for systematic computational characterization of their functionalities. The accuracy of these models has been examined by comparing them with detailed finite element modeling and experimentation on 3D printed samples. It is worth mentioning that the observed continuum‐based size‐effects for metamaterials/structures are inherited from their underlying material architecture and geometrical boundary conditions. Incorporating base material size‐effect^[^ ^56^, ^57^ ^]^ in addition to geometrical size‐effect^[^ ^24^ ^]^ for developing metamaterials in multiple hierarchical scales may require the adoption of atomistic modeling^[^ ^58^, ^59^, ^60^ ^]^ rather than a continuum mechanics approach. Implementing these models opens new avenues for designing mechanical metamaterials with programmable couplings. As examples, we have studied a few symmetry classes using the proposed models, introducing new material properties that characterize axial‐twist, shear‐bending, bending‐twist, and double curvature bending couplings. The mathematical analysis of the derived constitutive laws pertinent to cubic chiral metamaterials shows that the symmetric part of bending couple‐stress is coupled with shear deformation, and its antisymmetric part does not produce any coupling. Similarly, it is shown that the deviatoric part of axial stress and strain is coupled with the twist, while the spherical (hydrostatic) part does not produce any twist. The axial‐twist coupling in a chiral metabeam has been characterized; it is shown that this class of metabeams not only twists when compressed (or extended) but also compresses (or extends) when twisted. The out‐of‐plane deformation of a metaplate under in‐plane forces has been realized, while it was prohibited in the Cauchy continuum theory. We showed that a chiral metaplate made of an isotropic base material twists when compressed/extended and bends when subjected to a shear load, and vice versa.

The double‐curvature shape of plates under unidirectional bending was previously linked to Poison's ratio. In this study, we have introduced Mindlin's ratio, which solely governs the ratio of the curvatures and specifies the synclastic and anticlastic shapes of a metaplate under bending. At last, metaplates with a so‐called 2D‐tetrachiral architecture have been studied in the context of CSPT. It has been shown that these metaplates demonstrate a coupling between twist and bending couple‐stresses in addition to the shear‐axial coupling. Using a coordinate transformation and its associated Mohr's circle, we have found two distinctive sets of rotation angles along which these couplings disappear, and two pure bending shapes (one synclastic and the other anticlastic) appear.

In conclusion, we deem this study imparts a self‐sufficient continuum approach for the systematic design of materials with an engineered microstructure that offers on‐demand coupled/uncoupled elastic properties to avoid/reduce trial‐and‐error experimentation and costly numerical approaches, accelerating mechanical metamaterial discovery. Despite the long‐lasting history of chiral materials, they are still valuable and controversial challenges in mechanics and optics. Providing a generalized continuum approach can create a bridge between mechanical properties and their counterpart optical characteristics.^[^ ^27^, ^29^, ^61^ ^]^ In particular, the suggested theories, when coupled with appropriate augmented homogenization methods, enable the modeling of acoustic activity. Moreover, the metaplates and metabeams demonstrate programmable shape morphing characteristics suitable for applications in designing 3D flexible electronic devices,^[^ ^62^ ^]^ human body joint‐aiding supports for biomedical devices,^[^ ^51^ ^]^ waveguiding metamaterials,^[^ ^63^ ^]^ and reconfigurable robotic arms.^[^ ^64^, ^65^ ^]^ Another practical application of the homogenization scheme presented in this study pertains to the field of civil engineering. For decades, cellular beams such as honeycomb steel girders and circular/box voided slabs^[^ ^66^ ^]^ have been prevalent. By employing the methods introduced in this study, it is possible to precisely ascertain the effective beam and plate properties of these structures, streamlining the modeling process without compromising accuracy.

## 5 Experimental Section

To experimentally validate the proposed models for mechanical metamaterials, 3D printed specimens were manufactured using a selective laser sintering (SLS) printer and thermoplastic polyurethane (TPU) material. To provide the necessary boundary conditions during the experiments, specific fixtures were designed and produced using a fused deposition modeling 3D printer and thermoplastic filaments. To accurately capture deformations, 3D DIC techniques were utilized. Additionally, forces were measured using a 20 kN dual‐column ADMET eXpert 8612 universal testing machine (Section S7, Supporting Information, provides further details).

## Conflict of Interest

The authors declare no conflict of interest.

## Author Contributions

S.E. and A.H.A. designed the research; S.E. performed the theoretical and computational studies; S.E., B.S., and A.H.A. designed the experimental tests and set‐up and contributed to prototyping; S.E. analyzed computational data; B.S. conducted the experimentation; B.S. and S.E. analyzed the experimental data; S.E. and B.S. conducted the research under the supervision of A.H.A.; S.E. wrote the draft of the theoretical and computational sections; B.S. wrote the draft of the experimental section; and all authors contributed to detailed reviewing and editing of the paper.

## Supporting information

Supporting Information

Supplemental Movie 1

Supplemental Movie 2

Supplemental Movie 3

Supplemental Movie 4

Supplemental Movie 5

Supplemental Movie 6

Supplemental Movie 7

Supplemental Movie 8

Supplemental Movie 9

Supplemental Movie 10

Supplemental Movie 11

## Data Availability

The data that support the findings of this study are available in the supplementary material of this article.
