# Supplementary material for: Unravelling Size‐Dependent and Coupled Properties in Mechanical Metamaterials: A Couple‐Stress Theory Perspective
Source: Adv Sci (Weinh). 2024 Jan 2;11(13):2305113. doi: 10.1002/advs.202305113 (PMC10987119; doi:10.1002/advs.202305113)
Supplement: Supplementary file 1 — Supporting Information [file ADVS-11-2305113-s008.pdf]

ADVANCED SCIENCE
Open Access

Supporting Information

for *Adv. Sci.*, DOI 10.1002/advs.202305113

Unravelling Size-Dependent and Coupled Properties in Mechanical Metamaterials: A Couple-Stress Theory Perspective

*Shahin Eskandari, Benyamin shahryari and Abdolhamid Akbarzadeh**

Supporting Information

Unravelling Size-dependent and Coupled Properties in Mechanical Metamaterials: A Couple-Stress Theory Perspective

Shahin Eskandari, Benyamin Shahryari and Abdolhamid Akbarzadeh*

Department of Bioresource Engineering, McGill University, Island of Montreal, QC, H9X 3V9, Canada

*Corresponding author: Hamid Akbarzadeh E-mail: hamid.akbarzadeh@mcgill.ca

This PDF file includes:

S1. Bottom-up construction of couple-Stress theory (CST) from Cauchy Elasticity
S2. Augmented asymptotic homogenization (AAH)
S3. Symmetries of Generalized Elasticity Tensors
S4. Voigt notation
S5. Constitutive and compliance relations
S6. Supplementary results
S7. Experiments

Supplementary Figures S1 to S18

Supplementary Tables S1 to S9

Legends for Supplementary Movies S1 to S11

Supplementary Information References

Other supplementary materials for this manuscript include the following:

Movies S1 to S11

S1 Bottom-up construction of couple-Stress theory (CST) from Cauchy Elasticity

Asymptotic homogenization (AH) is a well-developed technique with a strong mathematical basis, which has primarily been proposed for studying partial differential equations with rapidly oscillating coefficients [3, 24]. This method has been successfully applied to composites and cellular materials [8, 10, 14] to calculate material properties required to map such media to Cauchy continuum model. This method is closely related to double scale perturbation analysis of partial differential equations. In this section, we revisit the necessary formulation for application of AH on a general elasticity problem and construct our formulation based on the one presented by Guedes et al. [8].

S1.1 Problem statement

Let Ω be an open subset of $\mathbb{R}^3$ with a smooth boundary Γ made of a crystalline material with periodic microstructure (Refer to Main text, Fig.1 (a)). Let the primitive unit cell Y be a parallelepiped in $\mathbb{R}^3$ defined by three lattice vectors $\boldsymbol{\ell}_j$ $(j = 1, 2, 3)$. We consider ¥ as the solid part of the unit cell, while s denotes the interior boundaries of the unit cell. Assuming the infinitesimal deformation, the local conservation equation of linear momentum in Cauchy elasticity governs the deformation of Ω as follows [11]

$$\boldsymbol{\sigma}.\boldsymbol{\nabla} + \mathbf{f} = \dot{\mathbf{L}} \tag{S1}$$

where $\mathbf{L}$, $\boldsymbol{\sigma}$, and $\mathbf{f}$ are the linear momentum, symmetric Cauchy stress, and applied body force, and $\boldsymbol{\nabla}$ is the vector differential operator. Moreover, dot denotes the scalar product, and over-dot represents differentiation with respect to time. Unless specified otherwise, bold symbols represent vectors and tensors, and italic symbols correspond to scalar parameters. We restrict our attention to the body forces described by potential functions as $\mathbf{f} = \boldsymbol{\nabla}\Phi_f + \boldsymbol{\nabla} \times \boldsymbol{\Psi}_f$, where Φ_f and $\boldsymbol{\Psi}_f$ are prescribed scalar and vector body force potentials. The scalar potential produces curl-free, and conservative body forces, and vector potential applies divergence-free, non-conservative body forces. The corresponding local constitutive relations are

$$\boldsymbol{\sigma} = \mathbf{C}^\circ : \boldsymbol{\epsilon}, \tag{S2a}$$

$$\mathbf{L} = \rho^\circ\, \mathbf{v}, \tag{S2b}$$

where $\mathbf{C}^\circ$ and ρ° are, respectively, the fourth-order elasticity tensor with all the major and minor symmetries ($C^\circ_{ijkl} = C^\circ_{klij} = C^\circ_{jikl} = C^\circ_{ijlk}$) and mass density of the base material; $\mathbf{u}$, $\mathbf{v}$ and $\boldsymbol{\epsilon}$ are the displacement vector, velocity vector and strain tensor. In the infinitesimal deformation regime, the displacement gradient $\mathbf{F}$ may be decomposed into its symmetric ($\boldsymbol{\epsilon}$) and antisymmetric parts ($\boldsymbol{\varphi}$). The antisymmetric part of displacement gradient represents the rotation field in the body and has three independent components, and can be represented by an axial vector [1] ($\boldsymbol{\theta}$). The measures of infinitesimal deformation are summarized below

$$\mathbf{F} = \mathbf{u}\,\boldsymbol{\nabla}, \tag{S3a}$$

$$\boldsymbol{\epsilon} = \mathbb{S} : \mathbf{F}, \tag{S3b}$$

$$\boldsymbol{\varphi} = \mathbb{W} : \mathbf{F}, \tag{S3c}$$

$$\boldsymbol{\theta} = -\frac{1}{2}\boldsymbol{\varepsilon} : \boldsymbol{\varphi} = \frac{1}{2}\nabla \times \mathbf{u}, \tag{S3d}$$

in which $\mathbb{S} = 1/2\,(\mathbb{I} + \mathbb{T})$ and $\mathbb{W} = 1/2\,(\mathbb{I} - \mathbb{T})$ are the forth-order symmetric and antisymmetric tensors; $\mathbb{I}$ and $\mathbb{T}$ are the fourth-order identity tensors ($\mathbf{A} = \mathbb{I} : \mathbf{A}$, $\mathbf{A}^T = \mathbb{T} : \mathbf{A}$); $\boldsymbol{\varepsilon}$ is the Levi-Civita permutation tensor. The problem of finding the deformation of a body Ω subjected to the body force $\mathbf{f}$, traction $\mathbf{t}_n$ on the boundary Γ_t together with traction $\mathbf{p}$ on the interior boundaries s, and prescribed displacement $\mathbf{u}_\mathrm{r}$ on Γ_d in weak-form can be stated as

$$\int\limits_{\Omega} (\boldsymbol{\delta}\mathbf{u}\,\boldsymbol{\nabla} : \mathbf{C}^\circ : \mathbf{u}\,\boldsymbol{\nabla} + \rho^\circ\, \boldsymbol{\delta}\mathbf{u} \cdot \dot{\mathbf{v}})\,\mathrm{dV} = \int\limits_{\Gamma_\mathrm{t}} \boldsymbol{\delta}\mathbf{u} \cdot \mathbf{t}_n\,\mathrm{dA} + \int\limits_{\mathrm{s}} \boldsymbol{\delta}\mathbf{u} \cdot \mathbf{p}\,\mathrm{dA} + \int\limits_{\Omega} \boldsymbol{\delta}\mathbf{u} \cdot \mathbf{f}\,\mathrm{dV} \tag{S4}$$

where $\boldsymbol{\delta}\mathbf{u}$ is an arbitrary admissible virtual displacement field taking zero value on the boundary Γ_d. A unique solution $\mathbf{u}$ exists for the (S4) under the assumption that the functions $\mathbf{f}$, $\mathbf{t}_n$, and $\mathbf{p}$ are sufficiently smooth, and the boundaries Γ_t, Γ_d, and s are regular. Solving such a problem using finite element methods is possible; however, since the discretization of the body in order to represent detailed microstructure becomes a cumbersome task, it is desirable to develop a method that can reflect the microstructure without looking at details of all material points of the body, whenever the macroscopic behavior of the body is concerned. To capture the effective macroscopic behavior of materials made of a spatial repetition of a unit cell with arbitrary constituents as depicted in Main text, Fig.1 (c), we implement the asymptotic homogenization method.

[1] axial vector or Pseudo-vector is an array of quantities that transforms like a vector under an orientation-preserving coordinate transformation, e.g. a proper rotation, but changes sign under an orientation reversing coordinate transformation, e.g. reflection.

S1.2 Asymptotic expansion

Two explicit assumptions are made in the AH theory: (1) the microstructure is spatially periodic, and (2) variation of all the fields can be approximated by their variation on multiple spatial scales due to the existence of a microstructure, including slow variation in terms of macroscopic coordinate $\mathbf{x}$, and periodic rapid variation in terms of microscopic coordinate $\mathbf{y}$. Therefore, all the relevant field variables are approximated by an asymptotic expansion

$$\boldsymbol{\Lambda}[\mathbf{r}] = \bar{\boldsymbol{\Lambda}}[\mathbf{x},\mathbf{y}] + \eta\boldsymbol{\Lambda}^1[\mathbf{x},\mathbf{y}] + \eta^2\boldsymbol{\Lambda}^2(\mathbf{x},\mathbf{y}) + \cdots, \tag{S5}$$

where $\boldsymbol{\Lambda}$ is the total value of the field variable, $\bar{\boldsymbol{\Lambda}}$ is the macroscopic part of the field variable, $\boldsymbol{\Lambda}^1$, $\boldsymbol{\Lambda}^2$, etc. are perturbations in the field variables due to existence of the microstructure that are periodic with respect to $\mathbf{y}$; η is the ratio of the microstructure length scale to the macroscopic length scale; $\mathbf{r}$ is the actual coordinate system. In manipulating the spatial derivatives of the double (macro and micro) scale functions, the following chain rule is useful

$$\nabla_{\mathbf{r}} = \nabla_{\mathbf{x}} + \frac{1}{\eta}\nabla_{\mathbf{y}} \tag{S6}$$

and that, for a Y-periodic function $\boldsymbol{\Upsilon}[\mathbf{r}] = \boldsymbol{\Upsilon}[\mathbf{r}+\mathbf{Y}]$

$$\lim_{\eta\to0^+}\int\limits_{\Omega}\boldsymbol{\Upsilon}[\mathbf{r}]\mathrm{d}\Omega = \int\limits_{\Omega}\frac{1}{|\mathrm{Y}|}\int\limits_{\yen}\boldsymbol{\Upsilon}[\mathbf{x},\mathbf{y}]\mathrm{d}\yen\ \mathrm{d}\Omega, \tag{S7a}$$

$$\lim_{\eta\to0^+}\eta\int\limits_{\mathrm{S}}\boldsymbol{\Upsilon}[\mathbf{r}]\mathrm{dS} = \int\limits_{\Omega}\frac{1}{|\mathrm{Y}|}\int\limits_{\mathrm{S}}\boldsymbol{\Upsilon}[\mathbf{x},\mathbf{y}]\mathrm{dS}\ \mathrm{d}\Omega, \tag{S7b}$$

where $|\mathrm{Y}|$ stands for the volume of the unit cell. Inserting the asymptotic expansion (S5) for the displacement and velocity vectors into (S4), and using the chain rule introduced in (S6), the functional becomes

$$\int\limits_{\Omega}\left[\boldsymbol{\delta}\mathbf{u}\left(\nabla_{\mathbf{x}} + \frac{1}{\eta}\nabla_{\mathbf{y}}\right):\mathbf{C}^{\circ}:(\bar{\mathbf{u}} + \eta\mathbf{u}^1 + \cdots)\left(\nabla_{\mathbf{x}} + \frac{1}{\eta}\nabla_{\mathbf{y}}\right) + \rho^{\circ}\,\boldsymbol{\delta}\mathbf{u}\cdot(\dot{\bar{\mathbf{v}}} + \eta\dot{\mathbf{v}}^1 + \cdots)\right]\mathrm{d}\Omega =$$
$$\int\limits_{\Omega}\boldsymbol{\delta}\mathbf{u}\cdot\left(\mathbf{f} + \frac{1}{\eta}\boldsymbol{\beta}\right)\mathrm{d}\Omega + \int\limits_{\Gamma_{\mathrm{t}}}\boldsymbol{\delta}\mathbf{u}\cdot\mathbf{t}_n\mathrm{d}\Gamma_{\mathrm{t}} + \int\limits_{\mathrm{S}}\boldsymbol{\delta}\mathbf{u}\cdot\mathbf{p}\mathrm{dS}, \tag{S8}$$

where $\mathbf{f} = \nabla_{\mathbf{x}}\boldsymbol{\Phi}_f + \nabla_{\mathbf{x}}\times\boldsymbol{\Psi}_f$ and $\boldsymbol{\beta} = \nabla_{\mathbf{y}}\boldsymbol{\Phi}_f + \nabla_{\mathbf{y}}\times\boldsymbol{\Psi}_f$ are macro- and micro-body forces. Assuming that all functions are smooth enough so that the limit when $\eta\to0^+$ exists for all integrals, (S8) holds if the equations with terms of the same power of η hold. The first three equations extracted from (S8) are

$$\frac{1}{\eta^2}\int\limits_{\Omega}[(\boldsymbol{\delta}\mathbf{u}\,\nabla_{\mathbf{y}}):\mathbf{C}^{\circ}:(\bar{\mathbf{u}}\,\nabla_{\mathbf{y}})]\,\mathrm{d}\Omega = 0, \tag{S9a}$$

$$\frac{1}{\eta}\int\limits_{\Omega}\left[(\boldsymbol{\delta}\mathbf{u}\,\nabla_{\mathbf{y}}):\mathbf{C}^{\circ}:\left(\bar{\mathbf{u}}\,\nabla_{\mathbf{x}} + \mathbf{u}^1\,\nabla_{\mathbf{y}}\right) + (\boldsymbol{\delta}\mathbf{u}\,\nabla_{\mathbf{x}}):\mathbf{C}^{\circ}:(\bar{\mathbf{u}}\,\nabla_{\mathbf{y}})\right]\mathrm{d}\Omega = \frac{1}{\eta}\int\limits_{\Omega}\boldsymbol{\delta}\mathbf{u}\cdot\boldsymbol{\beta}\,\mathrm{d}\Omega + \frac{1}{\eta}\int\limits_{\mathrm{S}}\boldsymbol{\delta}\mathbf{u}\cdot\mathbf{p}\,\mathrm{dS}, \tag{S9b}$$

$$\int\limits_{\Omega}\left[(\boldsymbol{\delta}\mathbf{u}\,\nabla_{\mathbf{x}}):\mathbf{C}^{\circ}:\left(\bar{\mathbf{u}}\,\nabla_{\mathbf{x}} + \mathbf{u}^1\,\nabla_{\mathbf{y}}\right) + (\boldsymbol{\delta}\mathbf{u}\,\nabla_{\mathbf{y}}):\mathbf{C}:\left(\mathbf{u}^1\,\nabla_{\mathbf{x}} + \mathbf{u}^2\,\nabla_{\mathbf{y}}\right) + \rho^{\circ}\,\boldsymbol{\delta}\mathbf{u}\cdot\dot{\bar{\mathbf{v}}}\right]\mathrm{d}\Omega = \int\limits_{\Omega}\boldsymbol{\delta}\mathbf{u}\cdot\mathbf{f}\,\mathrm{d}\Omega + \int\limits_{\Gamma_{\mathrm{t}}}\boldsymbol{\delta}\mathbf{u}\cdot\mathbf{t}_n\,\mathrm{d}\Gamma_{\mathrm{t}}. \tag{S9c}$$

Using the asymptotic homogenization technique, the elasticity problem (S4) is decomposed into three problems on different levels: *Microscopic* (S9a), *Mesoscopic* (S9b), and *Macroscopic* (S9c) levels. From the microscopic level problem, the variation of macroscopic displacement field $\bar{\mathbf{u}}$ is evaluated. The mesoscopic level problem enables the computation of fluctuating displacement field $\mathbf{u}^1$ based on the macroscopic deformation terms. The macroscopic level problem produces constitutive equations, governing equations as well as boundary conditions.

S1.3 Microscopic level

In the classical AH it is shown that $\bar{\mathbf{u}}$ does not vary with respect to $\mathbf{y}$. The assumption that leads to this solution is periodicity of $\bar{\mathbf{u}}$ with respect to $\mathbf{y}$ [15]. However, the parts of deformation that have the same periodicity as microstructure are $\mathbf{u}^1$, $\mathbf{u}^2$, etc, while the variation of the macroscopic displacement field $\bar{\mathbf{u}}$ is governed by the macroscopic loads and boundary conditions and is not necessarily periodic. Assume a bar made of a material with microstructure under simple tension. It is clear that under such condition, a unit cell

inside the material wold be stretched in the direction of applied load, and its boundaries would move in opposite directions, which indeed is not a periodic deformation. In fact, limiting the macroscopic displacement to be periodic with respect microscopic coordinate $\mathbf{y}$, is in contradiction with existence of any kind of macroscopic strain. Putting aside such a unphysical assumption and solving the microscopic level problem opens new doors to the effective behavior of materials with microstructure. Multiplying (S9a) by η^2 and taking the limit $\eta \to 0^+$ and using the property (S7a) yields

$$\int_{\Omega}\int_{¥} [(\boldsymbol{\delta}\mathbf{u}\,\nabla_{\mathbf{y}}):\mathbf{C}:(\bar{\mathbf{u}}\,\nabla_{\mathbf{y}})]\,\mathrm{d}¥\,\mathrm{d}\Omega = 0. \tag{S10}$$

Careful examination of the classical AH reveals that only a trivial solution $\bar{\mathbf{u}}\boldsymbol{\nabla}_{\mathbf{y}} = 0$ is considered for the microscopic level problem (S10) resulting in constant macroscopic fields over the RVE. The assumption that leads to this solution is the periodicity of macroscopic displacement field $\bar{\mathbf{u}}$ with respect to $\mathbf{y}$ (see page 253 of [15]), which is in contradiction with the existence of any macroscopic deformation. A simple contrary instance is the unidirectional tension of material in which the opposite boundaries of the RVE move away from each other and the displacement field is obviously not periodic. Removing such an unphysical assumption, and using the symmetry of the elasticity tensor $\mathbf{C}$, the complete solution of the microscopic problem is found as

$$\bar{\mathbf{u}}\boldsymbol{\nabla}_{\mathbf{y}} = \hat{\boldsymbol{\theta}}\times\mathbf{I} \Rightarrow \bar{\mathbf{u}}\,[\mathbf{x},\mathbf{y}] = \hat{\mathbf{u}}\,[\mathbf{x}] + \hat{\boldsymbol{\theta}}\,[\mathbf{x}]\times\mathbf{y}, \tag{S11}$$

where $\hat{\mathbf{u}}$ and $\hat{\boldsymbol{\theta}}$ are the translation vector and the rotation axial-vector describing the macroscopic kinematics of an RVE whose origin of local coordinate is located at the coordinate $\mathbf{x}$. Applying Whitaker averaging theorem [12] for continuous interfaces, $\langle\boldsymbol{\nabla}_{\mathbf{y}}\times\mathbf{u}\rangle_{\mathrm{Y}} = \boldsymbol{\nabla}_{\mathbf{x}}\times\langle\mathbf{u}\rangle_{\mathrm{Y}}$, and using (S11) one can write

$$\begin{aligned}\hat{\mathbf{u}}\,(\mathbf{x}) &= \langle\mathbf{u}\,(\mathbf{x},\mathbf{y})\rangle_{\mathrm{Y}}\\ \hat{\boldsymbol{\theta}}\,(\mathbf{x}) &= {}^1\!/_2\boldsymbol{\nabla}_{\mathbf{x}}\times\hat{\mathbf{u}}\,(\mathbf{x})\,,\end{aligned} \tag{S12}$$

provided that the local coordinate system is placed at the geometrical center of each RVE i.e., $\langle y\rangle_{\mathrm{Y}} = 0$. One must note that (S11) is the most general solution of (S10) and macroscopic displacement fields with higher powers of $\mathbf{y}$ do not satisfy this boundary value problem. Considering the asymptotic expansion (S5) for displacement field and replacing the macroscopic part $\bar{\mathbf{u}}$ from (S11) and the rest of the series with a microscopic displacement field $\mathbf{u}^*\,[\mathbf{x}+\mathbf{y}] = \eta\mathbf{u}^1[\mathbf{x},\mathbf{y}] + \eta^2\mathbf{u}^2[\mathbf{x},\mathbf{y}] + \cdots$ results in

$$\mathbf{u}\,[\mathbf{x}+\mathbf{y}] = \hat{\boldsymbol{\theta}}\,[\mathbf{x}]\times\mathbf{y} + \hat{\mathbf{u}}\,[\mathbf{x}] + \mathbf{u}^*\,[\mathbf{x},\mathbf{y}]\,. \tag{S13}$$

Inserting (S13) into (S3b) and (S3d), implementing the chain rule (S6), and taking the limit $\eta \to 0^+$, the total strain and rotation fields, respectively, take the following forms

$$\lim_{\eta\to0^+} \boldsymbol{\epsilon} = \bar{\boldsymbol{\epsilon}} + \boldsymbol{\epsilon}^*, \tag{S14a}$$

$$\lim_{\eta\to0^+} \boldsymbol{\theta} = \bar{\boldsymbol{\theta}} + \boldsymbol{\theta}^*. \tag{S14b}$$

where the parameters with zero and asterisk superscript denote the macroscopic and microscopic parts of the fields, respectively, and are defined below

$$\bar{\boldsymbol{\epsilon}} = \mathbb{S}:\bar{\mathbf{u}}\,\boldsymbol{\nabla}_x = \hat{\boldsymbol{\epsilon}} - \mathbb{S}:(\mathbf{y}\times\hat{\boldsymbol{\kappa}})\,, \tag{S15a}$$

$$\bar{\boldsymbol{\theta}} = \frac{1}{2}\boldsymbol{\nabla}_x\times\bar{\mathbf{u}} = \hat{\boldsymbol{\theta}} + \frac{1}{2}\hat{\boldsymbol{\kappa}}\cdot\mathbf{y}, \tag{S15b}$$

$$\boldsymbol{\epsilon}^* = \mathbb{S}:\mathbf{u}^1\,\boldsymbol{\nabla}_y, \tag{S15c}$$

$$\boldsymbol{\theta}^* = \frac{1}{2}\boldsymbol{\nabla}_y\times\mathbf{u}^1, \tag{S15d}$$

in which $\hat{\boldsymbol{\epsilon}} = \mathbb{S}:\hat{\mathbf{u}}\,\boldsymbol{\nabla}_x$ and $\hat{\boldsymbol{\kappa}} = \hat{\boldsymbol{\theta}}\,\boldsymbol{\nabla}_x$ are the second order translational strain tensor and curvature axial tensor , respectively. Although microscopic coordinate $\mathbf{y}$ is appeared in the definition of macroscopic rotation axial-vector (S15b), its volumetric average is equal to $\hat{\boldsymbol{\theta}}$ since the local coordinate system is placed at the geometrical center of each RVE i.e., $\langle y\rangle_{\mathrm{Y}} = 0$.

S1.4 Mesoscopic level

The outcome of the microscopic level problem was recognition of a macroscopic displacement field which varies linearly in microscopic scale. Additionally, we showed that the measures of macroscopic deformation consists of a symmetric strain tensor ($\hat{\boldsymbol{\epsilon}}$) and a curvature pseudo-tensor ($\hat{\boldsymbol{\kappa}}$). In this subsection, considering the mesoscopic level problem ((S9b)), often referred to as cell problem in the literature, we determine the perturbing fields in terms of macroscopic fields. Multiplying (S9b) by η, taking the limit $\eta \to 0^+$, and implementing the properties introduced in Equations (S7), yields

$$\int_{\Omega} \frac{1}{|\mathrm{Y}|} \int_{¥} \boldsymbol{\delta}\mathbf{u}\, \nabla_{\mathbf{y}} : \mathbf{C}^{\circ} : (\bar{\boldsymbol{\epsilon}} + \boldsymbol{\epsilon}^{*})\, \mathrm{d}¥\, \mathrm{d}\Omega = \int_{\Omega} \frac{1}{|\mathrm{Y}|} \int_{¥} \boldsymbol{\delta}\mathbf{u} \cdot \boldsymbol{\beta}\, \mathrm{d}¥\, \mathrm{d}\Omega + \int_{\Omega} \frac{1}{|\mathrm{Y}|} \int_{\mathrm{S}} \boldsymbol{\delta}\mathbf{u} \cdot \mathbf{p}\, \mathrm{dS}\, \mathrm{d}\Omega, \tag{S16}$$

It is important to note that since local elasticity tensor ($\mathbf{C}$) possesses all the minor and major symmetries, only the symmetric part of displacement gradient (strain tensor) contributes to the cell problem. Since (S16) must satisfy any admissible displacement filed $\delta\mathbf{u}$, by assuming a periodic virtual displacement $\delta\mathbf{u} = \delta\mathbf{u}\,[\mathbf{y}]$, the cell problem becomes

$$\int_{¥} \boldsymbol{\delta}\mathbf{u}\, \nabla_{\mathbf{y}} : \mathbf{C}^{\circ} : (\bar{\boldsymbol{\epsilon}} + \boldsymbol{\epsilon}^{*})\, \mathrm{d}¥ = \int_{¥} \boldsymbol{\delta}\mathbf{u} \cdot \boldsymbol{\beta}\, \mathrm{d}¥ + \int_{\mathrm{S}} \boldsymbol{\delta}\mathbf{u} \cdot \mathbf{p}\, \mathrm{dS}, \tag{S17}$$

Cell problem given in (S17) can be solved using a similar procedure used in classical AH [8, 10]. The solution method consists of applying the components of $\hat{\boldsymbol{\epsilon}}$, $\hat{\boldsymbol{\kappa}}$, $\mathbf{p}$, and $\boldsymbol{\beta}$ independently on the RVE while periodic boundary conditions are applied on the pairs of boundaries in each direction. Then, due to linearity of the problem, the responses can be superposed to form the total solution. There are three normal and three shear components of strain $\hat{\boldsymbol{\epsilon}}$, three torsional and six bending components of curvature $\hat{\boldsymbol{\kappa}}$, and three components for each applied traction $\mathbf{p}$ and micro body force $\boldsymbol{\beta}$. Different modes of strain tensor and curvature pseudo-tensor are shown in Figs. S1 and S2. Finally, by solving the cell problem, the microscopic fields can be expressed as a function of the macroscopic fields as

$$\mathbf{u}^{1}\,[\mathbf{x},\mathbf{y}] = \mathbf{U}^{\boldsymbol{\epsilon}}\,[\mathbf{y}] : \hat{\boldsymbol{\epsilon}}\,[\mathbf{x}] + \mathbf{U}^{\boldsymbol{\kappa}}\,[\mathbf{y}] : \hat{\boldsymbol{\kappa}}\,[\mathbf{x}] + \mathbf{U}^{\mathbf{p}}\,[\mathbf{y}] \cdot \hat{\mathbf{p}}\,[\mathbf{x}] + \mathbf{U}^{\boldsymbol{\beta}}\,[\mathbf{y}] \cdot \hat{\boldsymbol{\beta}}\,[\mathbf{x}] \tag{S18a}$$

$$\boldsymbol{\epsilon}^{*}\,[\mathbf{x},\mathbf{y}] = \mathbf{E}^{\boldsymbol{\epsilon}}\,[\mathbf{y}] : \hat{\boldsymbol{\epsilon}}\,[\mathbf{x}] + \mathbf{E}^{\boldsymbol{\kappa}}\,[\mathbf{y}] : \hat{\boldsymbol{\kappa}}\,[\mathbf{x}] + \mathbf{E}^{\mathbf{p}}\,[\mathbf{y}] \cdot \hat{\mathbf{p}}\,[\mathbf{x}] + \mathbf{E}^{\boldsymbol{\beta}}\,[\mathbf{y}] \cdot \hat{\boldsymbol{\beta}}\,[\mathbf{x}] \tag{S18b}$$

$$\boldsymbol{\theta}^{*}\,[\mathbf{x},\mathbf{y}] = \boldsymbol{\Theta}^{\boldsymbol{\epsilon}}\,[\mathbf{y}] : \hat{\boldsymbol{\epsilon}}\,[\mathbf{x}] + \boldsymbol{\Theta}^{\boldsymbol{\kappa}}\,[\mathbf{y}] : \hat{\boldsymbol{\kappa}}\,[\mathbf{x}] + \boldsymbol{\Theta}^{\mathbf{p}}\,[\mathbf{y}] \cdot \hat{\mathbf{p}}\,[\mathbf{x}] + \boldsymbol{\Theta}^{\boldsymbol{\beta}}\,[\mathbf{y}] \cdot \hat{\boldsymbol{\beta}}\,[\mathbf{x}] \tag{S18c}$$

where $\hat{\mathbf{p}}$ and $\hat{\boldsymbol{\beta}}$ are the macroscopic part of their corresponding fields; $\mathbf{U}$, $\mathbf{E}$, and $\boldsymbol{\Theta}$ with different superscripts are the local microscopic tensors which relate the pointwise microscopic periodic displacement vector, strain tensor, and rotation to different macroscopic defamation measures and forces applied on the unit cell, respectively. Among them $\mathbf{U}^{\mathbf{p}}$, $\mathbf{U}^{\boldsymbol{\beta}}$, $\boldsymbol{\Theta}^{\mathbf{p}}$, and $\boldsymbol{\Theta}^{\boldsymbol{\beta}}$ are second-order arrays; $\mathbf{U}^{\boldsymbol{\epsilon}}$, $\mathbf{U}^{\boldsymbol{\kappa}}$, $\boldsymbol{\Theta}^{\boldsymbol{\epsilon}}$, $\boldsymbol{\Theta}^{\boldsymbol{\kappa}}$, $\mathbf{E}^{\mathbf{p}}$, and $\mathbf{E}^{\boldsymbol{\beta}}$ are third-order arrays; $\mathbf{E}^{\boldsymbol{\epsilon}}$ and $\mathbf{E}^{\boldsymbol{\kappa}}$ are fourth-order arrays.

S1.5 Macroscopic level

In this subsection, using the macroscopic level prbolem and the solution of the cell problem at the mesoscopic level (Equations (S18)), we construct the constitutive relations and the governing equations for the macroscopic problem. Taking the limit $\eta \to 0^{+}$ of the macroscopic level problem (S9c) and implementing the property introduced in (S7a) yields

$$\int_{\Omega} \frac{1}{|\mathrm{Y}|} \int_{¥} \Big\{ \boldsymbol{\delta}\mathbf{u}\, \nabla_{\mathbf{x}} : \mathbf{C}^{\circ} : (\bar{\boldsymbol{\epsilon}} + \boldsymbol{\epsilon}^{*}) + \boldsymbol{\delta}\mathbf{u}\, \nabla_{\mathbf{y}} : \mathbf{C}^{\circ} : \left(\mathbf{u}^{1}\, \nabla_{\mathbf{x}} + \mathbf{u}^{2}\, \nabla_{\mathbf{y}}\right) + \rho^{\circ}\, \boldsymbol{\delta}\mathbf{u} \cdot \left(\dot{\hat{\mathbf{v}}} + \dot{\hat{\boldsymbol{\omega}}} \times \mathbf{y}\right) \Big\}\, \mathrm{d}¥\, \mathrm{d}\Omega$$
$$= \int_{\Omega} \frac{1}{|\mathrm{Y}|} \int_{¥} \boldsymbol{\delta}\mathbf{u} \cdot \mathbf{f}\, \mathrm{d}¥\, \mathrm{d}\Omega + \int_{\Gamma_{\mathrm{t}}} \frac{1}{|\Gamma_{\mathrm{Y}}|} \int_{\Gamma_{\mathrm{Y}}} \boldsymbol{\delta}\mathbf{u} \cdot \mathbf{t}_{n}\, \mathrm{d}\Gamma_{\mathrm{Y}}\, \mathrm{d}\Gamma_{\mathrm{t}}. \tag{S19}$$

where $\hat{\mathbf{v}} = \dot{\hat{\mathbf{u}}}$ and $\hat{\boldsymbol{\omega}} = \dot{\hat{\boldsymbol{\theta}}}$ are the translational and angular velocities. Taking $\boldsymbol{\delta}\mathbf{u} = \boldsymbol{\delta}\mathbf{u}\,[\mathbf{y}]$ will result in a higher-order cell problem and derivation of $\mathbf{u}^{2}$. However, we showed previously in (S14) and Equations (S15) that in the limit $\eta \to 0^{+}$ the only perturbing displacement field contributing to the total strain tensor is $\mathbf{u}^{1}$. Therefore, we restrict our attention to the first-order approximation. Assuming a macroscopic admissible virtual displacement field in the form $\boldsymbol{\delta}\mathbf{u} = \boldsymbol{\delta}\hat{\mathbf{u}}\,[\mathbf{x}] + \boldsymbol{\delta}\hat{\boldsymbol{\theta}}\,[\mathbf{x}] \times \mathbf{y}$ where $\boldsymbol{\delta}\hat{\boldsymbol{\theta}}\,[\mathbf{x}] = {}^{1}\!/_{2} \boldsymbol{\nabla}_{\mathbf{x}} \times \boldsymbol{\delta}\hat{\mathbf{u}}\,(\mathbf{x})$ (S19) yields

$$\int_{\Omega} \frac{1}{|\mathrm{Y}|} \int_{¥} \{ [\boldsymbol{\delta}\hat{\boldsymbol{\epsilon}} - \mathbb{S} : (\mathbf{y} \times \boldsymbol{\delta}\hat{\boldsymbol{\kappa}})] : \mathbf{C}^{\circ} : (\bar{\boldsymbol{\epsilon}} + \boldsymbol{\epsilon}^{*}) \}\, \mathrm{d}¥\, \mathrm{d}\Omega + \int_{\Omega} \frac{1}{|\mathrm{Y}|} \int_{¥} \left(\boldsymbol{\delta}\hat{\mathbf{u}} + \boldsymbol{\delta}\hat{\boldsymbol{\theta}} \times \mathbf{y}\right) \rho^{\circ} \cdot \left(\dot{\hat{\mathbf{v}}} + \dot{\hat{\boldsymbol{\omega}}} \times \mathbf{y}\right)\, \mathrm{d}¥\, \mathrm{d}\Omega$$
$$= \int_{\Omega} \frac{1}{|\mathrm{Y}|} \int_{¥} \left(\boldsymbol{\delta}\hat{\mathbf{u}} + \boldsymbol{\delta}\hat{\boldsymbol{\theta}} \times \mathbf{y}\right) \cdot \mathbf{f}\, \mathrm{d}¥\, \mathrm{d}\Omega + \int_{\Gamma_{\mathrm{t}}} \frac{1}{|\Gamma_{\mathrm{Y}}|} \int_{\Gamma_{\mathrm{Y}}} \left(\boldsymbol{\delta}\hat{\mathbf{u}} + \boldsymbol{\delta}\hat{\boldsymbol{\theta}} \times \mathbf{y}\right) \cdot \mathbf{t}_{n}\, \mathrm{d}\Gamma_{\mathrm{Y}}\, \mathrm{d}\Gamma_{\mathrm{t}}. \tag{S20}$$

Now, inserting the solution of the cell problem (S18) into (S19) we have

$$\int_{\Omega} \frac{1}{|\mathrm{Y}|} \int_{¥} \left\{ [\boldsymbol{\delta}\hat{\boldsymbol{\epsilon}} - \mathbb{S} : (\mathbf{y} \times \boldsymbol{\delta}\hat{\boldsymbol{\kappa}})] : \mathbf{C}^{\circ} : \left[(\mathbf{E}^{\epsilon} + \mathbb{S}) : \hat{\boldsymbol{\epsilon}} + (\mathbf{E}^{\kappa} - \mathbb{S} : \mathbf{y} \times \mathbb{I}) : \hat{\boldsymbol{\kappa}} + \mathbf{E}^{\mathrm{p}} \cdot \mathbf{p} + \mathbf{E}^{\boldsymbol{\beta}} \cdot \boldsymbol{\beta} \right] \right\} \, \mathrm{d}¥ \, \mathrm{d}\Omega$$

$$+ \int_{\Omega} \frac{1}{|\mathrm{Y}|} \int_{¥} \left(\boldsymbol{\delta}\hat{\mathbf{u}} + \boldsymbol{\delta}\hat{\boldsymbol{\theta}} \times \mathbf{y} \right) \rho^{\circ} \cdot \left(\dot{\hat{\mathbf{v}}} + \dot{\hat{\boldsymbol{\omega}}} \times \mathbf{y} \right) \, \mathrm{d}¥ \, \mathrm{d}\Omega = \int_{\Omega} \frac{1}{|\mathrm{Y}|} \int_{¥} \left(\boldsymbol{\delta}\hat{\mathbf{u}} + \boldsymbol{\delta}\hat{\boldsymbol{\theta}} \times \mathbf{y} \right) \cdot \mathbf{f} \, \mathrm{d}¥ \, \mathrm{d}\Omega + \int_{\Gamma_{\mathrm{t}}} \frac{1}{|\Gamma_{\mathrm{Y}}|} \int_{\Gamma_{\mathrm{Y}}} \left(\boldsymbol{\delta}\hat{\mathbf{u}} + \boldsymbol{\delta}\hat{\boldsymbol{\theta}} \times \mathbf{y} \right) \cdot \mathbf{t}_n \, \mathrm{d}\Gamma_{\mathrm{Y}} \, \mathrm{d}\Gamma_{\mathrm{t}}. \tag{S21}$$

Integrating over the solid part of the RVE (¥), one can rearrange (S20) into the weak form of the governing equations of the macroscopic problem as presented below

$$\int_{\Omega} (\boldsymbol{\delta}\hat{\boldsymbol{\epsilon}} : \hat{\boldsymbol{\sigma}} + \boldsymbol{\delta}\hat{\boldsymbol{\kappa}} : \hat{\boldsymbol{\mu}}) \, \mathrm{d}\Omega + \int_{\Omega} \left(\boldsymbol{\delta}\hat{\mathbf{u}} \cdot \dot{\hat{\mathbf{L}}} + \boldsymbol{\delta}\hat{\boldsymbol{\theta}} \cdot \dot{\hat{\mathbf{J}}} \right) \, \mathrm{d}\Omega = \int_{\Omega} \left(\boldsymbol{\delta}\hat{\mathbf{u}} \cdot \hat{\mathbf{f}} + \boldsymbol{\delta}\hat{\boldsymbol{\theta}} \cdot \hat{\mathbf{c}} \right) \, \mathrm{d}\Omega + \int_{\Gamma_{\mathrm{t}}} \left(\boldsymbol{\delta}\hat{\mathbf{u}} \cdot \hat{\mathbf{t}}_n + \boldsymbol{\delta}\hat{\boldsymbol{\theta}} \cdot \hat{\mathbf{m}}_n \right) \, \mathrm{d}\Gamma. \tag{S22}$$

Eq. (S22) represents the principle of virtual work for an elastic body in couple-stress theory. The first integral in the left-hand side is associated with the virtual internal elastic potential energy, the second one represents the virtual kinetic energy, and the right-hand side is the virtual external work associated with the external generalized body forces and tractions. Strain tensor $\hat{\boldsymbol{\epsilon}}$ and curvature pseudo-tensors $\hat{\boldsymbol{\kappa}}$ are the generalized measures of deformation, and their work conjugates are force-stress tensor $\hat{\boldsymbol{\sigma}}$ and couple-stress pseudo-tensor $\hat{\boldsymbol{\mu}}$

$$\hat{\boldsymbol{\sigma}} = \langle \bar{\boldsymbol{\sigma}} + \boldsymbol{\sigma}^{*} \rangle_{\mathrm{Y}} \, , \tag{S23a}$$

$$\hat{\boldsymbol{\mu}} = \langle \mathbf{y} \times (\bar{\boldsymbol{\sigma}} + \boldsymbol{\sigma}^{*}) \rangle_{\mathrm{Y}} \, , \tag{S23b}$$

where $\langle \square \rangle_{\mathrm{Y}} = \frac{1}{|\mathrm{Y}|} \int_{¥} (\square) \, \mathrm{dV}$ represents the volumetric average over the unit cell; $\bar{\boldsymbol{\sigma}} = \mathbf{C}^{\circ} : \bar{\epsilon}$ and $\boldsymbol{\sigma}^{*} = \mathbf{C}^{\circ} : \boldsymbol{\epsilon}^{*}$ are the macroscopic and microscopic parts of the total stress tensor, respectively. The generalization also takes place in the Kinetic energy terms, where $\hat{\mathbf{L}}$ and $\hat{\mathbf{J}}$ represent the effective linear and angular momentum

$$\hat{\mathbf{L}} = \langle \rho^{\circ} \, \dot{\mathbf{u}} \rangle_{\mathrm{Y}} \, , \tag{S24a}$$

$$\hat{\mathbf{J}} = \langle \rho^{\circ} \, y \times \dot{\mathbf{u}} \rangle_{\mathrm{Y}} \tag{S24b}$$

Moreover, $\hat{\mathbf{f}}$ and $\hat{\mathbf{m}}$ as the effective body-force and -couple, along with $\hat{\mathbf{t}}_n$ and $\hat{\mathbf{m}}_n$ as the effective force- and couple-traction are defined below

$$\hat{\mathbf{f}} = \langle \mathbf{f} \rangle_{\mathrm{Y}} \, , \tag{S25a}$$

$$\hat{\mathbf{c}} = \langle \mathbf{y} \times \mathbf{f} \rangle_{\mathrm{Y}} \, , \tag{S25b}$$

$$\hat{\mathbf{t}}_n = \langle \mathbf{t}_n \rangle_{\Gamma_{\mathrm{Y}}} \, , \tag{S25c}$$

$$\hat{\mathbf{m}}_n = \langle \mathbf{y} \times \mathbf{t}_n \rangle_{\Gamma_{\mathrm{Y}}} \, , \tag{S25d}$$

where Γ_{Y} is the boundary of the RVE which belongs to the surface Γ_{t} and $|\Gamma_{\mathrm{Y}}|$ is its area. Also $\langle \square \rangle_{\Gamma_{\mathrm{Y}}} = \frac{1}{|\Gamma_{\mathrm{Y}}|} \int_{\Gamma_{\mathrm{Y}}} (\square) \, \mathrm{d}\Gamma_{\mathrm{Y}}$ represents the areal average over boundary of the RVE. The definition of the generalized boundary traction reveals that the AAH along with couple-stress theory are able to take constant and linearly varying applied traction on the cell boundaries into account, in contrast to AH along with Cauchy elasticity theory, in that only constant traction is conceivable. The generalized constitutive relations can be extracted from (S20) in the following form

$$\hat{\boldsymbol{\sigma}} = \mathbf{C} : \hat{\boldsymbol{\epsilon}} + \mathbf{B} : \hat{\boldsymbol{\kappa}} + \hat{\boldsymbol{\sigma}}^{\mathrm{p}} + \hat{\boldsymbol{\sigma}}^{\boldsymbol{\beta}}, \tag{S26a}$$

$$\hat{\boldsymbol{\mu}} = \mathbf{A} : \hat{\boldsymbol{\epsilon}} + \mathbf{D} : \hat{\boldsymbol{\kappa}} + \hat{\boldsymbol{\mu}}^{\mathrm{p}} + \hat{\boldsymbol{\mu}}^{\boldsymbol{\beta}}, \tag{S26b}$$

$$\hat{\mathbf{L}} = \rho \hat{\mathbf{v}} + \boldsymbol{\varrho} \times \hat{\boldsymbol{\omega}}, \tag{S26c}$$

$$\hat{\mathbf{J}} = \hat{\mathbf{v}} \times \boldsymbol{\varrho} + \boldsymbol{\iota} . \hat{\boldsymbol{\omega}}, \tag{S26d}$$

where $\mathbf{C}$ and $\mathbf{D}$ are the fourth-order effective elasticity tensors, while $\mathbf{A}$ and $\bar{B}$ are the fourth-order effective generalized axial tensors and are presented below

$$\mathbf{C} = \langle \mathbf{C}^{\circ} : (\mathbf{E}^{\epsilon} + \mathbb{S}) \rangle_{\mathrm{Y}} \, , \tag{S27a}$$

$$\mathbf{B} = \langle \mathbf{C}^{\circ} : (\mathbf{E}^{\kappa} - \mathbb{S} : \mathbf{y} \times \mathbb{I}) \rangle_{\mathrm{Y}} \, , \tag{S27b}$$

$$\mathbf{A} = \langle \mathbf{y} \times \mathbf{C}^{\circ} : (\mathbf{E}^{\epsilon} + \mathbb{S}) \rangle_{\mathrm{Y}} \, , \tag{S27c}$$

$$\mathbf{D} = \langle \mathbf{y} \times \mathbf{C}^{\circ} : (\mathbf{E}^{\kappa} - \mathbb{S} : \mathbf{y} \times \mathbb{I}) \rangle_{\mathrm{Y}} \, ; \tag{S27d}$$

$\hat{\boldsymbol{\sigma}}^{\mathrm{p}}$ and $\hat{\boldsymbol{\sigma}}^{\boldsymbol{\beta}}$ are the residual force-stresses, and $\hat{\boldsymbol{\mu}}^{\mathrm{p}}$ and $\hat{\boldsymbol{\mu}}^{\boldsymbol{\beta}}$ are the residual couple-stresses induced due to the micro- tractions and body forces, respectively

$$\hat{\boldsymbol{\sigma}}^{\mathrm{p}} = \langle \mathbf{C}^{\circ} : \mathbf{E}^{\mathrm{p}} \cdot \hat{\mathbf{p}} \rangle_{\mathrm{Y}} \, , \tag{S28a}$$

$$\hat{\boldsymbol{\sigma}}^{\boldsymbol{\beta}} = \left\langle \mathbf{C}^{\circ} : \mathbf{E}^{\boldsymbol{\beta}} \cdot \hat{\boldsymbol{\beta}} \right\rangle_{\mathrm{Y}} \, , \tag{S28b}$$

$$\hat{\boldsymbol{\mu}}^{\mathrm{p}} = \langle \mathbf{y} \times \mathbf{C}^{\circ} : \mathbf{E}^{\mathrm{p}} \cdot \hat{\mathbf{p}} \rangle_{\mathrm{Y}} \, , \tag{S28c}$$

$$\hat{\boldsymbol{\mu}}^{\boldsymbol{\beta}} = \left\langle \mathbf{y} \times \mathbf{C}^{\circ} : \mathbf{E}^{\boldsymbol{\beta}} \cdot \hat{\boldsymbol{\beta}} \right\rangle_{\mathrm{Y}} \, ; \tag{S28d}$$

$\bar{\rho}$ is the effective density, $\bar{\varrho}$ and $\bar{\iota}$ are the pseudo-vector and pseudo-tensor corresponding to the first and the second effective moment of inertia, respectively

$$\rho = \langle \rho^{\circ} \rangle_{\mathrm{Y}} \,, \quad \text{(S29a)} \qquad \boldsymbol{\varrho} = \langle \rho^{\circ} \, \mathbf{y} \rangle_{\mathrm{Y}} \,, \quad \text{(S29b)} \qquad \boldsymbol{\iota} = \langle \rho^{\circ} \, (\mathrm{I} \times \mathbf{y}) \cdot (\mathbf{y} \times \mathrm{I}) \rangle_{\mathrm{Y}} \,. \quad \text{(S29c)}$$

Using the divergence theorem, one can construct the strong form of the governing equations from (S20) as

$$\hat{\boldsymbol{\sigma}} \cdot \boldsymbol{\nabla}_{\mathbf{x}} + \hat{\mathbf{f}} - \dot{\hat{\mathbf{L}}} + \frac{1}{2} \boldsymbol{\nabla}_{\mathbf{x}} \times \left(\hat{\boldsymbol{\mu}} \cdot \boldsymbol{\nabla}_{\mathbf{x}} + \hat{\mathbf{c}} - \dot{\hat{\mathbf{J}}} \right) = 0 \qquad \text{(S30)}$$

along with the associated Dirichlet and Neumann boundary conditions defined below

Dirichlet			Neumann		
$\hat{\mathbf{u}} = \hat{\mathbf{u}}_{\mathrm{d}}$	$\forall \mathbf{x} \in \Gamma_{\mathrm{d}},$	(S31a)	$\mathbf{n} \cdot \hat{\mathbf{S}} = \hat{\mathbf{t}}_n$	$\forall \mathbf{x} \in \Gamma_{\mathrm{t}},$	(S31c)
$\hat{\boldsymbol{\theta}} = \hat{\boldsymbol{\theta}}_{\mathrm{d}}$	$\forall \mathbf{x} \in \Gamma_{\mathrm{d}},$	(S31b)	$\mathbf{n} \cdot \hat{\boldsymbol{\mu}} = \hat{\mathbf{m}}_n$	$\forall \mathbf{x} \in \Gamma_{\mathrm{t}},$	(S31d)

where the specified displacement, rotation, force-traction and couple-traction on the boundary are determined as follows

$$\hat{\mathbf{u}}_{\mathrm{d}} = \langle \mathbf{u}_{\mathrm{d}} \rangle_{\Gamma_{\mathrm{Y}}} \,, \quad \text{(S32a)} \qquad \hat{\mathbf{t}}_n = \langle \mathbf{t}_n \rangle_{\Gamma_{\mathrm{Y}}} \,, \quad \text{(S32c)}$$

$$\hat{\boldsymbol{\theta}}_{\mathrm{d}} = \frac{1}{2} \langle \boldsymbol{\nabla}_{\mathbf{y}} \times \mathbf{u}_{\mathrm{d}} \rangle_{\Gamma_{\mathrm{Y}}} \,, \quad \text{(S32b)} \qquad \hat{\mathbf{m}}_n = \langle \mathbf{y} \times \mathbf{t}_n \rangle_{\Gamma_{\mathrm{Y}}} \,. \quad \text{(S32d)}$$

A schematic representation of this model is depicted in Main text, Fig.1 (b). Careful examination of (S30) reveals that the governing equations of the current theory is exactly the same as the ones associated with couple-stress theory (CST) derived by Mindlin and Tiersten [18] where the stress tensor is assumed asymmetric. Taking the anti-symmetric part of force-stress tensor into account all the components of force- and couple-stress tensors are presented in Main text, Fig.1 (c). Now, by rewriting the conservation laws for linear and angular momentum [18] we have

$$\hat{\mathbf{S}} \cdot \nabla_{\mathbf{x}} + \hat{\mathbf{f}} = \dot{\hat{\mathbf{L}}}, \quad \text{(S33a)} \qquad \left(\hat{\boldsymbol{\mu}} + \mathbf{x} \times \hat{\mathbf{S}} \right) \cdot \boldsymbol{\nabla}_{\mathbf{x}} + \left(\hat{\mathbf{c}} + \mathbf{x} \times \hat{\mathbf{f}} \right) = \left(\dot{\hat{\mathbf{J}}} + \mathbf{x} \times \dot{\hat{\mathbf{L}}} \right), \quad \text{(S33b)}$$

where $\mathbf{S}$ is the asymmetric stress tensor and can be decomposed into symmetric and anti-symmetric parts $\hat{\mathbf{S}} = \hat{\boldsymbol{\sigma}} - \boldsymbol{\varepsilon}.\hat{\boldsymbol{\tau}}$. By simplifying (S33) we can write

$$\hat{\boldsymbol{\sigma}} \cdot \boldsymbol{\nabla} + \hat{\boldsymbol{\tau}} \times \boldsymbol{\nabla} + \hat{\mathbf{f}} = \dot{\hat{\mathbf{L}}}, \quad \text{(S34a)} \qquad \hat{\boldsymbol{\mu}} \cdot \boldsymbol{\nabla} + \hat{\mathbf{c}} + 2\hat{\boldsymbol{\tau}} + \mathbf{x} \times \cancel{(\hat{\mathbf{S}} \cdot \boldsymbol{\nabla} + \hat{\mathbf{f}} - \dot{\hat{\mathbf{L}}})} = \dot{\hat{\mathbf{J}}}, \quad \text{(S34b)}$$

where $\hat{\boldsymbol{\tau}} = \boldsymbol{\varepsilon}{:}\hat{\mathbf{S}}$ is the stress axial-vector, and using (S34b) is determined as

$$\boldsymbol{\tau} = -(\hat{\boldsymbol{\mu}} \cdot \boldsymbol{\nabla} + \hat{\mathbf{c}} - \dot{\hat{\mathbf{J}}}), \qquad \text{(S35)}$$

and by inserting it into the conservation of linear momentum (S34a) reproduces (S30). It is important to note that, only the symmetric part of stress $\hat{\boldsymbol{\sigma}}$ contributes to the energy density function and therefore is determined through constitutive equations, while its anti-symmetric part $\hat{\boldsymbol{\tau}}$ is determined from angular momentum conservation equation. This is similar to the well-known Kirchhoff–Love plate theory and Bernoulli-Euler beam theory, where the out of plane shear forces are determined using the moment equilibrium. Interestingly, the frame-sensitive global couple-stress, body couple, and angular momentum are appeared in (S33b) and are defined below

$$\tilde{\mu} = \hat{\boldsymbol{\mu}} + \mathbf{x} \times \hat{\boldsymbol{\sigma}}, \quad \text{(S36a)} \qquad \tilde{\mathbf{c}} = \hat{\mathbf{c}} + \mathbf{x} \times \hat{\mathbf{f}}, \quad \text{(S36b)} \qquad \dot{\tilde{\mathbf{J}}} = \dot{\hat{\mathbf{J}}} + \mathbf{x} \times \dot{\hat{\mathbf{L}}}. \quad \text{(S36c)}$$

S1.6 Micro-rotation

It was shown that in CST only the symmetric part of the total deformation gradient (symmetric strain field) contributes to the internal elastic potential energy, and the anti-symmetric part produce rotation. Based on (S14b), the total rotation field $\boldsymbol{\theta}$ breaks down to two parts. The average macroscopic part of total rotation $\hat{\boldsymbol{\theta}}$ can be calculated based on the displacement field, directly. However, the microscopic part of total rotation $\boldsymbol{\theta}^{*}$ is neither contributing to energy density nor can be evaluated directly from field variables. Remarkably, the anti symmetric part of the microscopic displacement gradient produce a micro-rotation field that its average $\bar{\boldsymbol{\theta}}^{*}$ depends on the deformation measures and micro-forces as follows

$$\langle \boldsymbol{\theta}^* \rangle_{\mathrm{Y}} = \boldsymbol{\Theta}^{\boldsymbol{\epsilon}} : \hat{\boldsymbol{\epsilon}} + \boldsymbol{\Theta}^{\boldsymbol{\kappa}} : \hat{\boldsymbol{\kappa}} + \boldsymbol{\Theta}^{\mathbf{p}} \cdot \hat{\mathbf{p}} + \boldsymbol{\Theta}^{\boldsymbol{\beta}} \cdot \hat{\boldsymbol{\beta}} \tag{S37}$$

where $\boldsymbol{\Theta}^{\boldsymbol{\epsilon}}$ and $\boldsymbol{\Theta}^{\boldsymbol{\kappa}}$ are third-order axial and polar tensors, respectively, and $\boldsymbol{\Theta}^{\mathbf{p}}$ and $\boldsymbol{\Theta}^{\boldsymbol{\beta}}$ are second-order axial tensors. These parameters, similar to the effective material properties, depend on the base material and symmetry of the microstructure and can be evaluated as follows

$$\boldsymbol{\Theta}^{\boldsymbol{\epsilon}} = \langle \boldsymbol{\Theta}^{\boldsymbol{\epsilon}} [\mathbf{y}] \rangle_{\mathrm{Y}} , \tag{S38a}$$

$$\boldsymbol{\Theta}^{\mathbf{p}} = \langle \boldsymbol{\Theta}^{\mathbf{p}} [\mathbf{y}] \rangle_{\mathrm{Y}} , \tag{S38b}$$

$$\boldsymbol{\Theta}^{\boldsymbol{\kappa}} = \langle \boldsymbol{\Theta}^{\boldsymbol{\kappa}} [\mathbf{y}] \rangle_{\mathrm{Y}} , \tag{S38c}$$

$$\boldsymbol{\Theta}^{\boldsymbol{\beta}} = \left\langle \boldsymbol{\Theta}^{\boldsymbol{\beta}} [\mathbf{y}] \right\rangle_{\mathrm{Y}} . \tag{S38d}$$

S1.7 Localization

The macroscopic behavior of heterogeneous materials is governed by the interaction of their constituents at microscales. Implementing homogenization, we have simplified the problem consisting of materials with microstructures to a boundary value problem on an effective homogeneous medium. Through solving such an equivalent problem, the volume-averaged strain and curvature, as well as their energy conjugates, force- and couple-stresses, can be evaluated. However, these measures of deformation and stress are not useful for design purposes, failure criteria estimation, and material behavior evaluation, to name a few. On the other hand, localization aims at evaluating the physically relevant fields at the microscale and provides a deeper insight into the state of stress and strain. Such quantitative information can then be useful for estimation of the strength of the material through evaluation of maximum principal stresses, detection of plastic regions in ductile constituents, fracture in brittle phases, and delamination if there is an interface. We have already presented the total point-wise strain tensor and rotation pseudo-vector in Equations (S14). Accordingly, the total point-wise stress tensor also can be expressed as

$$\lim_{\eta \to 0^+} \boldsymbol{\sigma} = \bar{\boldsymbol{\sigma}} + \boldsymbol{\sigma}^*, \tag{S39}$$

where, $\bar{\boldsymbol{\sigma}} = \mathbf{C} : \bar{\boldsymbol{\epsilon}}$ is the macroscopic and $\boldsymbol{\sigma}^* = \mathbf{C} : \boldsymbol{\epsilon}^*$ is the microscopic part of total stress.

S2 Augmented asymptotic homogenization (AAH)

Obtaining the material properties, body forces, and residual force- and couple-stresses associated to each of the CST, CSPT, and CSBT follow similar procedure as follows:

- Modelling the geometry of the RVE
  - The selected RVE must be able to reproduce the whole material by tessellating it in the same directions as the periodic boundary conditions. This tessellation must be respected in three directions for the 3D space (CST), and in two and one directions for 2D space (CSPT) and 1D space (CSBT), respectively. Although the smallest RVE for the 3D crystals is a parallelepiped, only smallest cuboid version of the RVE should be used for AAH. Additionally, to satisfy the assumption $\langle y \rangle_{\mathrm{Y}} = 0$ made in Sec. S1.3, the local Cartesian axes $\mathbf{y}$ should be placed at geometric center of the RVE.
  - In this study SOLIDWORKS® is used to create the geometries of the metamaterials under consideration.
- Modelling the base material's behaviour
  - It is assumed that the mechanical behaviour of the base material is governed by Cauchy elasticity. However, there is no assumption on the symmetries and level of isotropy of the base material. However, for the numerical examples and the classification of the metamaterials we assumed that the base material is isotropic and only two elastic material properties are required to model it: Young's Modulus (E°) and Poisson's ratio (ν°).
  - Mechanical module of COMSOL Multiphysics® [4] is employed to perform FEM modeling.
- Application of appropriate periodic boundary conditions
  - Depending on the selected space (1D, 2D or 3D) a number of pairs of periodic boundary conditions must be applied. For the 3D space (CST) three pairs of periodic boundary conditions are require; this number reduces to two for the 2D space (CSPT) and one for the 1D space (CSBT). The mathematical expression for such boundary conditions may be written as: $\mathbf{u}^1_+ = \mathbf{u}^1_-$, where superscripts $+$ and $-$ indicates the surfaces (Left and Right, Bottom and Top, Back and Front) that the boundary condition is applied to .
- Suppression of rigid body motion
  - The independent variables in CST and CSPT are three components of displacement vector $\hat{\mathbf{u}}$, yet in CSBT there is an additional independent variable which is a rotation component $\hat{\theta}_1$. Consequently, for 3D- and 2D-AAH we need to add three additional equations (boundary conditions) to suppress rigid body motion. However, for 1D-AAH a forth equation (boundary conditions) is required specifically to suppress rigid body rotation around x_1.
- Application of volumetric strains
  - Similar to the classical asymptotic homogenization, the number of different load cases which should be considered for determination of the generalized elasticity tensors is equal to the number of deformation measures ($\hat{\epsilon}$, $\hat{\kappa}$). This number is equal to fifteen, nine and four for 3D space (CST), 2D space (CSPT), 1D space (CSBT).
  - We implement the unit strain/curvature scheme in which at each load case one deformation measure is assumed unit and the rest are set to zero. According to the assumed values of $\hat{\epsilon}$ and $\hat{\kappa}$ at each load case a distribution of macroscopic strain field $\bar{\epsilon}$ is determined using (S15a). This strain field has to be imposed on the geometry as volumetric strains. The components of the macroscopic strain field are presented in Table. S2. Also the shapes of a cube when unit deformation measures are applied are depicted in Figs. S1-S5. Fig. S1 and Figs. S2 present deformed shapes associated to the 3D-AAH, Fig. S3 and Figs. S4 are pertinent to the 2D-AAH, and S5 is associated to 1D-AAH.
- Application of body forces and internal tractions
  - The body forces ($\boldsymbol{\beta}$) and internal tractions ($\mathbf{p}$) must be applied as separate load cases. The result obtained from application of these load cases are later used to calculate the residual force- and couple-stresses.
- Solving the cell problem
  - The solution of the boundary value problem (S17) for each load case result in the unknown variables $\mathbf{u}^1$ and can be presented by (S18a).
  - By inserting $\mathbf{u}^1$ into (S15c) one can calculate the microscopic strain field $\boldsymbol{\epsilon}^*$.
  - By inserting $\boldsymbol{\epsilon}^*$ and $\bar{\boldsymbol{\epsilon}}$ into $\bar{\boldsymbol{\sigma}} = \mathbf{C}^{\circ} : \bar{\epsilon}$ and $\boldsymbol{\sigma}^* = \mathbf{C}^{\circ} : \boldsymbol{\epsilon}^*$, and then using (S23) the force-stresses $\hat{\boldsymbol{\sigma}}$ and couple-stresses $\hat{\boldsymbol{\mu}}$ at each load case are obtained. The components of force- and couple-stresses for different spaces are summarized in Table. S2.
- Obtaining generalized elasticity tensors and residual stresses
  - After obtaining the force-stresses $\hat{\boldsymbol{\sigma}}$ and couple-stresses $\hat{\boldsymbol{\mu}}$ at each load case related to the applied deformations, one may use (S26a) and (S26b) to obtain the Elasticity tensors $\mathbf{A}$, $\mathbf{B}$, $\mathbf{C}$, and $\mathbf{D}$.
  - The force- and couple-stresses obtained from load cases pertinent to the applied body forces and internal tractions are the residual stresses.
- Obtaining generalized inertia tensors
  - Calculation of the generalized inertia tensors is done using (S29) and it does not need any FEM modelling and it solely requires geometrical integration.

S3 Symmetries of Generalized Elasticity Tensors

One of the important concepts in Crystal Physics, Neumann's Principle [20], states that if a crystal possesses certain symmetry operations, all of its physical properties must be invariant with regard to the same symmetry operations. However, some physical properties might show more symmetry than the crystal symmetry. Therefore, not all the physical properties are equally useful for revealing true point group symmetries of crystals. An instance is the Cauchy elasticity tensor that is a centrosymmetric physical property where the imposed minor symmetries reduce the number of classes of classical elasticity tensors to nine reported by Voigt [27]. However, in the context of CST, additional physical properties are able to distinguish all the symmetry classes. The elastic bending tensor ($\mathbf{D}$) has a major symmetry and therefore describes a centrosymmetric physical property and classifies the crystals into the eleven Laue classes. Remarkably, the elastic coupling axial tensor ($\mathbf{B}$) does not have major symmetry and recognizes none-centrosymmetry, polarity and chirality and, therefore, all the thirty-two symmetry classes. Additionally, the first moment of inertia ($\boldsymbol{\varrho}$) is a first-order axial-tensor (vector) and can detects polarization, while it is not able to distinguish the chirality of crystals.

S3.1 Physical Symmetries

For a hyperplastic material in the context of CST, the elastic strain function

$$U = \frac{1}{2}\left(\boldsymbol{\sigma} : \boldsymbol{\epsilon} + \boldsymbol{\mu} : \boldsymbol{\kappa}\right), \tag{S40}$$

must be continuously differentiable. Consequently, for such a material, the following must be satisfied

$$\frac{\partial}{\partial \epsilon_{ij}}\left(\frac{\partial U}{\partial \epsilon_{kl}}\right) = \frac{\partial}{\partial \epsilon_{kl}}\left(\frac{\partial U}{\partial \epsilon_{ij}}\right) \quad \Rightarrow \quad C_{ijkl} = C_{ijkl}, \tag{S41a}$$

$$\frac{\partial}{\partial \kappa_{ij}}\left(\frac{\partial U}{\partial \kappa_{kl}}\right) = \frac{\partial}{\partial \kappa_{kl}}\left(\frac{\partial U}{\partial \kappa_{ij}}\right) \quad \Rightarrow \quad D_{ijkl} = D_{ijkl}, \tag{S41b}$$

$$\frac{\partial}{\partial \kappa_{ij}}\left(\frac{\partial U}{\partial \epsilon_{kl}}\right) = \frac{\partial}{\partial \epsilon_{kl}}\left(\frac{\partial U}{\partial \kappa_{ij}}\right) \quad \Rightarrow \quad B_{ijkl} = A_{ijkl}, \tag{S41c}$$

$$\frac{\partial}{\partial \epsilon_{ij}}\left(\frac{\partial U}{\partial \kappa_{kl}}\right) = \frac{\partial}{\partial \kappa_{kl}}\left(\frac{\partial U}{\partial \epsilon_{ij}}\right) \quad \Rightarrow \quad A_{ijkl} = B_{klij}, \tag{S41d}$$

where, for the sake of clarity, the Einstein's summation notation is adopted in (S41). The elasticity tensors $\mathbf{C}$ and $\mathbf{D}$ have major symmetry ($\mathbf{C} = \mathbf{C}^{\mathrm{T}}$) and the coupling elasticity axial tensors $\mathbf{A}$ and $\mathbf{B}$ are transpose of each other ($\mathbf{A} = \mathbf{B}^{\mathrm{T}}$). Additionally, since force-stress ($\boldsymbol{\sigma}$) and its work conjugate ($\boldsymbol{\epsilon}$) are symmetric tensors, the elasticity tensor $\mathbf{C}$ has all the minor symmetries similar to the Cauchy elasticity and $\mathbf{A}$ and $\mathbf{B}$ possess some minor symmetries ($\mathbf{A}_{ijkl} = \mathbf{A}_{ijlk}$ and $\mathbf{B}_{ijkl} = \mathbf{B}_{jikl}$). In summary, the symmetries of the elasticity matrices are

$$A_{ijkl} = A_{ijkl}, \quad A_{ijkl} = B_{ijkl}, \tag{S42a}$$

$$C_{ijkl} = C_{ijkl}, \quad C_{ijkl} = C_{ijkl}, \quad C_{ijkl} = C_{ijkl}, \tag{S42b}$$

$$B_{ijkl} = B_{ijkl}, \quad B_{ijkl} = A_{ijkl}, \tag{S42c}$$

$$D_{ijkl} = D_{ijkl}. \tag{S42d}$$

The elasticity matrices ($\mathbf{A}$, $\mathbf{B}$, $\mathbf{C}$, $\mathbf{D}$) in 3D space totally have 324 elements (each fourth-order tensor has 3^4 elements). Considering the symmetries given in (S42), the number of independent parameters reduces to 120 (21 elements for $\mathbf{C}$, 54 elements for $\mathbf{B}$ (or $\mathbf{A}$) and 45 elements for $\mathbf{D}$). Additionally, the curvature measure has a geometric constraint since the divergence of a rotational field is zero ($\boldsymbol{\nabla} \cdot \boldsymbol{\theta} = 0$), therefore the spherical part of curvature semi-pseudotensor ($\boldsymbol{\kappa}^{\mathrm{O}}$) is zero, i.e. $\boldsymbol{\kappa}^{\mathrm{O}} = \mathbf{I} : \kappa = 0$. Consequently, by writing couple stresses in terms of the derivative of strain energy density with respect to curvature, we have $\mu_{kk} = \partial U / \partial \kappa_{kk} = 0$, i.e. the spherical part of couple stress is also zero $\boldsymbol{\mu}^{\mathrm{O}} = \mathbf{I} : \boldsymbol{\mu} = 0$. Considering these additional relations, one can write

$$A_{11ij} + A_{22ij} + A_{33ij} = 0, \tag{S43a}$$

$$D_{11ij} + D_{22ij} + D_{33ij} = 0, \tag{S43b}$$

$$B_{ij11} + B_{ij22} + B_{ij33} = 0, \tag{S43c}$$

$$D_{ij11} + D_{ij22} + D_{ij33} = 0, \tag{S43d}$$

which reduces the number of the independent elastic components for the most general case of anisotropy to 105 (21 elements for $\mathbf{C}$, 48 elements for $\mathbf{B}$ (or $\mathbf{A}$), and 36 elements for $\mathbf{D}$).

S3.2 Crystallographic Symmetries

A primitive unit cell is the smallest volume of a crystalline material, which builds up the whole crystal structure by tessellation along three spatial directions. Parallelepiped is the only regular geometrical body that can fill the space completely without any gap by tessellation; therefore every imaginable crystal belongs to one of the seven crystal systems that are characterized by three lattice constants (ℓ_1 ,ℓ_2 , ℓ_3) and three angles between them (α_1, α_2, α_3) (Fig. S8-a).

Furthermore, each crystal can be categorized into thirty-two crystal classes based on its intrinsic symmetries. The symmetries of a crystal are identified by a set of symmetry operations that leave at least the central point fixed while transforming the rest of the crystal into a state indistinguishable from the initial crystal state. The symmetry operations are categorized into proper (rotations) and improper (inversion, reflection, and roto-inversion) transformations. The possible transformations maintaining the three-dimensional translational symmetry of the crystal consists of inversion (i), mirror reflection (m), n-fold rotations (n) n-fold roto-inversions ($\bar{n}$) with

$n = 1, 2, 3, 4, 6$. We should note that $\bar{4}$ involves 90° rotation followed by an inversion operator and cannot be written as a combination of other symmetry operations while $\bar{1} \equiv i$, $\bar{2} \equiv 1/m$, $\bar{3} \equiv 3.i$, $\bar{6} \equiv 3/m$ (n/m means an n-fold rotation axis with a mirror plane perpendicular to the axis). The thirty-two crystal classes and their symmetries are given in Table S1. Additionally, the graphical representation of these classes, as well as their point, axis, and planes of symmetry, are depicted in Fig. S8-b.

It is worthwhile to note that symmetry classes can be categorized based on the existence of a center of symmetry into central and non-central groups. This is important since centrosymmetric physical properties do not distinguish the existence of a center of symmetry and, therefore, will only differentiate between eleven of the thirty-two symmetry classes. This reduced assembly is called Laue class after his discovery on the diffraction of X-rays by crystals [5]. Each of the eleven Laue classes contains central and non-central crystallographic point groups that behave in the same way when investigated by centrosymmetric physical properties, like X-rays diffraction experiment. The non-central classes may be polar, chiral, both, or neither. One whose symmetry operations leave more than one common point unmoved is Polar. A chiral (often also called enantiomorphic) point group is one containing only proper rotational symmetries and therefore have right-handed and left-handed variants. This classification is also listed in Table S1 for all the thirty-two symmetry classes.

Associated with each symmetry operation exist a tensor which transforms the coordinate system ($\mathbf{x}$) with base vectors $\mathbf{e}_i$ to a new coordinate system ($\mathbf{x}'$) with base vectors $\mathbf{e}'_j$. The components of a vector in the new coordinate system ($\mathbf{v}'$) in terms of the components of that vector in the old coordinate system ($\mathbf{v}$) is written as $v'_j = Q_{ji} v_i$, where $Q_{ji} = \mathbf{e}_i \cdot \mathbf{e}'_j$ is the transformation tensor. The transformation tensor associated with n-fold rotation around a unit vector ($\mathbf{a}$), mirror reflection perpendicular to a unit vector ($\mathbf{a}$), and inversion are provided below

$$Q^r_{ij}\left(n, \mathbf{a}\right) = \left(1 - \cos\left(\frac{2\pi}{n}\right)\right) a_i a_j + \cos\left(\frac{2\pi}{n}\right) \delta_{ij} - \varepsilon_{ijk} a_k \sin\left(\frac{2\pi}{n}\right), \tag{S44}$$

$$Q^m_{ij}\left(\mathbf{a}\right) = \delta_{ij} - 2 a_i a_j, \tag{S45}$$

$$Q^i_{ij} = -\delta_{ij}, \tag{S46}$$

and the transformation tensors pertinent to other symmetry operation (i.e. $\bar{3}$, $\bar{4}$, $\bar{6}$) can be constructed using the definitions provided above.

S3.3 Transformation of material property tensors

Implementing coordinate transformation, one can transform the material property tensors written in one Cartesian coordinate system in other Cartesian coordinates that has undergone any combination of symmetry operation such as rotation, reflection and inversion. The components of the material property tensors follow the transformation rules below

$$\rho' = \rho, \tag{S47a}$$

$$\varrho'_i = Q_{im} \varrho_m, \tag{S47b}$$

$$\iota'_{ij} = Q_{im} Q_{jn} \iota_{mn}, \tag{S47c}$$

$$\Theta'^{\epsilon}_{ijk} = \left|\mathbf{Q}\right| Q_{im} Q_{jn} Q_{kp} \Theta^{\epsilon}_{mnp}, \tag{S47d}$$

$$\Theta'^{\kappa}_{ijk} = \left|\mathbf{Q}\right| Q_{im} Q_{jn} Q_{kp} \Theta^{\kappa}_{mnp} \tag{S47e}$$

$$C'_{ijkl} = Q_{im} Q_{jn} Q_{kp} Q_{lq} C_{mnpq}, \tag{S47f}$$

$$B'_{ijkl} = \left|\mathbf{Q}\right| Q_{im} Q_{jn} Q_{kp} Q_{lq} B_{mnpq}, \tag{S47g}$$

$$D'_{ijkl} = Q_{im} Q_{jn} Q_{kp} Q_{lq} D_{mnpq}. \tag{S47h}$$

S4 Voigt Notation

The presentation of the higher-order material property matrices is not effective since there are many repeated material parameters due to physical and crystallographic symmetries. Therefore, we will adopt a Voigt notation in which the second-order force-stress, strain, couple-stress, and curvature tensors are replaced by a column array, and consequently, the fourth-order elasticity tensors are presented by second-order matrices. The map between components of force stress and strain tensors and their equivalent array contains six relations $\{11 \to 1,\ 22 \to 2,\ 33 \to 3,\ 12 \to 4,\ 13 \to 5,\ 23 \to 6\}$, while nine relations are required to map all components of couple stress and curvature axial tensors $\{11 \to 1,\ 22 \to 2\ ,\ 33 \to 3,\ 12 \to 4,\ 21 \to 5,\ 13 \to 6,\ 31 \to 7,\ 23 \to 8,\ 32 \to 9\}$. Consequently, the arrays corresponding to the measures of stress and deformation, and their relations take the following form in 3D space (CST), 2D space (CSPT) and 1D space (CSBT)

$$\begin{bmatrix} \hat{\sigma}_{11} \\ \hat{\sigma}_{22} \\ \hat{\sigma}_{33} \\ \hat{\sigma}_{12} \\ \hat{\sigma}_{13} \\ \hat{\sigma}_{23} \\ \hat{\mu}_{11} \\ \hat{\mu}_{22} \\ \hat{\mu}_{33} \\ \hat{\mu}_{12} \\ \hat{\mu}_{21} \\ \hat{\mu}_{13} \\ \hat{\mu}_{31} \\ \hat{\mu}_{23} \\ \hat{\mu}_{32} \end{bmatrix} = \begin{bmatrix} C_{11} & C_{12} & C_{13} & C_{14} & C_{15} & C_{16} & B_{11} & B_{12} & B_{13} & B_{14} & B_{15} & B_{16} & B_{17} & B_{18} & B_{19} \\ & C_{22} & C_{23} & C_{24} & C_{25} & C_{26} & B_{21} & B_{22} & B_{23} & B_{24} & B_{25} & B_{26} & B_{27} & B_{28} & B_{29} \\ & & C_{33} & C_{34} & C_{35} & C_{36} & B_{31} & B_{32} & B_{33} & B_{34} & B_{35} & B_{36} & B_{37} & B_{38} & B_{39} \\ & & & C_{44} & C_{45} & C_{46} & B_{41} & B_{42} & B_{43} & B_{44} & B_{45} & B_{46} & B_{47} & B_{48} & B_{49} \\ & & & & C_{55} & C_{56} & B_{51} & B_{52} & B_{53} & B_{54} & B_{55} & B_{56} & B_{57} & B_{58} & B_{59} \\ & & & & & C_{66} & B_{61} & B_{62} & B_{63} & B_{64} & B_{65} & B_{66} & B_{67} & B_{68} & B_{69} \\ & & & & & & D_{11} & D_{12} & D_{13} & D_{14} & D_{15} & D_{16} & D_{17} & D_{18} & D_{19} \\ & & & & & & & D_{22} & D_{23} & D_{24} & D_{25} & D_{26} & D_{27} & D_{28} & D_{29} \\ & & & & & & & & D_{33} & D_{34} & D_{35} & D_{36} & D_{37} & D_{38} & D_{39} \\ & & & & & & & & & D_{44} & D_{45} & D_{46} & D_{47} & D_{48} & D_{49} \\ & & & \text{Sym.} & & & & & & & D_{55} & D_{56} & D_{57} & D_{58} & D_{59} \\ & & & & & & & & & & & D_{66} & D_{67} & D_{68} & D_{69} \\ & & & & & & & & & & & & D_{77} & D_{78} & D_{79} \\ & & & & & & & & & & & & & D_{88} & D_{89} \\ & & & & & & & & & & & & & & D_{99} \end{bmatrix} \cdot \begin{bmatrix} \hat{\epsilon}_{11} \\ \hat{\epsilon}_{22} \\ \hat{\epsilon}_{33} \\ 2\hat{\epsilon}_{12} \\ 2\hat{\epsilon}_{13} \\ 2\hat{\epsilon}_{23} \\ \hat{\kappa}_{11} \\ \hat{\kappa}_{22} \\ \hat{\kappa}_{33} \\ \hat{\kappa}_{12} \\ \hat{\kappa}_{21} \\ \hat{\kappa}_{13} \\ \hat{\kappa}_{31} \\ \hat{\kappa}_{23} \\ \hat{\kappa}_{32} \end{bmatrix} \tag{S48a}$$

$$\begin{bmatrix} \hat{\sigma}_{11} \\ \hat{\sigma}_{22} \\ \hat{\sigma}_{12} \\ \hat{\mu}_{11} \\ \hat{\mu}_{22} \\ \hat{\mu}_{12} \\ \hat{\mu}_{21} \\ \hat{\mu}_{31} \\ \hat{\mu}_{32} \end{bmatrix} = \begin{bmatrix} C_{11} & C_{12} & C_{14} & B_{11} & B_{12} & B_{14} & B_{15} & B_{17} & B_{19} \\ & C_{22} & C_{24} & B_{21} & B_{22} & B_{24} & B_{25} & B_{27} & B_{29} \\ & & C_{44} & B_{41} & B_{42} & B_{44} & B_{45} & B_{47} & B_{49} \\ & & & D_{11} & D_{12} & D_{14} & D_{15} & D_{17} & D_{19} \\ & & & & D_{22} & D_{24} & D_{25} & D_{27} & D_{29} \\ & & & & & D_{44} & D_{45} & D_{47} & D_{49} \\ & & & \text{Sym.} & & & D_{55} & D_{57} & D_{59} \\ & & & & & & & D_{77} & D_{79} \\ & & & & & & & & D_{99} \end{bmatrix} \cdot \begin{bmatrix} \hat{\epsilon}_{11} \\ \hat{\epsilon}_{22} \\ 2\hat{\epsilon}_{12} \\ \hat{\kappa}_{11} \\ \hat{\kappa}_{22} \\ \hat{\kappa}_{12} \\ \hat{\kappa}_{21} \\ \hat{\kappa}_{31} \\ \hat{\kappa}_{32} \end{bmatrix} \tag{S48b}$$

$$\begin{bmatrix} \hat{\sigma}_{11} \\ \hat{\mu}_{11} \\ \hat{\mu}_{21} \\ \hat{\mu}_{31} \end{bmatrix} = \begin{bmatrix} C_{11} & B_{11} & B_{15} & B_{17} \\ & D_{11} & D_{15} & D_{17} \\ & & D_{55} & D_{57} \\ & \text{Sym.} & & D_{77} \end{bmatrix} \cdot \begin{bmatrix} \hat{\epsilon}_{11} \\ \hat{\kappa}_{11} \\ \hat{\kappa}_{21} \\ \hat{\kappa}_{31} \end{bmatrix} \tag{S48c}$$

where, the indices used for the elasticity parameters follow the same Voigt notation introduced earlier (e.g., $C_{1212} \to C_{44}$, $D_{1121} \to D_{15}$ and $B_{2231} \to B_{17}$).

S5 Constitutive and compliance relations

S5.1 3D space - CST

One may implement the constitutive relations in classic Cauchy elasticity to derive the stress components when uniform strains are applied to a cubic body. Such operation is achievable since the strain components are independent of each other. For example, using specific boundary conditions, one can apply axial strain in one direction and ensure that all the other components are zero. Nevertheless, in couple-stress theory application of uniform curvature is not possible when all the strain components are zero since curvature components are slopes of strain variation in different directions. Additionally, when the coupling elasticity tensor $\mathbf{B}$ is nonzero, which is the case for non-centrosymmetric materials, even strains are not independent of other components of deformation measures. Therefore, in the above mention cases, without solving the boundary-value problem, the actual components of force-stress and couple-stress can not be derived.

$$\hat{\boldsymbol{\sigma}} = \mathbf{C} : \hat{\boldsymbol{\epsilon}} + \mathbf{B} : \hat{\boldsymbol{\kappa}} \tag{S49a}$$
$$\hat{\boldsymbol{\mu}} = \mathbf{A} : \hat{\boldsymbol{\epsilon}} + \mathbf{D} : \hat{\boldsymbol{\kappa}}, \tag{S49b}$$

Compliance equations in classic Cauchy elasticity have been introduced to specify the deformation measures (strain) when only one component of stress is applied, and all the other components are zero. Such relations are easily derived just by inverting the classic constitutive relations. However, in couple-stress theory, it is impossible to derive such relationships without solving the boundary-value problem due to the frame-sensitive nature of global couple-stress (S36). Let us discuss this matter using an example: assume that all the generalized stresses are zero and only μ_{11} is applied at the top of a column of microstructured material. This loading is equivalent to applying a torsional moment at the top of the same column. Obviously, the material resists this loading by developing couple-stresses along with variable shear stresses in the cross-section of the column. Therefore the resistance against torsion is composed of torsional couple-stress and moment [2] of shear stresses. Consequently, for a centrosymmetric material, only the couple-stress-curvature part, and for a non-centrosymmetric material, the whole compliance relations are overall size-dependent due to the coupling between force-stress and couple-stress. Accordingly, the constitutive and compliance relations in the context of CST are only defined locally for a unit element of material and should not be treated globally. Compliance relations for one element may be derived by inverting the constitutive equations (S26) and presented as

$$\hat{\boldsymbol{\epsilon}} = \mathbf{S} : \hat{\boldsymbol{\sigma}} + \mathbf{Q} : \hat{\boldsymbol{\mu}}, \tag{S50a}$$
$$\hat{\boldsymbol{\kappa}} = \mathbf{P} : \hat{\boldsymbol{\sigma}} + \mathbf{R} : \hat{\boldsymbol{\mu}}, \tag{S50b}$$

where, S, Q, P, and R are the generalized compliance tensors. The symmetries of the compliance tensors are the same as their pertinent stiffness tensors, e.g., $S \leftrightarrow C$, $Q \leftrightarrow B$, $P \leftrightarrow A$, and $R \leftrightarrow D$ and clearly, $P = Q^T$. We should note that the determinant of the total elasticity tensor is zero and therefore is not invertible. However, by taking the into account that curvature and couple-stress are deviatoric, compliance relations are easily constructed.

S5.1.1 Isotropic Material

Single crystal materials can be categorized into the crystal classes. However, most of the materials are polycrystalline, amorphous, or semi-crystalline and do not have a periodic microstructure. Such materials are not always isotropic and often exhibit some level of anisotropy. The symmetry of these materials can be described by Curie group symmetries, sometimes called the limiting or the continuous groups [21]. All Curie groups have a common symmetry element represented by an ∞-fold rotation axis. Required symmetry elements for the seven Curie groups, as well as their geometric representations, are presented in Fig. S9.

We refer to materials with $\infty\infty$ and $\infty\infty m$ point group symmetries as isochiral and isocentral, respectively. The constitutive laws in 3D space associated with CST in Voigt notation (S48a) for these class of materials can be presented as follows

[2] In physics, a moment is the product of a distance and physical quantity.

$$
\begin{bmatrix}
C_{11} & C_{12} & C_{12} & \cdot & \cdot & \cdot & \cdot & \cdot & \cdot & \cdot & \cdot & \cdot & \cdot & \cdot & \cdot \\
 & C_{11} & C_{12} & \cdot & \cdot & \cdot & \cdot & \cdot & \cdot & \cdot & \cdot & \cdot & \cdot & \cdot & \cdot \\
 & & C_{11} & \cdot & \cdot & \cdot & \cdot & \cdot & \cdot & \cdot & \cdot & \cdot & \cdot & \cdot & \cdot \\
 & & & C^{\dagger}_{44} & \cdot & \cdot & \cdot & \cdot & \cdot & \cdot & \cdot & \cdot & \cdot & \cdot & \cdot \\
 & & & & C^{\dagger}_{44} & \cdot & \cdot & \cdot & \cdot & \cdot & \cdot & \cdot & \cdot & \cdot & \cdot \\
 & & & & & C^{\dagger}_{44} & \cdot & \cdot & \cdot & \cdot & \cdot & \cdot & \cdot & \cdot & \cdot \\
 & & & & & & D_{11} & D^{\#}_{12} & D^{\#}_{12} & \cdot & \cdot & \cdot & \cdot & \cdot & \cdot \\
 & & & & & & & D_{11} & D^{\#}_{12} & \cdot & \cdot & \cdot & \cdot & \cdot & \cdot \\
 & & & & & & & & D_{11} & \cdot & \cdot & \cdot & \cdot & \cdot & \cdot \\
 & & & & & & & & & D_{44} & D^{\dagger}_{45} & \cdot & \cdot & \cdot & \cdot \\
 & & & & & & & & & & D_{44} & \cdot & \cdot & \cdot & \cdot \\
 & & & & & & & & & & & D_{44} & D^{\dagger}_{45} & \cdot & \cdot \\
 & & & & & & & & & & & & D_{44} & \cdot & \cdot \\
 & & & & & & & & & & & & & D_{44} & D^{\dagger}_{45} \\
 & & & & & & & & & & & & & & D_{44}
\end{bmatrix} \tag{S51a}
$$

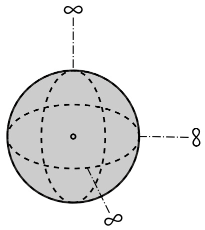

$$
\begin{bmatrix}
C_{11} & C_{12} & C_{12} & \cdot & \cdot & \cdot & B_{11} & B^{\#}_{12} & B^{\#}_{12} & \cdot & \cdot & \cdot & \cdot & \cdot & \cdot \\
 & C_{11} & C_{12} & \cdot & \cdot & \cdot & B^{\#}_{12} & B_{11} & B^{\#}_{12} & \cdot & \cdot & \cdot & \cdot & \cdot & \cdot \\
 & & C_{11} & \cdot & \cdot & \cdot & B^{\#}_{12} & B^{\#}_{12} & B_{11} & \cdot & \cdot & \cdot & \cdot & \cdot & \cdot \\
 & & & C^{\dagger}_{44} & \cdot & \cdot & \cdot & \cdot & \cdot & B^{\dagger}_{44} & B^{\dagger}_{44} & \cdot & \cdot & \cdot & \cdot \\
 & & & & C^{\dagger}_{44} & \cdot & \cdot & \cdot & \cdot & \cdot & \cdot & B^{\dagger}_{44} & B^{\dagger}_{44} & \cdot & \cdot \\
 & & & & & C^{\dagger}_{44} & \cdot & \cdot & \cdot & \cdot & \cdot & \cdot & \cdot & B^{\dagger}_{44} & B^{\dagger}_{44} \\
 & & & & & & D_{11} & D^{\#}_{12} & D^{\#}_{12} & \cdot & \cdot & \cdot & \cdot & \cdot & \cdot \\
 & & & & & & & D_{11} & D^{\#}_{12} & \cdot & \cdot & \cdot & \cdot & \cdot & \cdot \\
 & & & & & & & & D_{11} & \cdot & \cdot & \cdot & \cdot & \cdot & \cdot \\
 & & & & & & & & & D_{44} & D^{\dagger}_{45} & \cdot & \cdot & \cdot & \cdot \\
 & & & & & & & & & & D_{44} & \cdot & \cdot & \cdot & \cdot \\
 & & & & & & & & & & & D_{44} & D^{\dagger}_{45} & \cdot & \cdot \\
 & & & & & & & & & & & & D_{44} & \cdot & \cdot \\
 & & & & & & & & & & & & & D_{44} & D^{\dagger}_{45} \\
 & & & & & & & & & & & & & & D_{44}
\end{bmatrix} \tag{S51b}
$$

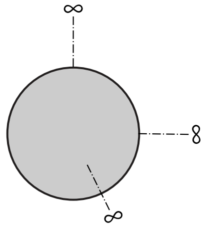

where the black color parameters are independent material properties and the gray ones are dependent. The dependent parameters are defined below

$$C^{\dagger}_{44} = \frac{C_{11} - C_{12}}{2}, \tag{S52a}$$

$$D^{\#}_{12} = -\frac{1}{2}D_{11}, \tag{S52b}$$

$$D^{\dagger}_{45} = \frac{3}{2}D_{11} - D_{44}. \tag{S52c}$$

$$B^{\#}_{12} = -\frac{1}{2}B_{11}, \tag{S52d}$$

$$B^{\dagger}_{44} = \frac{3}{4}B_{11}. \tag{S52e}$$

As another approach, the generalized elasticity tensors for isochiral materials may be constructed based on the isotropic tensors presented below:

$$C_{ijkl} = (K - \frac{2}{3}G)\delta_{ij}\delta_{kl} + G(\delta_{ik}\delta_{jl} + \delta_{il}\delta_{jk}), \tag{S53a}$$

$$B_{ijkl} = (\quad -\frac{2}{3}\beta)\delta_{ij}\delta_{kl} + \beta(\delta_{ik}\delta_{jl} + \delta_{il}\delta_{jk}), \tag{S53b}$$

$$D_{ijkl} = (\quad -\frac{2}{3}\eta)\,\delta_{ij}\delta_{kl} + \eta(\delta_{ik}\delta_{jl} + \delta_{il}\delta_{jk}) + \gamma(\delta_{ik}\delta_{jl} - \delta_{il}\delta_{jk}), \tag{S53c}$$

where K and G are the classical bulk and shear moduli; η, and γ are anticlastic (symmetric or saddle-shaped) and synclastic (anti-symmetric or dome-shaped) bending moduli; β is the coupling modulus, respectively. The relationship between the generalized lame parameters and the independent material parameters presented in (S51a) and (S51b) are

$$K = \frac{1}{3}(C_{11} + 2C_{12}), \quad (54a)$$
$$G = \frac{1}{2}(C_{11} - C_{12}) \quad (54b)$$
$$C_{11} = K + \frac{4}{3}G, \quad (55a)$$
$$C_{12} = K - \frac{2}{3}G, \quad (55b)$$
$$C^{\dagger}_{44} = G, \quad (56a)$$
$$\eta = \frac{3}{4}D_{11}, \quad (56b)$$
$$\gamma = D_{44} - \frac{3}{4}D_{11}, \quad (56c)$$
$$D_{11} = \frac{4}{3}\eta, \quad (57a)$$
$$D_{44} = \eta + \gamma, \quad (57b)$$
$$D^{\dagger}_{45} = \eta - \gamma. \quad (58a)$$
$$\beta = \frac{3}{4}B_{11}, \quad (58b)$$
$$B_{11} = \frac{4}{3}\beta, \quad (59a)$$
$$B^{\dagger}_{44} = \beta. \quad (59b)$$

We should note that the unit of generalized material properties are $\beta\,[Pa.m]$, $\eta\,[Pa.m^2]$, and $\gamma\,[Pa.m^2]$. Using (S53), the constitutive equations (S26) in the absence of residual force- and couple-stresses may be written as

$$\hat{\sigma}_{ij} = (K - \frac{2\,G}{3})\delta_{ij}\hat{\epsilon}_{kk} + G(\hat{\epsilon}_{ij} + \hat{\epsilon}_{ji}) - \frac{2}{3}\beta\delta_{ij}\hat{\kappa}_{kk} + \beta(\hat{\kappa}_{ij} + \hat{\kappa}_{ji}), \quad \text{(S60a)}$$
$$\hat{\mu}_{ij} = (\quad -\frac{2}{3}\beta)\delta_{ij}\hat{\epsilon}_{kk} + \beta\,(\hat{\epsilon}_{ij} + \hat{\epsilon}_{ji}) - \frac{2}{3}\eta\delta_{ij}\hat{\kappa}_{kk} + \eta\,(\hat{\kappa}_{ij} + \hat{\kappa}_{ji}) + \gamma(\hat{\kappa}_{ij} - \hat{\kappa}_{ji}). \quad \text{(S60b)}$$

Manipulation of (S60) results in another form of constitutive relations for isochiral materials as follows

$$\hat{\sigma}_{ij} = 3K\hat{\epsilon}_v\delta_{ij} + 2G\hat{\epsilon}_{(ij)} + 2\beta\hat{\kappa}_{(ij)}, \quad \text{(S61a)}$$
$$\hat{\mu}_{ij} = \quad 2\beta\,\hat{\epsilon}_{(ij)} + 2\eta\,\hat{\kappa}_{(ij)} + 2\gamma\hat{\kappa}_{[ij]}. \quad \text{(S61b)}$$

For isochiral materials, the generalized compliance matrices are

$$S_{ijkl} = \quad -\frac{\nu}{E}\;\delta_{ij}\delta_{kl} + \frac{1+\nu}{2\,E}(\delta_{ik}\delta_{jl} + \delta_{il}\delta_{jk}), \quad \text{(S62a)}$$
$$Q_{ijkl} = \quad -\frac{2}{3}\frac{1}{4\beta'}\delta_{ij}\delta_{kl} + \quad \frac{1}{4\beta'}(\delta_{ik}\delta_{jl} + \delta_{il}\delta_{jk}), \quad \text{(S62b)}$$
$$R_{ijkl} = -\frac{2}{3}\frac{1+\varsigma}{2\,I}\;\delta_{ij}\delta_{kl} + \frac{1+\varsigma}{2\,I}(\delta_{ik}\delta_{jl} + \delta_{il}\delta_{jk}) + \frac{1-\varsigma}{2\,I}(\delta_{ik}\delta_{jl} - \delta_{il}\delta_{jk}), \quad \text{(S62c)}$$

where primed parameters are modified due to existence of chirality. The chirality of materials can be measured through the following quantity

$$\alpha = \frac{\beta^2}{G\,\eta} \quad \text{(S63)}$$

which we name it as Lake's chirality ratio in honor of R. S. Lakes. Note that K and γ are invariant with respect to existence of chirality and the other modified lame constants may be calculated directly as

$$G' = G(1-\alpha), \quad \text{(S64a)} \qquad \eta' = \eta(1-\alpha), \quad \text{(S64b)} \qquad \beta' = \beta(1-\alpha), \quad \text{(S64c)}$$

while the modified Young's and Cosserat's moduli, Poisson's and Mindlin's ratios can be evaluated by inserting the above lame parameters in

$$E = \frac{9G'K}{3K + G'}, \quad \text{(S65a)}$$
$$\nu = \frac{3K - 2G'}{2(3K + G')}, \quad \text{(S65b)}$$
$$K = \frac{E}{3(1-2\nu)}, \quad \text{(S65c)}$$
$$G' = \frac{E}{2(1+\nu)}, \quad \text{(S65d)}$$
$$I = \frac{4\eta'\gamma}{\gamma + \eta'}, \quad \text{(S65e)}$$
$$\varsigma = \frac{\gamma - \eta'}{\gamma + \eta'}, \quad \text{(S65f)}$$
$$\gamma = \frac{I}{2(1-\varsigma)}, \quad \text{(S65g)}$$
$$\eta' = \frac{I}{2(1+\varsigma)}. \quad \text{(S65h)}$$

Replacing (S62) into (S50) results in

$$\hat{\epsilon}_{ij} = -\frac{\nu}{E}\ \hat{\sigma}_{kk}\delta_{ij} + \frac{1+\nu}{2\,E}\ (\hat{\sigma}_{ij}+\hat{\sigma}_{ji}) - \frac{2}{3}\frac{1}{4\,\beta'}\ \hat{\mu}_{kk}\delta_{ij} + \frac{1}{4\,\beta'}\ \ (\hat{\mu}_{ij}+\hat{\mu}_{ji}), \tag{S66a}$$

$$\hat{\kappa}_{ij} = -\frac{2}{3}\frac{1}{4\,\beta'}\hat{\sigma}_{kk}\delta_{ij} + \frac{1}{4\,\beta'}(\hat{\sigma}_{ij}+\hat{\sigma}_{ji}) - \frac{2}{3}\frac{1+\varsigma}{2\,I}\hat{\mu}_{kk}\delta_{ij} + \frac{1+\varsigma}{2\,I}(\hat{\mu}_{ij}+\hat{\mu}_{ji}) + \frac{1-\varsigma}{2\,I}(\hat{\mu}_{ij}-\hat{\mu}_{ji}). \tag{S66b}$$

Further manipulation of (S66) results in

$$\hat{\epsilon}_{ij} = \frac{1}{3\,K}\hat{\sigma}_v\delta_{ij} + \frac{1}{2\,G'}\hat{\sigma}_{(ij)} + \frac{1}{2\,\beta'}\hat{\mu}_{(ij)}, \tag{S67a}$$

$$\hat{\kappa}_{ij} = \frac{1}{2\,\beta'}\hat{\sigma}_{(ij)} + \frac{1}{2\,\eta'}\hat{\mu}_{(ij)} + \frac{1}{2\,\gamma}\hat{\mu}_{[ij]}. \tag{S67b}$$

By setting the terms containing β to zero and removing the effect of chirality in (S66) and (S67) the compliance relations for isocentral materials can be obtained.

S5.1.2 Cubic crystals

Artificial materials like composites and cellular metamaterials do not show isotropic behavior, generally. The maximum symmetry possible in such materials belongs to crystal classes No.28 ($m\bar{3}m$) and No.29 (432). These two crystal classes have the same rotational symmetries, yet these symmetry operations are all improper for No.28, while for No.29, they are all proper. The existence of improper symmetry operations makes crystal class No.28 centrosymmetric, while its absence makes crystal class No.29 chiral. The constitutive laws in 3D space associated with CST in Voigt notation (S48a) for materials belonging to the crystal class No.28 and NO.29 can be presented as follows

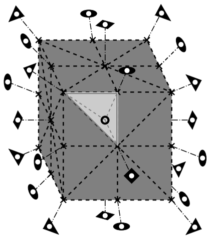

$$\begin{bmatrix}
C_{11} & C_{12} & C_{12} & \cdot & \cdot & \cdot & \cdot & \cdot & \cdot & \cdot & \cdot & \cdot & \cdot & \cdot & \cdot \\
 & C_{11} & C_{12} & \cdot & \cdot & \cdot & \cdot & \cdot & \cdot & \cdot & \cdot & \cdot & \cdot & \cdot & \cdot \\
 & & C_{11} & \cdot & \cdot & \cdot & \cdot & \cdot & \cdot & \cdot & \cdot & \cdot & \cdot & \cdot & \cdot \\
 & & & C_{44} & \cdot & \cdot & \cdot & \cdot & \cdot & \cdot & \cdot & \cdot & \cdot & \cdot & \cdot \\
 & & & & C_{44} & \cdot & \cdot & \cdot & \cdot & \cdot & \cdot & \cdot & \cdot & \cdot & \cdot \\
 & & & & & C_{44} & \cdot & \cdot & \cdot & \cdot & \cdot & \cdot & \cdot & \cdot & \cdot \\
 & & & & & & D_{11} & D^{\#}_{12} & D^{\#}_{12} & \cdot & \cdot & \cdot & \cdot & \cdot & \cdot \\
 & & & & & & & D_{11} & D^{\#}_{12} & \cdot & \cdot & \cdot & \cdot & \cdot & \cdot \\
 & & & & & & & & D_{11} & \cdot & \cdot & \cdot & \cdot & \cdot & \cdot \\
 & & & & & & & & & D_{44} & D_{45} & \cdot & \cdot & \cdot & \cdot \\
 & & & & & & & & & & D_{44} & \cdot & \cdot & \cdot & \cdot \\
 & & & & & & & & & & & D_{44} & D_{45} & \cdot & \cdot \\
 & & & & & & & & & & & & D_{44} & \cdot & \cdot \\
 & & & & & & & & & & & & & D_{44} & D_{45} \\
 & & & & & & & & & & & & & & D_{44}
\end{bmatrix} \tag{S68a}$$

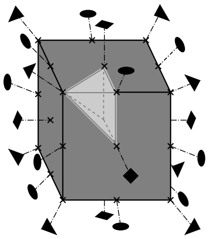

$$\begin{bmatrix}
C_{11} & C_{12} & C_{12} & \cdot & \cdot & \cdot & B_{11} & B^{\#}_{12} & B^{\#}_{12} & \cdot & \cdot & \cdot & \cdot & \cdot & \cdot \\
 & C_{11} & C_{12} & \cdot & \cdot & \cdot & B^{\#}_{12} & B_{11} & B^{\#}_{12} & \cdot & \cdot & \cdot & \cdot & \cdot & \cdot \\
 & & C_{11} & \cdot & \cdot & \cdot & B^{\#}_{12} & B^{\#}_{12} & B_{11} & \cdot & \cdot & \cdot & \cdot & \cdot & \cdot \\
 & & & C_{44} & \cdot & \cdot & \cdot & \cdot & \cdot & B_{44} & B_{44} & \cdot & \cdot & \cdot & \cdot \\
 & & & & C_{44} & \cdot & \cdot & \cdot & \cdot & \cdot & \cdot & B_{44} & B_{44} & \cdot & \cdot \\
 & & & & & C_{44} & \cdot & \cdot & \cdot & \cdot & \cdot & \cdot & \cdot & B_{44} & B_{44} \\
 & & & & & & D_{11} & D^{\#}_{12} & D^{\#}_{12} & \cdot & \cdot & \cdot & \cdot & \cdot & \cdot \\
 & & & & & & & D_{11} & D^{\#}_{12} & \cdot & \cdot & \cdot & \cdot & \cdot & \cdot \\
 & & & & & & & & D_{11} & \cdot & \cdot & \cdot & \cdot & \cdot & \cdot \\
 & & & & & & & & & D_{44} & D_{45} & \cdot & \cdot & \cdot & \cdot \\
 & & & & & & & & & & D_{44} & \cdot & \cdot & \cdot & \cdot \\
 & & & & & & & & & & & D_{44} & D_{45} & \cdot & \cdot \\
 & & & & & & & & & & & & D_{44} & \cdot & \cdot \\
 & & & & & & & & & & & & & D_{44} & D_{45} \\
 & & & & & & & & & & & & & & D_{44}
\end{bmatrix} \tag{S68b}$$

where the black color parameters are independent material properties and the gray ones are dependent. The dependent parameters are related to the independent ones as follows

$$B^{\#}_{12} = -\frac{1}{2}B_{11}, \tag{S69a}$$

$$D^{\#}_{12} = -\frac{1}{2}D_{11},. \tag{S69b}$$

In contrast to isocentral materials, for cubic crystals No.28 the shear (G_s) and bending moduli (η_b) are independent of axial (G_a)

and torsional (η_t), respectively. The same holds for isochiral materials in comparison to crystal class No.29, while additionally the axial-torsional ($\beta_{\text{a-t}}$) and shear-bending ($\beta_{\text{s-b}}$) coupling moduli are also independent. Therefore, by separating the diagonal and off-diagonal components, we can present the constitutive equations (S26) in the absence of residual force- and couple-stresses as

$$\text{diagonal } i{=}j \quad \left|\begin{aligned}\hat{\sigma}_{ij} &= 3K\hat{\epsilon}_v\delta_{ij} + 2G_{\text{a}}\hat{\epsilon}_{(ij)} + 2\beta_{\text{a-t}}\hat{\kappa}_{(ij)}\\ \hat{\mu}_{ij} &= 2\beta_{\text{a-t}}\hat{\epsilon}_{(ij)} + 2\eta_{\text{t}}\hat{\kappa}_{(ij)}\end{aligned}\right. , \tag{S70a}$$

$$\text{off-diagonal } i{\neq}j \quad \left|\begin{aligned}\hat{\sigma}_{ij} &= 2G_{\text{s}}\hat{\epsilon}_{(ij)} + 2\beta_{\text{s-b}}\hat{\kappa}_{(ij)}\\ \hat{\mu}_{ij} &= 2\beta_{\text{s-b}}\hat{\epsilon}_{(ij)} + 2\eta_{\text{b}}\hat{\kappa}_{(ij)} + 2\gamma\hat{\kappa}_{[ij]}\end{aligned}\right. \tag{S70b}$$

The above equation can be used to obtain constitutive relations associated to cubic crystal No.28 by setting $\beta_{\text{a-t}}$ and $\beta_{\text{s-b}}$ to zero. The relationship between the generalized lame parameters and the independent material parameters presented in (S68) are

$$K = \frac{1}{3}(C_{11} + 2C_{12}), \tag{71a}$$
$$G_{\text{a}} = \frac{1}{2}(C_{11} - C_{12}), \tag{71b}$$
$$G_{\text{s}} = C_{44} \quad , \tag{71c}$$
$$C_{11} = K + \frac{4}{3}G_a, \tag{72a}$$
$$C_{12} = K - \frac{2}{3}G_a, \tag{72b}$$
$$C_{44} = G_{\text{s}}, \tag{73a}$$
$$\eta_{\text{t}} = \frac{3}{4}D_{11}, \tag{73b}$$
$$\eta_{\text{b}} = \frac{1}{2}(D_{44} + D_{45}), \tag{73c}$$
$$\gamma = \frac{1}{2}(D_{44} - D_{45}), \tag{73d}$$
$$D_{11} = \frac{4}{3}\eta_{\text{t}}, \tag{74a}$$
$$D_{44} = \eta_{\text{b}} + \gamma, \tag{74b}$$
$$D_{45} = \eta_{\text{b}} - \gamma. \tag{75a}$$
$$\beta_{\text{a-t}} = \frac{3}{4}B_{11}, \tag{75b}$$
$$\beta_{\text{s-b}} = B_{44}, \tag{75c}$$
$$B_{11} = \frac{4}{3}\beta_{\text{a-t}}, \tag{76a}$$
$$B_{44} = \beta_{\text{s-b}}. \tag{76b}$$

The generalized compliance relation associated to cubic crystals No.29 are

$$\text{diagonal } i{=}j \quad \left|\begin{aligned}\hat{\epsilon}_{ij} &= -\frac{\nu}{E}\hat{\sigma}_{kk}\delta_{ij} + \frac{1+\nu}{2\,E}(\hat{\sigma}_{ij}+\hat{\sigma}_{ji}) - \frac{2}{3}\frac{1}{4\,\beta'_{\text{a-t}}}\hat{\mu}_{kk}\delta_{ij} + \frac{1}{4\,\beta'_{\text{a-t}}}(\hat{\mu}_{ij}+\hat{\mu}_{ji})\\ \hat{\kappa}_{ij} &= -\frac{2}{3}\frac{1}{4\,\beta'_{\text{a-t}}}\hat{\sigma}_{kk}\delta_{ij} + \frac{1}{4\,\beta'_{\text{a-t}}}(\hat{\sigma}_{ij}+\hat{\sigma}_{ji}) - \frac{2}{3}\frac{1}{2\,\eta'_{\text{t}}}\hat{\mu}_{kk}\delta_{ij} + \frac{1}{2\,\eta'_{\text{t}}}(\hat{\mu}_{ij}+\hat{\mu}_{ji})\end{aligned}\right. , \tag{S77a}$$

$$\text{off-diagonal } i{\neq}j \quad \left|\begin{aligned}\hat{\epsilon}_{ij} &= \frac{1}{2\,G'_{\text{s}}}(\hat{\sigma}_{ij}+\hat{\sigma}_{ji}) + \frac{1}{4\,\beta'_{\text{s-b}}}(\hat{\mu}_{ij}+\hat{\mu}_{ji})\\ \hat{\kappa}_{ij} &= \frac{1}{4\,\beta'_{\text{s-b}}}(\hat{\sigma}_{ij}+\hat{\sigma}_{ji}) + \frac{1+\varsigma}{2\,I}(\hat{\mu}_{ij}+\hat{\mu}_{ji}) + \frac{1-\varsigma}{2\,I}(\hat{\mu}_{ij}-\hat{\mu}_{ji})\end{aligned}\right. \tag{S77b}$$

which can also be presented as follows

$$\text{diagonal } i{=}j \quad \left|\begin{aligned}\hat{\epsilon}_{ij} &= \frac{1}{3\,K}\hat{\sigma}_v\delta_{ij} + \frac{1}{2\,G'_{\text{a}}}\hat{\sigma}_{(ij)} + \frac{1}{2\,\beta'_{\text{a-t}}}\hat{\mu}_{(ij)}\\ \hat{\kappa}_{ij} &= \frac{1}{2\,\beta'_{\text{a-t}}}\hat{\sigma}_{(ij)} + \frac{1}{2\,\eta'_{\text{t}}}\hat{\mu}_{(ij)}\end{aligned}\right. , \tag{S78a}$$

$$\text{off-diagonal } i{\neq}j \quad \left|\begin{aligned}\hat{\epsilon}_{ij} &= \frac{1}{2\,G'_{\text{s}}}\hat{\sigma}_{(ij)} + \frac{1}{2\,\beta'_{\text{s-b}}}\hat{\mu}_{(ij)}\\ \hat{\kappa}_{ij} &= \frac{1}{2\,\beta'_{\text{s-b}}}\hat{\sigma}_{(ij)} + \frac{1}{2\,\eta'_{\text{b}}}\hat{\mu}_{(ij)} + \frac{1}{2\,\gamma}\hat{\mu}_{[ij]}\end{aligned}\right. \tag{S78b}$$

The following relations exist among the compliance and constitutive parameters for cubic crystals No.29.

$$E = \frac{9G'_{\text{a}}K}{3K + G'_{\text{a}}}, \tag{S79a}$$
$$\nu = \frac{3K - 2G'_{\text{a}}}{2(3K + G'_{\text{a}})}, \tag{S79b}$$
$$K = \frac{E}{3(1-2\nu)}, \tag{S79c}$$
$$G'_{\text{a}} = \frac{E}{2(1+\nu)}, \tag{S79d}$$
$$I = \frac{4\eta'_{\text{b}}\gamma}{\gamma + \eta'_{\text{b}}}, \tag{S79e}$$
$$\varsigma = \frac{\gamma - \eta'_{\text{b}}}{\gamma + \eta'_{\text{b}}}, \tag{S79f}$$
$$\gamma = \frac{I}{2(1-\varsigma)}, \tag{S79g}$$
$$\eta'_{\text{b}} = \frac{I}{2(1+\varsigma)}. \tag{S79h}$$

where the primed material parameters are modified due to the chirality as follows

$$G'_{\mathrm{a}} = G_{\mathrm{a}}\left(1-\alpha_{\text{a-t}}\right), \quad \text{(S80a)} \qquad \eta'_{\mathrm{t}} = \eta_{\mathrm{t}}\left(1-\alpha_{\text{a-t}}\right), \quad \text{(S80c)} \qquad \beta'_{\text{a-t}} = \beta_{\text{a-t}}\left(1-\alpha_{\text{a-t}}^{-1}\right), \quad \text{(S80e)}$$

$$G'_{\mathrm{s}} = G_{\mathrm{s}}\left(1-\alpha_{\text{s-b}}\right), \quad \text{(S80b)} \qquad \eta'_{\mathrm{b}} = \eta_{\mathrm{b}}\left(1-\alpha_{\text{s-b}}\right), \quad \text{(S80d)} \qquad \beta'_{\text{s-b}} = \beta_{\text{s-b}}\left(1-\alpha_{\text{s-b}}^{-1}\right), \quad \text{(S80f)}$$

where $\alpha_{\text{a-t}}$ and $\alpha_{\text{s-b}}$ are axial-twist and shear-bending Lake's chirality ratios:

$$\alpha_{\text{a-t}} = \frac{\beta_{\text{a-t}}^2}{G_{\mathrm{a}}\,\eta_{\mathrm{t}}}, \quad \text{(S81a)} \qquad \alpha_{\text{s-b}} = \frac{\beta_{\text{s-b}}^2}{G_{\mathrm{s}}\,\eta_{\mathrm{b}}}, \quad \text{(S81b)}$$

By setting the terms containing $\beta_{\text{a-t}}$ and $\beta_{\text{s-b}}$ to zero and removing the primes in (S77) and (S78) the compliance relations for cubic crystal No.28 can be obtained.

S5.2 2D space - CSPT

All the strain and force-stress components associated with the CSPT are in-plane, and their indices vary from 1 to 2. Yet, the curvature and couple-stresses may be categorized based on their deformation mode into out-of-plane ($\kappa_{11}, \kappa_{12}, \kappa_{21}, \kappa_{22}$) and in-plane ($\kappa_{31}$ and κ_{32}) components. In the case of metaplates belonging to crystal classes under consideration in this paper (No.6 (mmm), No.14 ($4/mmm$), No.15 (422), No.17 ($4/m$), No.21 ($6/mmm$), No.23 (622), and No.24 ($6/m$)), the in-plane curvatures ($\hat{\kappa}_{31}$ and $\hat{\kappa}_{32}$) and couple-stresses ($\hat{\mu}_{31}$ and $\hat{\mu}_{32}$) are decoupled from the rest of the stress and deformation measure components. The constitutive relation among them may be presented as $\hat{\mu}_{31} = I_{31}\hat{\kappa}_{31}$ and $\hat{\mu}_{32} = I_{32}\hat{\kappa}_{32}$ where I_{31} and I_{32} are the in-plane Cosserat bending modulus. Except for the crystal class No.6, for the rest of the above-mentioned classes $I_{31} = I_{32} = I_3$.

The constitutive relations pertinent to the in-plane components of strain and force-stress and the out-of-plane deformation components of curvature and couple-stresses in the context of CSPT can be presented by

$$\hat{\sigma}_{ij} = C_{ijkl}\hat{\epsilon}_{kl} + B_{ijkl}\hat{\kappa}_{kl}, \quad \text{(S82a)}$$

$$\hat{\mu}_{ij} = A_{ijkl}\hat{\epsilon}_{kl} + D_{ijkl}\hat{\kappa}_{kl}, \quad \text{(S82b)}$$

where all the indices vary from 1 to 2 and $\mathbf{A}$, $\mathbf{B}$, $\mathbf{C}$, and $\mathbf{D}$ are 2D fourth-order tensors with 2^4 components which have similar symmetries as their 3D companion. The constitutive relation pertinent to the in-plane components of curvature and couple-stress are best presented using Voigt notation (S48b).

Note that only the in-plane couple-stress components have global counterparts ($\tilde{\mu}_{31}$ and $\tilde{\mu}_{32}$), and therefore, only the in-plane couple-stress is overall-size dependent which is decoupled from the other components of force- and couple-stresses. Consequently, except for the part relating to the in-plane couple-stress components, the rest of compliance relations for metaplates belonging to the above-mentioned classes can be constructed by inverting the constitutive relations. The same discussion applies to the compliance relations and only the relation among the in-plane force-stresses and strains and the out-of-plane couple-stresses and curvatures may be presented by

$$\hat{\epsilon}_{ij} = S_{ijkl}\hat{\sigma}_{kl} + Q_{ijkl}\hat{\mu}_{kl}, \quad \text{(S83a)}$$

$$\hat{\kappa}_{ij} = P_{ijkl}\hat{\sigma}_{kl} + R_{ijkl}\hat{\mu}_{kl}, \quad \text{(S83b)}$$

where $\mathbf{P}$, $\mathbf{Q}$, $\mathbf{R}$, and $\mathbf{S}$ are also 2D fourth-order tensors with 2^4 components. In what follows we only discuss the constitutive relations among the in-plane force-stresses and strains and the out-of-plane couple-stresses and curvatures.

S5.2.1 Hexagonal crystals

The maximum symmetry possible in metaplates belongs to crystal classes No.21 ($6/mmm$) and No.23 (622). These two crystal classes have the same rotational symmetries, yet these symmetry operations are all improper for No.21, while for No.23, they are all proper. The existence of improper symmetry operations makes crystal class No.21 centrosymmetric, while its absence makes crystal class No.23 chiral. The constitutive laws in 2D space associated with CSPT in Voigt notation (S48b) for materials belonging to the crystal class No.21 and No.23 can be presented as follows

$$\begin{bmatrix} C_{11} & C_{12} & \cdot & \cdot & \cdot & \cdot & \cdot & \cdot & \cdot \\ & C_{11} & \cdot & \cdot & \cdot & \cdot & \cdot & \cdot & \cdot \\ & & C^{\dagger}_{44} & \cdot & \cdot & \cdot & \cdot & \cdot & \cdot \\ & & & D_{11} & \bar{D}_{11} & \cdot & \cdot & \cdot & \cdot \\ & & & & D_{11} & \cdot & \cdot & \cdot & \cdot \\ & & & & & D_{44} & D^{\dagger}_{45} & \cdot & \cdot \\ & & & & & & D_{44} & \cdot & \cdot \\ & & & & & & & D_{77} & \cdot \\ & & & & & & & & D_{77} \end{bmatrix} \quad \text{(S84a)}$$

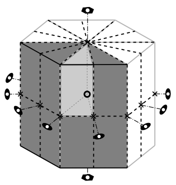

$$\begin{bmatrix} C_{11} & C_{12} & \cdot & B_{11} & \bar{B}_{11} & \cdot & \cdot & \cdot & \cdot \\ & C_{11} & \cdot & \bar{B}_{11} & B_{11} & \cdot & \cdot & \cdot & \cdot \\ & & C^{\dagger}_{44} & \cdot & \cdot & B^{\dagger}_{44} & B^{\dagger}_{44} & \cdot & \cdot \\ & & & D_{11} & \bar{D}_{11} & \cdot & \cdot & \cdot & \cdot \\ & & & & D_{11} & \cdot & \cdot & \cdot & \cdot \\ & & & & & D_{44} & D^{\dagger}_{45} & \cdot & \cdot \\ & & & & & & D_{44} & \cdot & \cdot \\ & & & & & & & D_{77} & \cdot \\ & & & & & & & & D_{77} \end{bmatrix} \quad \text{(S84b)}$$

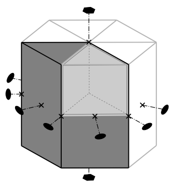

where the black color parameters are independent material properties and the gray ones are dependent. The dependent parameters are related to the independent ones as follows

$$C^{\dagger}_{44} = \frac{C_{11} - C_{12}}{2}, \quad \text{(S85a)} \qquad D^{\dagger}_{45} = 2\,D_{11} - D_{44}, \quad \text{(S85b)} \qquad B^{\dagger}_{44} = B_{11}. \quad \text{(S85c)}$$

Since the these class of materials have a 6-fold rotational symmetry, their in-plane behaviour is isotropic. Therefore, as another approach, the in-plane generalized elasticity tensors for hexachiral metaplates may be constructed based on the isotropic tensors presented below:

$$C_{ijkl} = (K - G)\delta_{ij}\delta_{kl} + G(\delta_{ik}\delta_{jl} + \delta_{il}\delta_{jk}), \quad \text{(S86a)}$$
$$B_{ijkl} = (\quad -\beta)\delta_{ij}\delta_{kl} + \beta(\delta_{ik}\delta_{jl} + \delta_{il}\delta_{jk}), \quad \text{(S86b)}$$
$$D_{ijkl} = (\quad -\eta)\,\delta_{ij}\delta_{kl} + \eta(\delta_{ik}\delta_{jl} + \delta_{il}\delta_{jk}) + \gamma(\delta_{ik}\delta_{jl} - \delta_{il}\delta_{jk}), \quad \text{(S86c)}$$

where K and G are the classical 2D bulk and shear moduli, while η, and γ are anticlastic (symmetric or saddle-shaped) and synclastic (anti-symmetric or dome-shaped) bending moduli, respectively. The relationship between the generalized lame parameters and the independent material parameters presented in (S84) are

$$K = \frac{1}{2}(C_{11} + C_{12}), \quad \text{(87a)} \qquad \eta = \frac{1}{2}(D_{44} + D^{\dagger}_{45}), \quad \text{(89b)} \qquad \beta = B_{11}, \quad \text{(91b)}$$
$$G = \frac{1}{2}(C_{11} - C_{12}), \quad \text{(87b)} \qquad \gamma = \frac{1}{2}(D_{44} - D^{\dagger}_{45}), \quad \text{(89c)}$$
$$C_{11} = K + G, \quad \text{(88a)} \qquad D_{44} = \eta + \gamma, \quad \text{(90a)} \qquad B_{11} = \beta, \quad \text{(92a)}$$
$$C_{12} = K - G, \quad \text{(88b)} \qquad D^{\dagger}_{45} = \eta - \gamma, \quad \text{(90b)} \qquad B_{44} = \beta, \quad \text{(92b)}$$
$$C^{\dagger}_{44} = G, \quad \text{(89a)} \qquad D_{11} = \eta, \quad \text{(91a)}$$

Using (S86), the constitutive equations (S82) may be written as

$$\hat{\sigma}_{ij} = (K - G)\delta_{ij}\hat{\epsilon}_{kk} + G(\hat{\epsilon}_{ij} + \hat{\epsilon}_{ji}) - \beta\delta_{ij}\hat{\kappa}_{kk} + \beta(\hat{\kappa}_{ij} + \hat{\kappa}_{ji}), \quad \text{(S93a)}$$
$$\hat{\mu}_{ij} = (\quad -\beta)\delta_{ij}\hat{\epsilon}_{kk} + \beta(\hat{\epsilon}_{ij} + \hat{\epsilon}_{ji}) - \eta\delta_{ij}\hat{\kappa}_{kk} + \eta\,(\hat{\kappa}_{ij} + \hat{\kappa}_{ji}) + \gamma(\hat{\kappa}_{ij} - \hat{\kappa}_{ji}). \quad \text{(S93b)}$$

Manipulation of (S93) results in another form of constitutive relations for isochiral materials as follows

$$\hat{\sigma}_{ij} = 2K\hat{\epsilon}_v\delta_{ij} + 2G\hat{\epsilon}_{(ij)} + 2\beta\hat{\kappa}_{(ij)}, \quad \text{(S94a)}$$
$$\hat{\mu}_{ij} = \qquad 2\beta\,\hat{\epsilon}_{(ij)} + 2\eta\,\hat{\kappa}_{(ij)} + 2\gamma\hat{\kappa}_{[ij]}. \quad \text{(S94b)}$$

For isochiral materials, the generalized compliance matrices are

$$S_{ijkl} = \quad -\frac{\nu}{E}\;\delta_{ij}\delta_{kl} + \frac{1+\nu}{2\,E}(\delta_{ik}\delta_{jl} + \delta_{il}\delta_{jk}), \quad \text{(S95a)}$$
$$Q_{ijkl} = \quad -\frac{1}{4\beta'}\delta_{ij}\delta_{kl} + \quad \frac{1}{4\beta'}(\delta_{ik}\delta_{jl} + \delta_{il}\delta_{jk}), \quad \text{(S95b)}$$
$$R_{ijkl} = -\frac{1+\varsigma}{2\,I}\;\delta_{ij}\delta_{kl} + \frac{1+\varsigma}{2\,I}\;(\delta_{ik}\delta_{jl} + \delta_{il}\delta_{jk}) + \frac{1-\varsigma}{2\,I}(\delta_{ik}\delta_{jl} - \delta_{il}\delta_{jk}), \quad \text{(S95c)}$$

where primed parameters are modified due to existence of chirality. The chirality of materials can be measured through Lake's chirality

ratio:

$$\alpha = \frac{\beta^2}{G\,\eta} \tag{S96}$$

Note that K and γ are invariant with respect to existence of chirality and the other modified lame constants may be calculated directly as

$$G' = G(1-\alpha), \quad \text{(S97a)} \qquad \eta' = \eta(1-\alpha), \quad \text{(S97b)} \qquad \beta' = \beta(1-\alpha), \quad \text{(S97c)}$$

while the modified Young's and Cosserat's moduli, Poisson's and Mindlin's ratios can be evaluated by inserting the above lame parameters in

$$E = \frac{4G'K}{K+G'}, \quad \text{(S98a)} \qquad I = \frac{4\eta'\gamma}{\gamma+\eta'}, \quad \text{(S98e)}$$

$$\nu = \frac{K-G'}{K+G'}, \quad \text{(S98b)} \qquad \varsigma = \frac{\gamma-\eta'}{\gamma+\eta'}, \quad \text{(S98f)}$$

$$K = \frac{E}{2(1-2\nu)}, \quad \text{(S98c)} \qquad \gamma = \frac{I}{2(1-\varsigma)}, \quad \text{(S98g)}$$

$$G' = \frac{E}{2(1+\nu)}, \quad \text{(S98d)} \qquad \eta' = \frac{I}{2(1+\varsigma)}. \quad \text{(S98h)}$$

Replacing (S95) into (S83) results in

$$\hat{\epsilon}_{ij} = -\frac{\nu}{E}\hat{\sigma}_{kk}\delta_{ij} + \frac{1+\nu}{2\,E}\,(\hat{\sigma}_{ij}+\hat{\sigma}_{ji}) - \frac{1}{4\,\beta'}\,\hat{\mu}_{kk}\delta_{ij} + \frac{1}{4\,\beta'}\,(\hat{\mu}_{ij}+\hat{\mu}_{ji}), \tag{S99a}$$

$$\hat{\kappa}_{ij} = -\frac{1}{4\,\beta'}\hat{\sigma}_{kk}\delta_{ij} + \frac{1}{4\,\beta'}(\hat{\sigma}_{ij}+\hat{\sigma}_{ji}) - \frac{1+\varsigma}{2\,I}\hat{\mu}_{kk}\delta_{ij} + \frac{1+\varsigma}{2\,I}(\hat{\mu}_{ij}+\hat{\mu}_{ji}) + \frac{1-\varsigma}{2\,I}(\hat{\mu}_{ij}-\hat{\mu}_{ji}). \tag{S99b}$$

Further manipulation of (S99) results in

$$\hat{\epsilon}_{ij} = \frac{1}{2\,K}\hat{\sigma}_a\delta_{ij} + \frac{1}{2\,G'}\hat{\sigma}_{(ij)} + \frac{1}{2\,\beta'}\hat{\mu}_{(ij)}, \tag{S100a}$$

$$\hat{\kappa}_{ij} = \frac{1}{2\,\beta'}\hat{\sigma}_{(ij)} + \frac{1}{2\,\eta'}\hat{\mu}_{(ij)} + \frac{1}{2\,\gamma}\hat{\mu}_{[ij]}. \tag{S100b}$$

By setting the terms containing β to zero and removing the effect of chirality in (S99) and (S100) the compliance relations for hexacentral metaplates can be obtained.

S5.2.1.1 2D-hexachiral Another metaplate with crystal class belonging to the hexagonal crystal system that is discussed in this paper is the crystal No.24 ($6/m$) which has a mirror plane perpendicular to the 6-fold rotational symmetry axis. Existence of a center of symmetry makes this class centrosymmetric, yet it is erroneously reffed to hexachiral in the literature [1, 19, 22, 23, 26]. Here we need to distinguish the definition of chirality in 2D and 3D. A 2D chiral microstructure has a pattern that cannot be brought into congruence with its mirror image unless it is lifted from the plane [2]. A 3D chiral object, however, keeps the same handedness anyways. In most of the cases the so-called hexachiral materials have 3D unitcells extruded from a 2D pattern. Therefore, to make things right we call these class of materials extruded 2D-hexachiral metapaltes. The constitutive laws in 2D space associated with CSPT in Voigt notation (S48b) for metaplates belonging to the 2D-hexachiral crystal class No.24 can be presented as follows

$$\begin{bmatrix} C_{11} & C_{12} & \cdot & \cdot & \cdot & \cdot & \cdot & \cdot & \cdot \\ & C_{11} & \cdot & \cdot & \cdot & \cdot & \cdot & \cdot & \cdot \\ & & C_{44}^{\dagger} & \cdot & \cdot & \cdot & \cdot & \cdot & \cdot \\ & & & D_{11} & \bar{D}_{11} & \cdot & \cdot & \cdot & \cdot \\ & & & & D_{11} & \cdot & \cdot & \cdot & \cdot \\ & & & & & D_{44} & D_{45}^{\dagger} & \cdot & \cdot \\ & & & & & & D_{44} & \cdot & \cdot \\ & & & & & & & D_{77} & \cdot \\ & & & & & & & & D_{77} \end{bmatrix} \tag{S101}$$

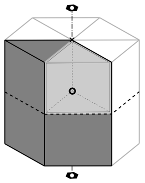

As it is observed, the 2D-hexachiral crystal class No.24 behaves similar to the hexacentral crystals.

S5.2.2 Tetragonal crystals

The next crystals in line in terms of the symmetries possible in metaplates are crystal classes No.14 ($4/mmm$) and No.15 (422). These two crystal classes also have the same rotational symmetries, yet these symmetry operations are all improper for No.14, while for No.16, they are all proper. The existence of improper symmetry operations makes crystal class No.14 centrosymmetric, while its absence makes crystal class No.15 chiral. We call the crystal classes No.14 and No.15 tetracentral and tetrachiral, respectively. The constitutive laws in 2D space associated with CSPT in Voigt notation (S48b) for materials belonging to the tetracentral and tetrachiral crystal classes can be presented as follows

$$\begin{bmatrix} C_{11} & C_{12} & \cdot & \cdot & \cdot & \cdot & \cdot & \cdot & \cdot \\ & C_{11} & \cdot & \cdot & \cdot & \cdot & \cdot & \cdot & \cdot \\ & & C_{44} & \cdot & \cdot & \cdot & \cdot & \cdot & \cdot \\ & & & D_{11} & \bar{D}_{11} & \cdot & \cdot & \cdot & \cdot \\ & & & & D_{11} & \cdot & \cdot & \cdot & \cdot \\ & & & & & D_{44} & D_{45} & \cdot & \cdot \\ & & & & & & D_{44} & \cdot & \cdot \\ & & & & & & & D_{77} & \cdot \\ & & & & & & & & D_{77} \end{bmatrix} \tag{S102a}$$

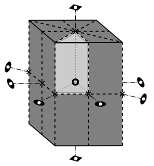

$$\begin{bmatrix} C_{11} & C_{12} & \cdot & B_{11} & \bar{B}_{11} & \cdot & \cdot & \cdot & \cdot \\ & C_{11} & \cdot & \bar{B}_{11} & B_{11} & \cdot & \cdot & \cdot & \cdot \\ & & C_{44} & \cdot & \cdot & B_{44} & B_{44} & \cdot & \cdot \\ & & & D_{11} & \bar{D}_{11} & \cdot & \cdot & \cdot & \cdot \\ & & & & D_{11} & \cdot & \cdot & \cdot & \cdot \\ & & & & & D_{44} & D_{45} & \cdot & \cdot \\ & & & & & & D_{44} & \cdot & \cdot \\ & & & & & & & D_{77} & \cdot \\ & & & & & & & & D_{77} \end{bmatrix} \tag{S102b}$$

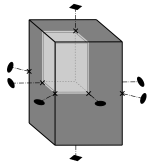

where the black colour parameters are independent material properties and the gray ones are dependent. In contrast to hexacentral mataplates, for tetracentral crystal No.14 the shear (G_s) and bending moduli (η_b) are independent of axial (G_a) and torsional (η_t), respectively. The same holds for hexachiral materials in comparison to the tertrachiral crystal class No.15, while additionally the axial-torsional ($\beta_{\text{a-t}}$) and shear-bending ($\beta_{\text{s-b}}$) coupling moduli are also independent. Therefore, by separating the diagonal and off-diagonal components, we can present the constitutive equations (S82) as

diagonal $i{=}j$

$$\begin{vmatrix} \hat{\sigma}_{ij} = 2K\hat{\epsilon}_a\delta_{ij} + 2G_{\text{a}}\hat{\epsilon}_{(ij)} + 2\beta_{\text{a-t}}\hat{\kappa}_{(ij)} \\ \hat{\mu}_{ij} = \qquad\qquad 2\beta_{\text{a-t}}\hat{\epsilon}_{(ij)} + 2\eta_{\text{t}}\hat{\kappa}_{(ij)} \end{vmatrix}, \tag{S103a}$$

off-diagonal $i{\neq}j$

$$\begin{vmatrix} \hat{\sigma}_{ij} = 2G_{\text{s}}\hat{\epsilon}_{(ij)} + 2\beta_{\text{s-b}}\hat{\kappa}_{(ij)} \\ \hat{\mu}_{ij} = 2\beta_{\text{s-b}}\hat{\epsilon}_{(ij)} + 2\eta_{\text{b}}\hat{\kappa}_{(ij)} + 2\gamma\hat{\kappa}_{[ij]} \end{vmatrix} \tag{S103b}$$

The above equation can be used to obtain constitutive relations associated to tetracentral crystal No.14 by setting $\beta_{\text{a-t}}$ and $\beta_{\text{s-b}}$ to zero. The relationship between the generalized lame parameters and the independent material parameters presented in (S102) are

$$K = \frac{1}{2}(C_{11} + C_{12}), \tag{104a}$$
$$G_{\text{a}} = \frac{1}{2}(C_{11} - C_{12}), \tag{104b}$$
$$G_{\text{s}} = C_{44} \quad , \tag{104c}$$
$$C_{11} = K + G_a, \tag{105a}$$
$$C_{12} = K - G_a, \tag{105b}$$
$$C_{44} = G_{\text{s}}, \tag{106a}$$
$$\eta_{\text{t}} = D_{11}, \tag{106b}$$
$$\eta_{\text{b}} = \frac{1}{2}(D_{44} + D_{45}), \tag{106c}$$
$$\gamma = \frac{1}{2}(D_{44} - D_{45}), \tag{106d}$$
$$D_{11} = \eta_{\text{t}}, \tag{107a}$$
$$D_{44} = \eta_{\text{b}} + \gamma, \tag{107b}$$
$$D_{45} = \eta_{\text{b}} - \gamma. \tag{108a}$$
$$\beta_{\text{a-t}} = B_{11}, \tag{108b}$$
$$\beta_{\text{s-b}} = B_{44}, \tag{108c}$$
$$B_{11} = \beta_{\text{a-t}}, \tag{109a}$$
$$B_{44} = \beta_{\text{s-b}}. \tag{109b}$$

The generalized compliance relation associated with tetrachiral crystals No.15 are

$$\text{diagonal } i{=}j \quad \left| \begin{aligned} \hat{\epsilon}_{ij} &= -\frac{\nu}{E}\hat{\sigma}_{kk}\delta_{ij} + \frac{1+\nu}{2\,E}(\hat{\sigma}_{ij}+\hat{\sigma}_{ji}) - \frac{1}{4\,\beta'_{\text{a-t}}}\hat{\mu}_{kk}\delta_{ij} + \frac{1}{4\,\beta'_{\text{a-t}}}(\hat{\mu}_{ij}+\hat{\mu}_{ji}) \\ \hat{\kappa}_{ij} &= -\frac{1}{4\,\beta'_{\text{a-t}}}\hat{\sigma}_{kk}\delta_{ij} + \frac{1}{4\,\beta'_{\text{a-t}}}(\hat{\sigma}_{ij}+\hat{\sigma}_{ji}) - \frac{1}{2\,\eta'_{\text{t}}}\hat{\mu}_{kk}\delta_{ij} + \frac{1}{2\,\eta'_{\text{t}}}(\hat{\mu}_{ij}+\hat{\mu}_{ji}) \end{aligned} \right. , \tag{S110a}$$

$$\text{off-diagonal } i{\neq}j \quad \left| \begin{aligned} \hat{\epsilon}_{ij} &= \frac{1}{2\,G'_{\text{s}}}(\hat{\sigma}_{ij}+\hat{\sigma}_{ji}) + \frac{1}{4\,\beta'_{\text{s-b}}}(\hat{\mu}_{ij}+\hat{\mu}_{ji}) \\ \hat{\kappa}_{ij} &= \frac{1}{4\,\beta'_{\text{s-b}}}(\hat{\sigma}_{ij}+\hat{\sigma}_{ji}) + \frac{1+\varsigma}{2\,I}(\hat{\mu}_{ij}+\hat{\mu}_{ji}) + \frac{1-\varsigma}{2\,I}(\hat{\mu}_{ij}-\hat{\mu}_{ji}) \end{aligned} \right. \tag{S110b}$$

which can also be presented as follows

$$\text{diagonal } i{=}j \quad \left| \begin{aligned} \hat{\epsilon}_{ij} &= \frac{1}{2\,K}\hat{\sigma}_{a}\delta_{ij} + \frac{1}{2\,G'_{\text{a}}}\hat{\sigma}_{(ij)} + \frac{1}{2\,\beta'_{\text{a-t}}}\hat{\mu}_{(ij)} \\ \hat{\kappa}_{ij} &= \qquad\qquad \frac{1}{2\,\beta'_{\text{a-t}}}\hat{\sigma}_{(ij)} + \frac{1}{2\,\eta'_{\text{t}}}\hat{\mu}_{(ij)} \end{aligned} \right. , \tag{S111a}$$

$$\text{off-diagonal } i{\neq}j \quad \left| \begin{aligned} \hat{\epsilon}_{ij} &= \frac{1}{2\,G'_{\text{s}}}\hat{\sigma}_{(ij)} + \frac{1}{2\,\beta'_{\text{s-b}}}\hat{\mu}_{(ij)} \\ \hat{\kappa}_{ij} &= \frac{1}{2\,\beta'_{\text{s-b}}}\hat{\sigma}_{(ij)} + \frac{1}{2\,\eta'_{\text{b}}}\hat{\mu}_{(ij)} + \frac{1}{2\,\gamma}\hat{\mu}_{[ij]} \end{aligned} \right. \tag{S111b}$$

The following relations exist among the compliance and constitutive parameters for cubic crystals No.29.

$$E = \frac{4G'_{\text{a}}K}{K+G'_{\text{a}}}, \tag{S112a}$$

$$\nu = \frac{K-G'_{\text{a}}}{K+G'_{\text{a}}}, \tag{S112b}$$

$$K = \frac{E}{2(1-2\nu)}, \tag{S112c}$$

$$G'_{\text{a}} = \frac{E}{2(1+\nu)}, \tag{S112d}$$

$$I = \frac{4\eta'_{\text{b}}\gamma}{\gamma+\eta'_{\text{b}}}, \tag{S112e}$$

$$\varsigma = \frac{\gamma-\eta'_{\text{b}}}{\gamma+\eta'_{\text{b}}}, \tag{S112f}$$

$$\gamma = \frac{I}{2(1-\varsigma)}, \tag{S112g}$$

$$\eta'_{\text{b}} = \frac{I}{2(1+\varsigma)}. \tag{S112h}$$

where the primed material parameters are modified due to the chirality as follows

$$G'_{\text{a}} = G_{\text{a}}\,(1-\alpha_{\text{a-t}})\,, \tag{S113a}$$

$$G'_{\text{s}} = G_{\text{s}}\,(1-\alpha_{\text{s-b}})\,, \tag{S113b}$$

$$\eta'_{\text{t}} = \eta_{\text{t}}\,(1-\alpha_{\text{a-t}})\,, \tag{S113c}$$

$$\eta'_{\text{b}} = \eta_{\text{b}}\,(1-\alpha_{\text{s-b}})\,, \tag{S113d}$$

$$\beta'_{\text{a-t}} = \beta_{\text{a-t}}\left(1-\alpha_{\text{a-t}}^{-1}\right), \tag{S113e}$$

$$\beta'_{\text{s-b}} = \beta_{\text{s-b}}\left(1-\alpha_{\text{s-b}}^{-1}\right), \tag{S113f}$$

where $\alpha_{\text{a-t}}$ and $\alpha_{\text{s-b}}$ are axial-twist and shear-bending Lake's chirality ratios:

$$\alpha_{\text{a-t}} = \frac{\beta_{\text{a-t}}^2}{G_{\text{a}}\,\eta_{\text{t}}}, \tag{S114a}$$

$$\alpha_{\text{s-b}} = \frac{\beta_{\text{s-b}}^2}{G_{\text{s}}\,\eta_{\text{b}}}, \tag{S114b}$$

By setting the terms containing $\beta_{\text{a-t}}$ and $\beta_{\text{s-b}}$ to zero and removing the primes in (S110) and (S111) the compliance relations for tetracentral crystal No.14 can be obtained.

S5.2.2.1 2D-Tetrachiral Another metaplate with crystal class belonging to the tetragonal crystal system that is discussed in this paper is the crystal No.17 ($4/m$) which has a mirror plane perpendicular to the 4-fold rotational symmetry axis. Many researchers [1, 6, 19] have categorized these calss of materials tetrachiral. However, existence of a center of symmetry is opposed to such classification ($\mathbf{B} = 0$). However, similar to the crystal class No.24 ($6/m$) that was called 2D-hexachiral, we call this class of metaplates 2D-tetrachiral. In contrast to the 2D-hexachiral metaplates that acted like a hexacentral class, materials with 2D-tetrachiral symmetries exhibit couplings axial-shear in stress-strain relations, and torsional-bending in couple-stress-curvature relations. The constitutive laws in 2D space associated with CSPT in Voigt notation (S48b) for metaplates belonging to the 2D-tetrachiral crystal class No.17 can be presented as follows

$$
\begin{bmatrix}
C_{11} & C_{12} & C_{14} & \cdot & \cdot & \cdot & \cdot & \cdot & \cdot \\
 & C_{11} & \bar{C}_{14} & \cdot & \cdot & \cdot & \cdot & \cdot & \cdot \\
 & & C_{44} & \cdot & \cdot & \cdot & \cdot & \cdot & \cdot \\
 & & & D_{11} & \bar{D}_{11} & D_{14} & D_{14} & \cdot & \cdot \\
 & & & & D_{11} & \bar{D}_{14} & \bar{D}_{14} & \cdot & \cdot \\
 & & & & & D_{44} & D_{45} & \cdot & \cdot \\
 & & & & & & D_{44} & \cdot & \cdot \\
 & & & & & & & D_{77} & \cdot \\
 & & & & & & & & D_{77}
\end{bmatrix} \tag{S115}
$$

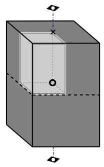

Similar to the 3D tetrachiral metaplates, the symmetric-deviatoric parts of force- and couple-stress are coupled and there is no coupling for circular part of stress ($\hat{\sigma}_{\text{a}} = 2K\hat{\epsilon}_{\text{a}}$) and anti-symmetric part of couple-stress ($\hat{\mu}_{[ij]} = 2\gamma\,\hat{\kappa}_{[ij]}$). Noting that $\hat{\sigma}_{(22)} = -\hat{\sigma}_{(11)}$, $\hat{\mu}_{(22)} = -\hat{\mu}_{(11)}$, $\hat{\epsilon}_{(22)} = -\hat{\epsilon}_{(11)}$, and $\hat{\kappa}_{(22)} = -\hat{\kappa}_{(11)}$, the rest of constitutive relations for the crystal class No.17 may be presented as

$$\hat{\sigma}_{(11)} = 2G_{\text{a}}\hat{\epsilon}_{(11)} + 2\beta_{\text{a-s}}\hat{\epsilon}_{(12)}, \tag{S116a}$$
$$\hat{\sigma}_{(12)} = 2\beta_{\text{a-s}}\hat{\epsilon}_{(11)} + 2G_{\text{s}}\hat{\epsilon}_{(12)}, \tag{S116b}$$
$$\hat{\mu}_{(11)} = 2\eta_{\text{t}}\hat{\kappa}_{(11)} + 2\beta_{\text{t-b}}\hat{\kappa}_{(12)}, \tag{S116c}$$
$$\hat{\mu}_{(12)} = 2\beta_{\text{t-b}}\hat{\kappa}_{(11)} + 2\eta_{\text{b}}\hat{\kappa}_{(12)}, \tag{S116d}$$

where $\beta_{\text{a-s}}$ and $\beta_{\text{t-b}}$ are material parameters characterizing the axial-shear and torsional-bending couplings, respectively.

$$K = \frac{1}{2}(C_{11} + C_{12}), \tag{117a}$$
$$G_{\text{a}} = \frac{1}{2}(C_{11} - C_{12}), \tag{117b}$$
$$G_{\text{s}} = C_{44} \quad , \tag{117c}$$
$$\beta_{\text{a-s}} = C_{14} \quad , \tag{117d}$$
$$C_{11} = K + G_a, \tag{118a}$$
$$C_{12} = K - G_a, \tag{118b}$$
$$C_{44} = G_{\text{s}}, \tag{118c}$$
$$C_{14} = \beta_{\text{a-s}}, \tag{119a}$$
$$\eta_{\text{t}} = D_{11}, \tag{119b}$$
$$\eta_{\text{b}} = \frac{1}{2}(D_{44} + D_{45}), \tag{119c}$$
$$\gamma = \frac{1}{2}(D_{44} - D_{45}), \tag{119d}$$
$$\beta_{\text{t-b}} = D_{14}, \tag{119e}$$
$$D_{11} = \eta_{\text{t}}, \tag{120a}$$
$$D_{44} = \eta_{\text{b}} + \gamma, \tag{120b}$$
$$D_{45} = \eta_{\text{b}} - \gamma \tag{120c}$$
$$D_{14} = \beta_{\text{a-s}}. \tag{121a}$$

Such couplings may disappear after a right-handed rotation (φ) of the axes around x_3. To determine the appropriate rotation angle, in what follows we study the coordinate transformation in the context of CSPT.

The transformed force- and couple-stress components after a right-handed rotation (φ) of the axes around x_3 are presented below

$$\hat{\sigma}'_{11} = \frac{\hat{\sigma}_{11}+\hat{\sigma}_{22}}{2} + \frac{\hat{\sigma}_{11}-\hat{\sigma}_{22}}{2}\cos(2\varphi) + \hat{\sigma}_{12}\sin(2\varphi) \tag{S122a}$$
$$\hat{\sigma}'_{22} = \frac{\hat{\sigma}_{11}+\hat{\sigma}_{22}}{2} - \frac{\hat{\sigma}_{11}-\hat{\sigma}_{22}}{2}\cos(2\varphi) - \hat{\sigma}_{12}\sin(2\varphi) \tag{S122b}$$
$$\hat{\sigma}'_{12} = -\frac{\hat{\sigma}_{11}-\hat{\sigma}_{22}}{2}\sin(2\varphi) + \hat{\sigma}_{12}\cos(2\varphi) \tag{S122c}$$
$$\hat{\mu}'_{12} = \frac{\hat{\mu}_{12}-\hat{\mu}_{21}}{2} + \frac{\hat{\mu}_{12}+\hat{\mu}_{21}}{2}\cos(2\varphi) - \hat{\mu}_{11}\sin(2\varphi) \tag{S122d}$$
$$\hat{\mu}'_{21} = \frac{\hat{\mu}_{21}-\hat{\mu}_{12}}{2} + \frac{\hat{\mu}_{12}+\hat{\mu}_{21}}{2}\cos(2\varphi) - \hat{\mu}_{11}\sin(2\varphi) \tag{S122e}$$
$$\hat{\mu}'_{11} = \frac{\hat{\mu}_{12}+\hat{\mu}_{21}}{2}\sin(2\varphi) + \hat{\mu}_{11}\cos(2\varphi) \tag{S122f}$$
$$\hat{\mu}'_{31} = \hat{\mu}_{31}\cos(\varphi) + \hat{\mu}_{32}\sin(\varphi) \tag{S122g}$$
$$\hat{\mu}'_{32} = -\hat{\mu}_{31}\sin(\varphi) + \hat{\mu}_{32}\cos(\varphi) \tag{S122h}$$

The Mohr-circles associated to the above equations are presented in Fig. S10. A After coordinate transformation, the spherical and anti-symmetric parts of a two-dimensional second order tensor remain the same, and only the symmetric-deviatoric part transforms. Noting that $\hat{\mu}_{(22)} = -\hat{\mu}_{(11)}$ and $\hat{\sigma}_{(22)} = -\hat{\sigma}_{(11)}$, one can write

$$\hat{\sigma}'_{(11)} = \hat{\sigma}_{(11)}\cos(2\varphi) + \hat{\sigma}_{(12)}\sin(2\varphi) \tag{S123a}$$
$$\hat{\sigma}'_{(12)} = -\hat{\sigma}_{(11)}\sin(2\varphi) + \hat{\sigma}_{(12)}\cos(2\varphi) \tag{S123b}$$
$$\hat{\mu}'_{(11)} = \hat{\mu}_{(11)}\cos(2\varphi) + \hat{\mu}_{(12)}\sin(2\varphi) \tag{S123c}$$
$$\hat{\mu}'_{(12)} = -\hat{\mu}_{(11)}\sin(2\varphi) + \hat{\mu}_{(12)}\cos(2\varphi) \tag{S123d}$$

The Mohr-circles associated to the above equations are presented in Fig. S11-a and Fig. S11-b. A similar relation exist between strain and curvature components and they can be easily constructed based on (S122) and (S123). The constitutive laws after a right-handed rotation (φ) of the axes around x_3 take the form

$$\hat{\sigma}'_{(11)} = 2G'_{\text{a}}\hat{\epsilon}'_{(11)} + 2\beta'_{\text{a-s}}\hat{\epsilon}'_{(12)}, \tag{S124a}$$
$$\hat{\sigma}'_{(12)} = 2\beta'_{\text{a-s}}\hat{\epsilon}'_{(11)} + 2G'_{\text{s}}\hat{\epsilon}'_{(12)}, \tag{S124b}$$
$$\hat{\mu}'_{(11)} = 2\eta'_{\text{t}}\hat{\kappa}'_{(11)} + 2\beta'_{\text{t-b}}\hat{\kappa}'_{(12)}, \tag{S124c}$$
$$\hat{\mu}'_{(12)} = 2\beta'_{\text{t-b}}\hat{\kappa}'_{(11)} + 2\eta'_{\text{b}}\hat{\kappa}'_{(12)}, \tag{S124d}$$

where

$$G'_{\text{a}} = \frac{G_{\text{a}}+G_{\text{s}}}{2} + \frac{G_{\text{a}}-G_{\text{s}}}{2}\cos(4\varphi) + \beta_{\text{a-s}}\sin(4\varphi), \tag{S125a}$$
$$G'_{\text{s}} = \frac{G_{\text{a}}+G_{\text{s}}}{2} - \frac{G_{\text{a}}-G_{\text{s}}}{2}\cos(4\varphi) - \beta_{\text{a-s}}\sin(4\varphi), \tag{S125b}$$
$$\beta'_{\text{a-s}} = -\frac{G_{\text{a}}-G_{\text{s}}}{2}\sin(4\varphi) + \beta_{\text{a-s}}\cos(4\varphi), \tag{S125c}$$
$$\eta'_{\text{t}} = \frac{\eta_{\text{t}}+\eta_{\text{b}}}{2} + \frac{\eta_{\text{t}}-\eta_{\text{b}}}{2}\cos(4\varphi) + \beta_{\text{t-b}}\sin(4\varphi), \tag{S125d}$$
$$\eta'_{\text{b}} = \frac{\eta_{\text{t}}+\eta_{\text{b}}}{2} - \frac{\eta_{\text{t}}-\eta_{\text{b}}}{2}\cos(4\varphi) - \beta_{\text{t-b}}\sin(4\varphi), \tag{S125e}$$
$$\beta'_{\text{t-b}} = -\frac{\eta_{\text{t}}-\eta_{\text{b}}}{2}\sin(4\varphi) + \beta_{\text{t-b}}\cos(4\varphi), \tag{S125f}$$

The Mohr's circle presented in Figs. S11-c can be obtained by plotting G'_{a} against $\beta'_{\text{a-s}}$ for different values of φ. It is important to note that in this case, the argument of the sine and cosine functions is 4φ, unlike the force- and couple-stress Mohr's circles where it is 2φ. As a result, there exists a set of rotation angles, given by $1/4\left(\tan^{-1}(2\beta_{\text{a-s}}/(G_{\text{a}}-G_{\text{s}})) + \pi n\right)$, $n \in \mathbb{Z}$, for which the coupling parameter $\beta'_{\text{a-s}}$ becomes zero. At these angles, G'_{a} and G'_{s} reach their extreme values as presented in (S126). Similarly, by plotting η'_{t} against $\beta'_{\text{t-b}}$ for different values of φ, we obtain the Mohr's circle shown in Figs. S11-d. In this case, there also exist specific rotation angles, given by $1/4\left(\tan^{-1}(2\beta_{\text{t-b}}/(\eta_{\text{t}}-\eta_{\text{b}})) + \pi n\right)$, $n \in \mathbb{Z}$, at which the coupling parameter $\beta'_{\text{t-b}}$ becomes zero. At these angles, η'_{t} and η'_{b} reach their extreme values as presented in (S127).

$$G'_{\text{a}} = \frac{G_{\text{a}}+G_{\text{s}}}{2} \pm \sqrt{\beta^2_{\text{a-s}} + (G_{\text{a}}-G_{\text{s}})^2}, \tag{S126a}$$
$$G'_{\text{s}} = \frac{G_{\text{a}}+G_{\text{s}}}{2} \mp \sqrt{\beta^2_{\text{a-s}} + (\frac{G_{\text{a}}-G_{\text{s}}}{2})^2}, \tag{S126b}$$
$$\eta'_{\text{t}} = \frac{\eta_{\text{t}}+\eta_{\text{b}}}{2} \pm \sqrt{\beta^2_{\text{t-b}} + (\frac{\eta_{\text{t}}-\eta_{\text{b}}}{2})^2}, \tag{S127a}$$
$$\eta'_{\text{b}} = \frac{\eta_{\text{t}}+\eta_{\text{b}}}{2} \mp \sqrt{\beta^2_{\text{t-b}} + (\frac{\eta_{\text{t}}-\eta_{\text{b}}}{2})^2}, \tag{S127b}$$

S5.2.3 Orthorhombic crystals

The constitutive matrix in Voigt notation corresponding to the metaplates with unit cells belonging to the crystal class No.6 (mmm) is presented below

$$\begin{bmatrix} C_{11} & C_{12} & \cdot & \cdot & \cdot & \cdot & \cdot & \cdot & \cdot \\ & C_{22} & \cdot & \cdot & \cdot & \cdot & \cdot & \cdot & \cdot \\ & & C_{44} & \cdot & \cdot & \cdot & \cdot & \cdot & \cdot \\ & & & D_{11} & \bar{D}_{11} & \cdot & \cdot & \cdot & \cdot \\ & & & & D_{11} & \cdot & \cdot & \cdot & \cdot \\ & & & & & D_{44} & D_{45} & \cdot & \cdot \\ & & & & & & D_{55} & \cdot & \cdot \\ & & & & & & & D_{77} & \cdot \\ & & & & & & & & D_{99} \end{bmatrix} \tag{S128}$$

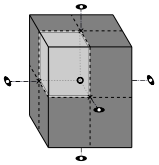

(S128) contains four independent material parameters of the C tensor, and six material parameters of the D tensor. One may find the bulk, axial, synclastic and anticlastic moduli, yet since the associated modes of deformation are not decoupled anymore they only make everything more complicated. Therefore, for this class of material we skip those formulations and go straight to the compliance relations as presented in (S129).

$$\begin{bmatrix} \hat{\epsilon}_{11} \\ \hat{\epsilon}_{22} \\ \hat{\epsilon}_{12} \\ \hat{\kappa}_{11} \\ \hat{\kappa}_{22} \\ \hat{\kappa}_{12} \\ \hat{\kappa}_{21} \\ \hat{\kappa}_{31} \\ \hat{\kappa}_{32} \end{bmatrix} = \begin{bmatrix} \frac{1}{E_1} & \frac{-\nu_{12}}{E_1} & \cdot & \cdot & \cdot & \cdot & \cdot & \cdot & \cdot \\ \frac{-\nu_{21}}{E_2} & \frac{1}{E_2} & \cdot & \cdot & \cdot & \cdot & \cdot & \cdot & \cdot \\ & & \frac{1}{4G_s} & \cdot & \cdot & \cdot & \cdot & \cdot & \cdot \\ & & & \frac{1}{4\eta_t} & -\frac{1}{4\eta_t} & \cdot & \cdot & \cdot & \cdot \\ & & & & \frac{1}{4\eta_t} & \cdot & \cdot & \cdot & \cdot \\ & & & & & \frac{1}{I_1} & \frac{\varsigma_{12}}{I_1} & \cdot & \cdot \\ & & & & & \frac{\varsigma_{21}}{I_2} & \frac{1}{I_2} & \cdot & \cdot \\ & & & & & & & \frac{1}{I_{31}} & \cdot \\ & & & & & & & & \frac{1}{I_{32}} \end{bmatrix} \cdot \begin{bmatrix} \hat{\sigma}_{11} \\ \hat{\sigma}_{22} \\ 2\hat{\sigma}_{12} \\ \hat{\mu}_{11} \\ \hat{\mu}_{22} \\ \hat{\mu}_{12} \\ \hat{\mu}_{21} \\ \hat{\mu}_{31} \\ \hat{\mu}_{32} \end{bmatrix} \tag{S129}$$

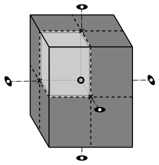

The relationship among the constitutive and compliance parameters are presented below

$$C_{11} = \frac{E_1^2}{E_1 - E_2\nu_{12}^2}, \tag{S130a}$$

$$C_{22} = \frac{E_1E_2}{E_1 - E_2\nu_{12}^2}, \tag{S130b}$$

$$C_{12} = \frac{E_1E_2\nu_{12}}{E_1 - E_2\nu_{12}^2}, \tag{S130c}$$

$$E_1 = -\frac{C_{12}^2 - C_{11}C_{22}}{C_{22}}, \tag{S130d}$$

$$E_2 = -\frac{C_{12}^2 - C_{11}C_{22}}{C_{11}}, \tag{S130e}$$

$$\nu_{12} = \frac{C_{12}}{C_{22}}, \tag{S130f}$$

$$D_{44} = \frac{I_1^2}{I_1 - I_2\varsigma_{12}^2}, \tag{S130g}$$

$$D_{55} = \frac{I_1I_2}{E_1 - E_2\varsigma_{12}^2}, \tag{S130h}$$

$$D_{45} = -\frac{I_1I_2\varsigma_{12}}{I_1 - I_2\nu_{12}^2}, \tag{S130i}$$

$$I_1 = -\frac{D_{45}^2 - D_{44}D_{55}}{D_{55}}, \tag{S130j}$$

$$I_2 = -\frac{D_{45}^2 - D_{44}D_{55}}{D_{44}}, \tag{S130k}$$

$$\varsigma_{12} = -\frac{D_{45}}{D_{55}}. \tag{S130l}$$

S6 Supplementary results

S6.1 AHH results

The Tables S5 and S6 present the effective material properties derived from the analysis of 1D-AAH and 2D-AAH, respectively, considering a range of values for n from one to fifteen. It is evident that with increasing values of n, the effective CSBT and CSPT material properties tend to converge towards the properties predicted by classical Euler-Bernoulli beam theory and Love-Kirchhoff plate theory, respectively. The one-dimensional Young's modulus ($E^{1\mathrm{D}}$) tend to the classical 3D Young's modulus ($E^{\mathrm{C}} = (9K^{3\mathrm{D}}G_a^{3\mathrm{D}})/(G_a^{3\mathrm{D}}+3K^{3\mathrm{D}}) = 2.116\%E^0$), one-dimensional Cosserat modulus ($I^{1\mathrm{D}}$) tends to the bending modulus per unit area ($E^{\mathrm{C}}th^2/12 = 0.176\%E^0th^2$), and torsional modulus ($\eta_t^{1\mathrm{D}}$) tends to the classical torsional rigidity of a homogeneous prismatic bar with a square cross section of size $th \times th$ ($\eta_t^{\mathrm{C}} = 0.141G_s^{3\mathrm{D}}th^2 = 0.145\%E^0th^2$). Finally, the axial-torsional coupling modulus tends to zero as the size of unit cells approaches zero (n reaches infinity). The two-dimensional bulk modulus ($K^{2\mathrm{D}}$) tend to the classical plane-stress bulk modulus ($E^{\mathrm{C}}/2(1-\nu^{3\mathrm{D}}) = 1.205\%E^0$), two-dimensional deviatoric modulus ($G^{2\mathrm{D}}$) tends to its classical companion in plane-stress state ($G_a^{3\mathrm{D}} = 0.943\%E^0$), two-dimensional torsional modulus ($\eta_t^{2\mathrm{D}}$) and deviatoric bending modulus ($\eta_b^{2\mathrm{D}}$) tend to their pertinent modulus in the Love-Kirchhoff theory for a homogeneous plate with a thickness of th ($\eta_t^{2\mathrm{D}} \to G_s^{3\mathrm{D}}th^2/12 = 0.086\%E^0th^2$ and $\eta_b^{2\mathrm{D}} \to G_a^{3\mathrm{D}}th^2/12 = 0.078\%E^0th^2$). Finally, the axial-torsional ($\beta_{a\text{-}t}^{2\mathrm{D}}$) and shear-bending ($\beta_{s\text{-}b}^{2\mathrm{D}}$) coupling moduli as well as the in-plane bending modulus ($I_3^{2\mathrm{D}}$) disappear as the size of unit cells goes to zero.

S6.2 Mindlin's ratio

To investigate Mindlin's ratio, five different symmetry classes, namely No.21 ($6/mmm$), No.24 ($6/m$), No.14 ($4/mmm$), No.17 ($4/m$), and No.6 (mmm) are considered. The unit cells possessing 6-fold symmetry ($6/mmm$ and $6/m$) are isotropic in the context of CSPT, and only five material parameters are required to describe their behaviour; two for in-plane stress-strain relationship(K and G), two for out-of-plane couple-stress-curvature behaviour (η and γ), and one for in-plane couple-stress-curvature relation (I_3). In order of symmetry, the next class is ($4/mmm$) which demands two additional material parameters, one for in-plane shear (G_s) and the other for the out-of-plane torsion (η_t). Next is ($4/m$), which displays two interesting couplings: one between shear and deviatoric stresses and the other one between torsion and symmetric bending couple-stress. These couplings are characterized by β_{s-d} and β_{t-b}, respectively. The least symmetry among the selected classes belongs to (mmm). This symmetry class is similar to ($4/mmm$) except that the areal and deviatoric part of stress and symmetric and antisymmetric part of couple-stress are coupled. Other than the mentioned coupling, these additional material parameters make the unit cell orthotropic where the Young's and Cosserat moduli in two principal directions become different ($E_1 \neq E_2$ and $I_1 \neq I_2$). As a consequence, the Poisson's and Mindlin's ratios in two principal directions become different ($\nu_{12} \neq \nu_{21}$ and $\varsigma_{12} \neq \varsigma_{21}$), yet they have a relation with the Young's and Cosserat modulus ratio ($\nu_{12} = \nu_{21}E_1/E_2$ and $\varsigma_{12} = \varsigma_{21}I_1/I_2$). Positive definiteness of the generalized elasticity tensor dictates the following relations: for the unit cells with ($6/mmm$), ($6/m$), and ($4/mmm$) symmetries $|\nu| < 1$ and $|\varsigma| < 1$, for 2D-tetrachiral ($4/m$) unit cells $|\beta_{s-a}| < \sqrt{(G_sG_a)}$ and $|\beta_{t-b}| < \sqrt{(\eta_t\eta_b)}$, and for the orthotropic (mmm) unit cells $|\varsigma_{12}| < \sqrt{(I_1/I_2)}$ and $|\varsigma_{21}| < \sqrt{(I_2/I_1)}$.

We investigate the influence of relative thickness ($\bar{th} = \ell/th$) and a geometric parameter (ϕ) on the variation of Mindlin's ratio for three different unit cells: honeycomb (mmm), star-reentrant ($4/mmm$), and 2D-tetrachiral ($4/m$). In Fig. S12-a, we present the variation of ς_{12} and ς_{21} with $\bar{th}$ and ϕ for a metaplate constructed using the honeycomb unit cell (mmm). Notably, the anisotropic nature of the honeycomb unit cell enables achieving Mindlin's ratios up to 3, which is considerably higher than the values associated with unit cells having 6-fold and 4-fold symmetries. The latter exhibit an extremum of ±1 for Mindlin's ratio. Furthermore, Fig. S12-b displays the upper and lower bounds of ς_{12} and ς_{21} with respect to $\bar{th}$ and φ for honeycomb metaplates. Moving on to Fig. S12-c, we examine the variation of Mindlin's and Poisson's ratios for metaplates composed of star-reentrant unit cells in relation to $\bar{th}$ and φ. Although Poisson's ratio shows only slight variation with relative thickness, the variation in Mindlin's ratio is significant. It is also apparent that for larger values of relative thickness, Mindlin's ratio tends to converge towards Poisson's ratio. Finally, Fig. S12-d illustrates the variation of Mindlin's ratio with $\bar{th}$ and ϕ along the two principal directions. Notably, it is important to recognize that the principal orientations differ for each combination of $\bar{th}$ and ϕ.

S6.3 Validating Mindlin's Ratio via FEM and Experiments

The Mindlin ratio, depicted in Fig. 4(a) of the main text, is defined as the ratio of induced curvature in the transverse direction when a uniform couple-stress (bending moment) is applied in the direct direction. More specifically, assuming that the plate is subject to uniform couple stress $\hat{\mu}_{12}$, the Mindlin ratio, ς, is defined as:

$$\varsigma = \frac{\hat{\kappa}_{21}}{\hat{\kappa}_{12}} \tag{S131}$$

In order to accurately ascertain Mindlin's ratio, defined herewith, a plate needs to be subjected to couple-stress across two opposing boundaries without the influence of additional forces, a scenario referred to as a free boundary condition throughout this text. The experimental emulation of such a loading condition inherently presents a challenge. To navigate this, we have affixed two rigid segments to the metaplates' ends, enabling viable experimentation. The introduction of these rigid elements, however, effectively suppresses transverse

curvature at the boundaries—termed a fixed boundary condition in the subsequent discussion. This modification could induce discrepancies between the observed double curvature in the plate and the computed effective Mindlin's ratio. Nevertheless, as demonstrated in the manuscript, this boundary condition does not invert the sign of the ensuing double curvature, allowing us to validate our model's predictions through experimental means. It's imperative to recognize that we do not expect experimental results to precisely emulate the curvatures predicted through homogenization. The affirmation of the experimental and homogenization results necessitates their juxtaposition with distinct simulations: one adhering to a fixed, and the other to a free boundary condition. This comparative analysis is elucidated in Fig. S13 and Table S9.

Pivoting from the aforementioned discussion and results, the discrepancy observed between the homogenization and experimental outcomes can be ascribed to the differential boundary conditions. In the experiment, metaplates with unit cell orientations of $\varphi^D = 33^o$ and $\varphi^D = -12^o$ are restrained from rotation and displacement at both ends, whilst the theoretical results presuppose free boundary conditions. It is vital to underscore that the primary intent of this experiment is to ascertain the sign of Mindlin's ratio. This allows for the categorization of the bending shape of various plates and infuses fresh perspectives into the domain. Traditionally, the double-curvature shape of plates under unidirectional bending has been associated with Poisson's ratio. This investigation, however, introduces Mindlin's ratio as a determinant, singularly governing the curvature ratios and demarcating the synclastic and anticlastic form of a metaplate under bending.

S7 Experiments

This section provides a comprehensive overview of the experimental procedures. Firstly, the preparation processes are discussed, encompassing the 3D printing of the samples, fabrication of necessary fixtures, and the setup required for conducting the experiments.

S7.1 Additive manufacturing

S7.1.1 SLS 3D printing and material properties

The samples were produced using selective laser sintering (SLS) printing technology. To initiate the printing process, a thin layer of polymer powder with a particle size of $60\mu m$ was evenly spread across the build platform. The maximum build size achievable was $25.4\,cm \times 25.4\,cm \times 34.29\,cm$, with a resolution of $0.75\,mm$ in the X/Y plane and $0.1\,mm$ in the Z direction. The nominal minimum thickness allowed was $1\,mm$, and any features with a layer thickness smaller than $1\,mm$ needed to be scaled up. A 50W CO_2 laser in the Sinterstation 2500 was employed to selectively sinter the particles based on the sliced pattern derived from the design's STL file, created using SolidWorks. The printing process involved successive layering, with the blade laying fresh powder on the build area after each layer was completed.

Given the open-cell structure of the design, any unsintered powder remaining inside the printed sample was removed using a brush and further polished using a sandblaster and glass beads blasting. This post-processing procedure took approximately two hours to ensure the high quality of the final 3D printed samples. The mechanical properties of the TPU material used were experimentally determined by Shi et al. [25].

S7.1.2 FDM 3D printing of the fixtures

Fused deposition modeling (FDM) 3D printers operate by extruding thermoplastic filaments through a heated nozzle, melting the material and applying it layer by layer onto a build platform. Each layer is added successively until the entire part is completed. In this research, we utilized FDM printing to create fixtures for two different purposes: beam and plate tests.

For the beam tests, the fixtures were designed to provide fixed boundary conditions during the experiments. In the plate tests, the fixture was specifically designed to apply shear deformations to the plate sample. The load test machine applied axial displacement, and by utilizing the fixtures, the applied deformations were converted into shear deformation.

In our study, an Ultimaker S3 printer and PLA material were used. The fixtures were printed with an infill density of 80% and an infill line pattern with directions of $[45°, -45°]$ to ensure rigidity. To enhance printing quality, the print speed was set to $70mm/s$, and the printing temperature was maintained at 210°C. Additionally, water-soluble PVA material was employed to print the necessary supports.

S7.2 3D printed samples

Fig. S14 presents images of 3D printed chiral beams with left-handed (LH) and right-handed (RH) orientations, ranging from $n = 1$ to $n = 5$, while maintaining a constant $N = 2$. Similarly, Fig. S15 showcases 3D printed chiral and 2D-tetrachiral plates with three distinct orientations: $\varphi = 0°$, $\varphi = -12°$, and $\varphi = 33°$.

S7.3 Test procedure

Tests were conducted using a dual column ADMET eXpert 8612 universal testing machine with a capacity of 20 kN. The tests were performed under displacement and rotation rates of 10 mm/min and 10 deg/min, respectively.The measurements were performed by applying 0.10 and $1.75\ 1/m$ axial strain and axial curvature, respectively. The deviations from linearity observed in the tests were minimal. To ensure the accuracy and reliability of the results, each test was repeated three times, effectively reducing any deviations. The reported results represent the averages obtained from these three repeated tests.

S7.3.1 Beams

For the experimental validation of the behavior of chiral metabeams, four load cases were considered, as illustrated in Fig. 2c of the main manuscript. The experimental setup for these load cases is shown in Fig. S16a. In load cases (1) and (3), right-handed metabeams were connected to identical left-handed metabeams to replicate the assumed free boundary conditions in the numerical analysis. Consequently, when an axial (or twist) deformation was applied to the top of the metabeam, the connecting surface was allowed to rotate (or vertically displace) freely. In load cases (2) and (4), right-handed and left-handed metabeams were tested separately, with their bottom face fixed, and the deformation applied to their top faces. To accurately capture the deformations, 3D digital image correlation (DIC) was employed. Forces were extracted from a 20 kN dual-column ADMET eXpert 8612 universal testing machine.

S7.3.2 Plates

The experimental setup for capturing the shear-bending coupling in a chiral metaplate is depicted in S16-b. The plate is screwed to two rigid (relative to the plate) fixtures on the left and right sides. The right-side fixture is fixed against rotation and displacement at the bottom gripper of the testing machine, and the testing machine applies a vertical displacement to the left-side fixture. This mechanism due to the off-axis placement of the fixtures imposes a shear deformation to the metaplate and out-of-plane bending deformation is captured using DIC techniques.

S7.4 Digital image correlation

Digital image correlation (DIC) is a non-contact optical technique for studying the mechanical deformation of solids, which can obtain the deformation of a surface by comparison of digital images of the undeformed and deformed configurations. Subset based DIC technique is the most widely used method in which, by tracking blocks of pixels, the system can measure the surface displacement and build up the whole deformation fields. First, a reference square subset with sufficient intensity is selected from the undeformed image. Second, based on the predefined correlation criterion along with the computational algorithm, the software starts to search for the deformed image corresponding to the square subset in the undeformed image. The target subset is selected based on the maximum similarity in intensity pattern with the reference subset. Third, the difference between the target subset and the reference subset, the displacement vector, is calculated. So, to effectively utilize DIC and obtain reliable matching, the subsets need to be unique with an acceptable range of contrast and intensity levels to be identified in the deformed image, which is directly related to the speckle patterns. The speckle patterns can be artificially created by spraying the white/black paints or manually specifying the points with a marker. The errors of the obtained deformation field are directly related to the speckle pattern's quality [9, 13, 28]. Generally, the speckle size must be at a minimum $3-5$ pixels in size and the ratio of speckle areas to the desired surface area of the study should be 0.5.

In this study, a VIC 3D Correlated Solutions Inc. DIC system was utilized, consisting of two illumination sources and two 5 MP cameras equipped with 35 mm lenses. The system was mounted on a leveled stand and positioned in front of the experimental setup, as illustrated in Figure S17. The setup was calibrated separately for beam and plate tests, ensuring accurate measurements for each configuration. VIC-3D software was employed for the data analysis. A subset size of 11×11 pixels was chosen to encompass sufficient dominant features within a single subset, as suggested by McGinnis et al. [17]. It is important to consider the trade-off between subset size, distinguishability, and accuracy. Larger subset sizes provide distinct patterns that can be easily distinguished from neighboring subsets, while smaller subset sizes accurately capture the underlying deformation field using first/second order subset shape functions. To enhance the spatial resolution and data density, the step between subsets was set to 7 pixels during the analysis process, allowing for a more detailed representation of the captured data.

S7.5 Sources of error in experiment

The manufacturing process of 3D-printed samples is subject to various potential sources of error, including factors such as printing accuracy, imperfections in the 3D-printed objects, and post-processing procedures. In this context, the samples were manufactured utilizing SLS (Selective Laser Sintering) 3D printing technology, employing TPU (Thermoplastic Polyurethane) material. Referring to section S7.1.1, the 3D printer's resolution in the X/Y plane is $0.75mm$, while the resolution in the Z direction is $0.1mm$. Additionally, the minimum nominal thickness is set at $1.0mm$. As a result, it is inevitable that there will be a slight discrepancy between the dimensions and density of the design and the actual 3D-printed samples, as outlined in Tables S7 and S8 for metabeams and metaplates, respectively. This deviation can become more pronounced when dealing with smaller features in the samples, such as beams with $n = 5$, which exhibit the greatest differences in geometric parameters and weight between the as-fabricated and as-designed states. Furthermore, inherent geometric defects in the 3D-printed samples contribute to elastic imperfections [16], as illustrated in Fig. S18 through SEM imaging of a chiral unit cell. It has been demonstrated that accounting for these imperfections in simulations can yield significantly more accurate results, with differences of up to 50 observed between the simulations of perfect and imperfect designs [7, 16]. This underscores the substantial impact that printing imperfections can have on material properties, contingent upon the specific design and printing techniques employed. After the SLS printing process, any remaining unsintered powder within the printed samples was meticulously removed using brushes and then further refined through sandblasting and glass bead blasting procedures, all accomplished within a two-hour timeframe. Although it is expected that beam samples (ranging from $n = 1$ to $n = 5$, both LH and RH) should possess identical weights after post-processing, it is evident that samples featuring smaller details, i.e., LH and RH with $n = 4$ and $n = 5$, exhibit higher weights in comparison to LH and RH beams with $n = 1$, as illustrated in Table S7. This discrepancy serves as concrete evidence that residual unsintered powder persists within the samples despite the completion of post-processing. Hence, depending on the size and intricacy of features in each design, the presence of unsintered powder may endure, necessitating manual removal, which cannot guarantee an exceptionally precise outcome. All the aforementioned factors may collectively contribute to deviations between experimental and theoretical results.

Supplementary Figures

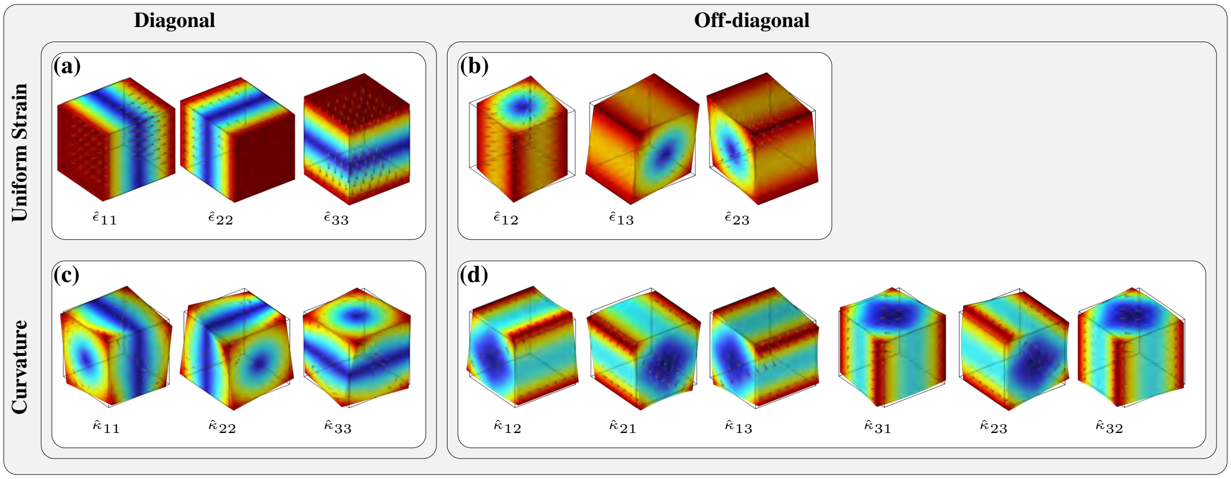

Figure S1: Deformation of a cubic unit cell pertinent to 3D-AAH when three pairs of periodic boundary conditions and volumetric strains associated to each case is applied. (a) Diagonal uniform strains representing axial deformation, (b) Off-diagonal uniform strains representing shear deformations, (c) Strains associated with diagonal curvatures representing twisting deformations, and (d) Strains associated with off-diagonal curvatures representing bending deformation.

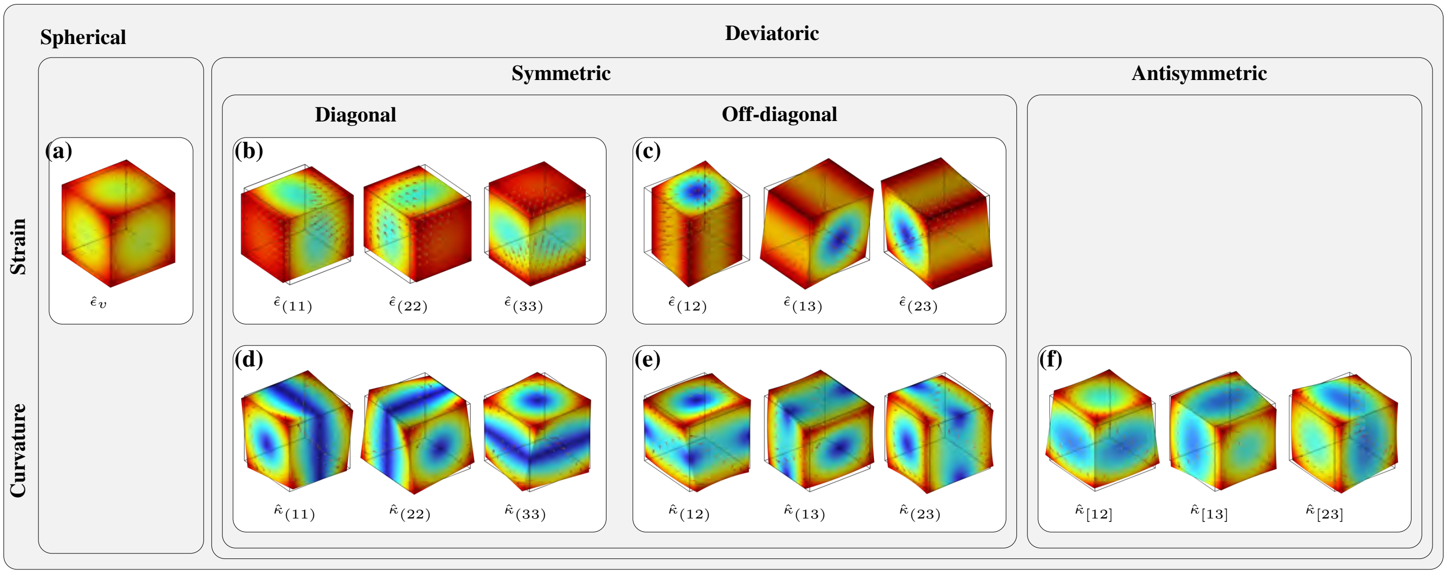

Figure S2: Deformation of a cubic unit cell pertinent to 3D-AAH when three pairs of periodic boundary conditions and volumetric strains associated to each case of deformation is applied. (a) Spherical strain representing uniform volumetric deformation, (b) Diagonal deviatoric uniform strains, (c) Off-diagonal deviatoric uniform strains representing shear deformations, (d) Strains associated with diagonal symmetric deviatoric curvatures representing twisting deformations, (e) Strains associated with off-diagonal symmetric deviatoric curvatures representing anticlastic (saddle-shape) bending deformation, and (f) Strains associated with off-diagonal anti-symmetric deviatoric curvatures representing synclastic (dome-shape) bending deformation.

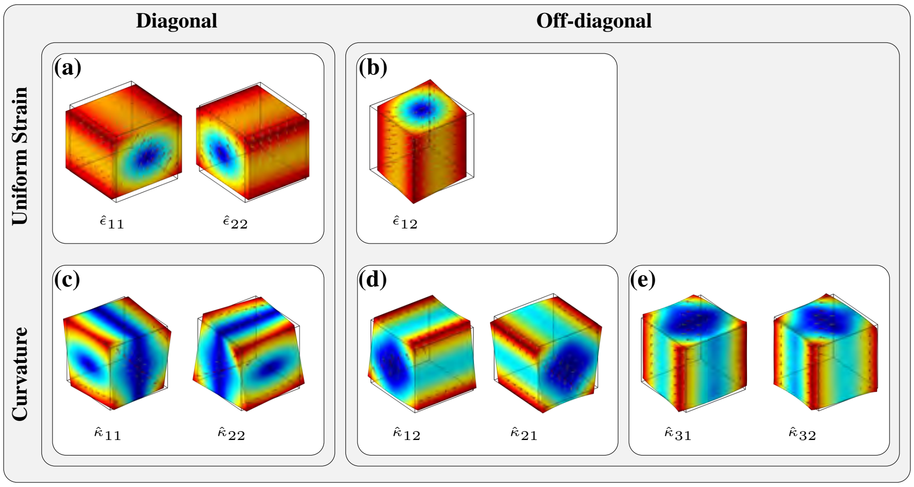

Figure S3: Deformation of a cubic unit cell pertinent to 2D-AAH when two pairs of periodic boundary conditions and volumetric strains associated to each case of deformation is applied. (a) Diagonal uniform strains representing axial deformation, (b) Off-diagonal uniform strains representing shear deformations, (c) Strains associated with diagonal curvatures representing twisting deformations, (d) Strains associated with off-diagonal curvatures representing bending deformation, nad (e) Strains associated with in-plane curvatures.

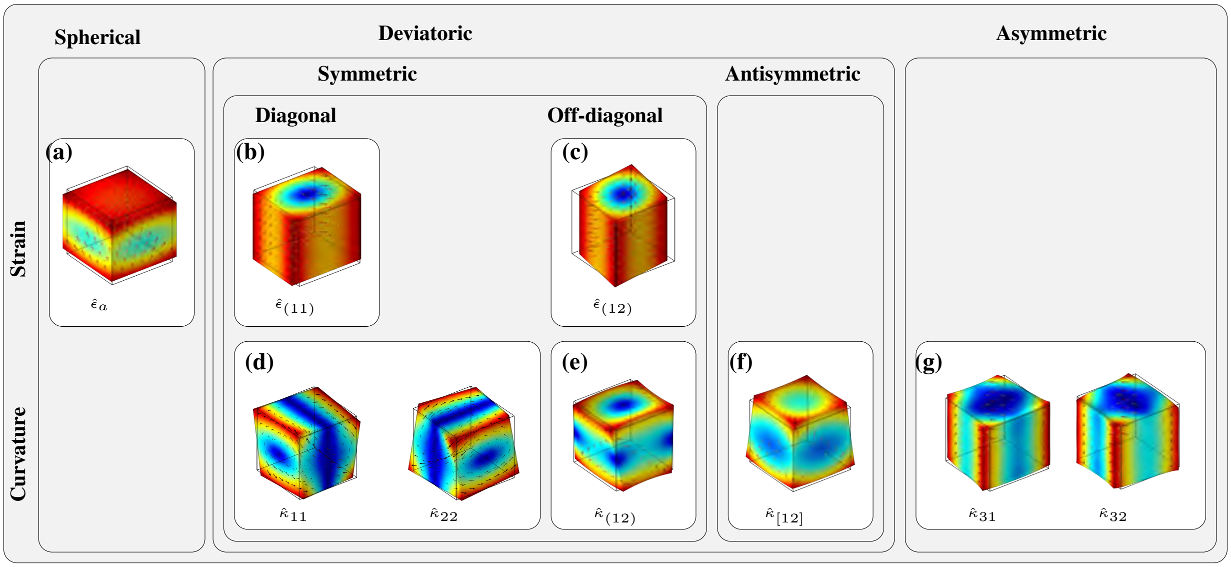

Figure S4: Deformation of a cubic unit cell pertinent to 2D-AAH when two pairs of periodic boundary conditions and volumetric strains associated to each case of deformation is applied. (a) Spherical strain representing uniform areal deformation, (b) Diagonal deviatoric uniform strains, (c) Off-diagonal deviatoric uniform strains representing shear deformations, (d) Strains associated with diagonal symmetric deviatoric curvatures representing twisting deformations, (e) Strains associated with off-diagonal symmetric deviatoric curvatures representing anticlastic (saddle-shape) bending deformation (f) Strains associated with off-diagonal anti-symmetric deviatoric curvatures representing synclastic (dome-shape) bending deformation, and (g) Strains associated with in-plane curvatures.

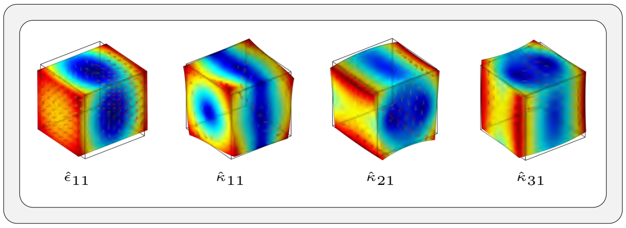

Figure S5: Deformation of a cubic unit cell pertinent to 1D-AAH when one pair of periodic boundary conditions and volumetric strains associated to each case of deformation is applied.

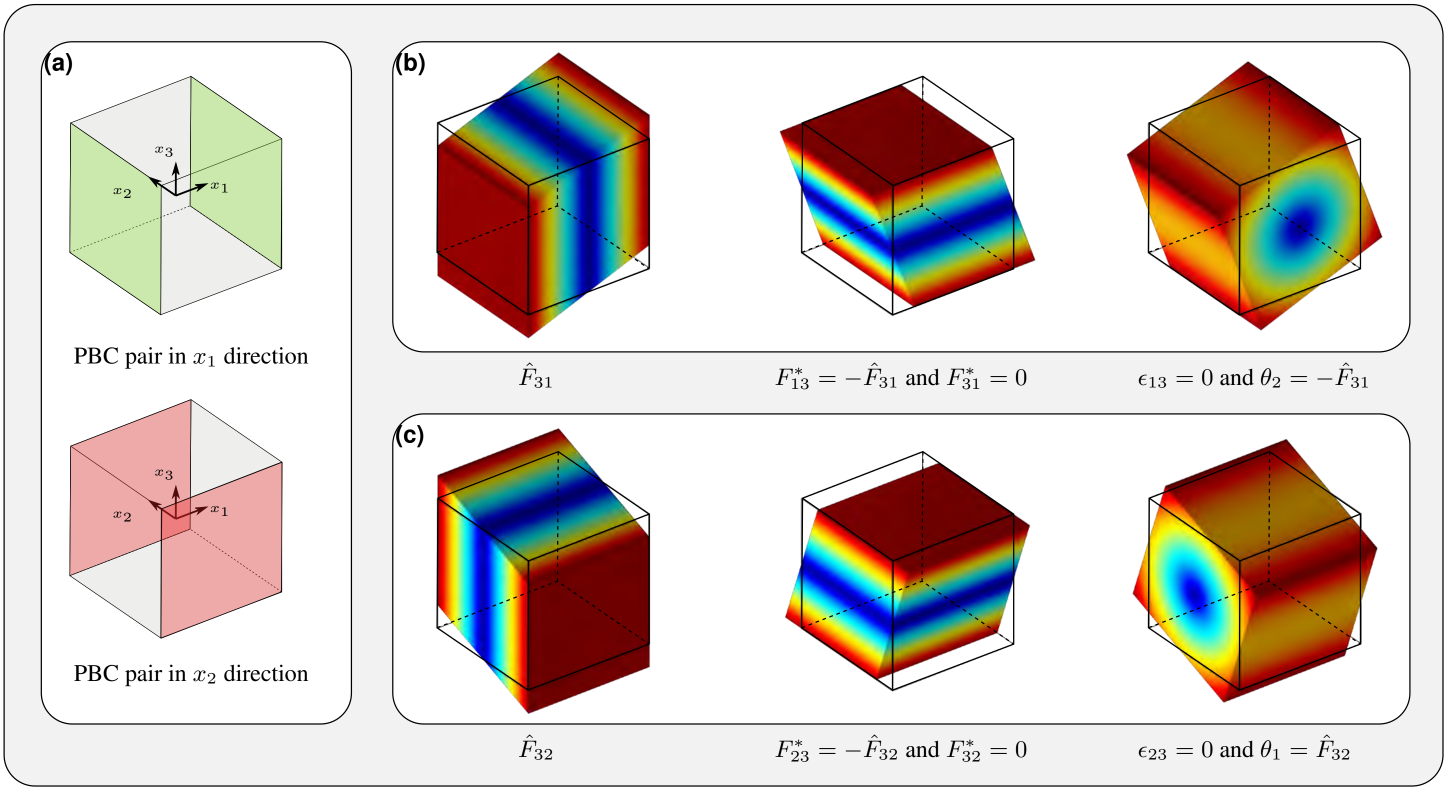

Figure S6: The macroscopic deformation gradients $\hat{F}_{31}$ and $\hat{F}_{32}$ are applied to a cubic homogeneous unit cell while two pairs of periodic boundary conditions (PBCs) are applied. Due to the absence of third periodic boundary condition in x_3 direction, the unit cell has the freedom to deform in a way that does not produce any total strain ($\boldsymbol{\epsilon}$) and results in only a rotation. (a) Periodic boundary conditions in CSPT, (b) Macroscopic, Periodic microscopic, and total deformation associated with the load case $\hat{F}_{31}$, and, (c) Macroscopic, Periodic microscopic, and total deformation associated with the load case $\hat{F}_{32}$.

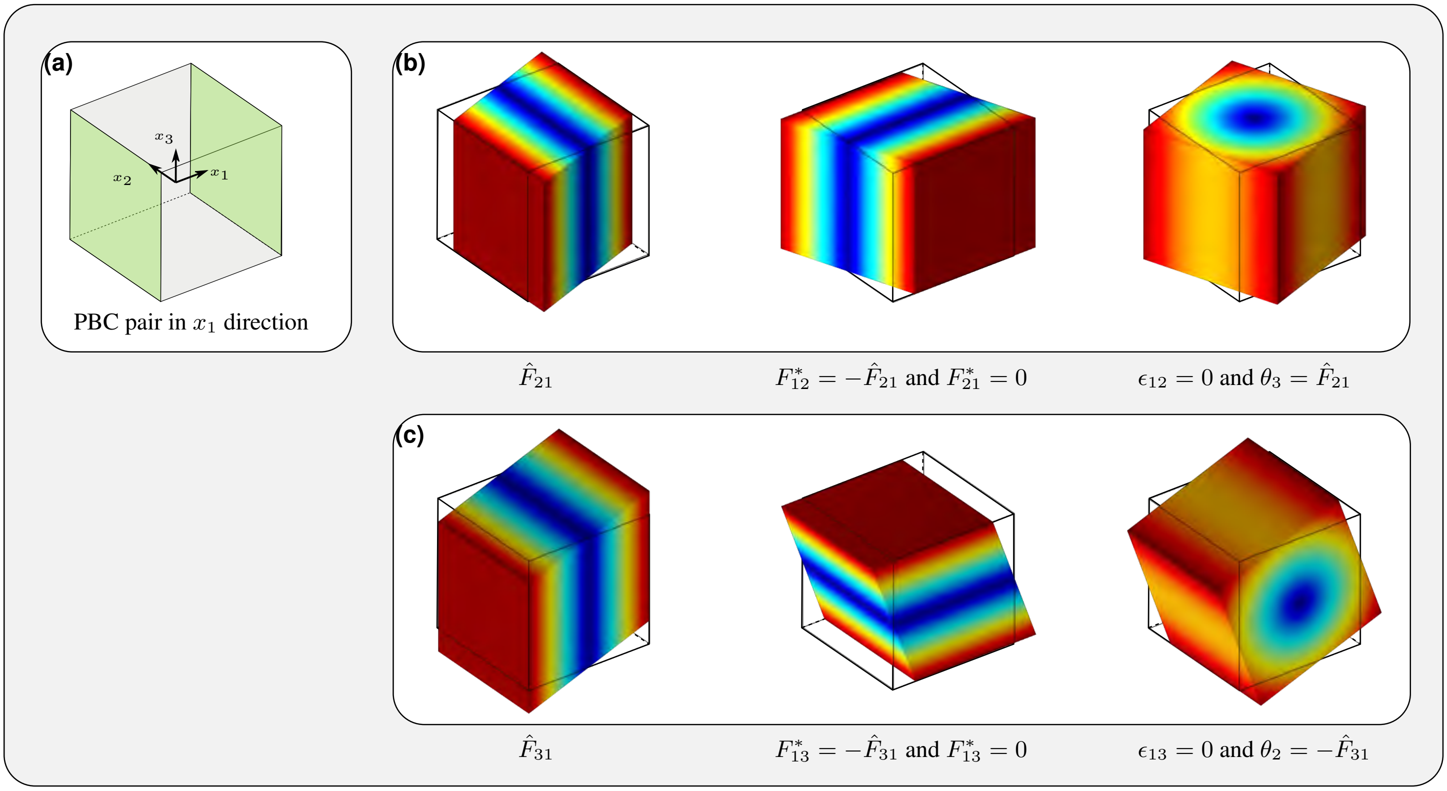

Figure S7: The macroscopic deformation gradients $\hat{F}_{21}$ and $\hat{F}_{31}$ are applied to a cubic homogeneous unit cell while one pair of periodic boundary condition (PBC) is applied. Due to the absence of the other two periodic boundary conditions in x_2 and x_3 directions, the unit cell has the freedom to deform in a way that does not produce any total strain (ϵ) and results in only a rotation. (a) Periodic boundary condition in CSBT, (b) Macroscopic, Periodic microscopic, and total deformation associated with the load case $\hat{F}_{21}$, and, (c) Macroscopic, Periodic microscopic, and total deformation associated with the load case $\hat{F}_{31}$.

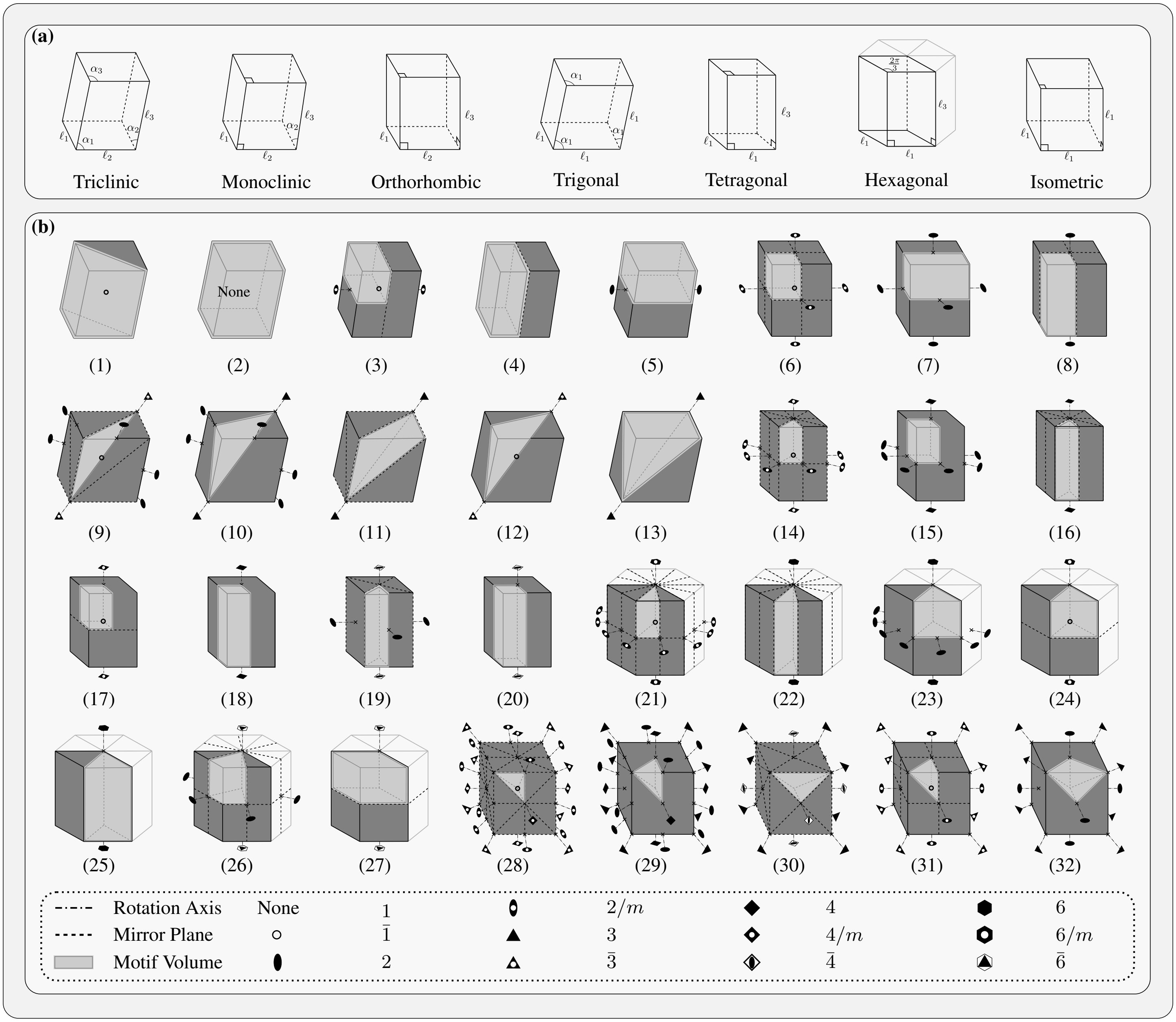

Figure S8: (a) Seven crystal systems, and (b) Thirty-two crystal classes.

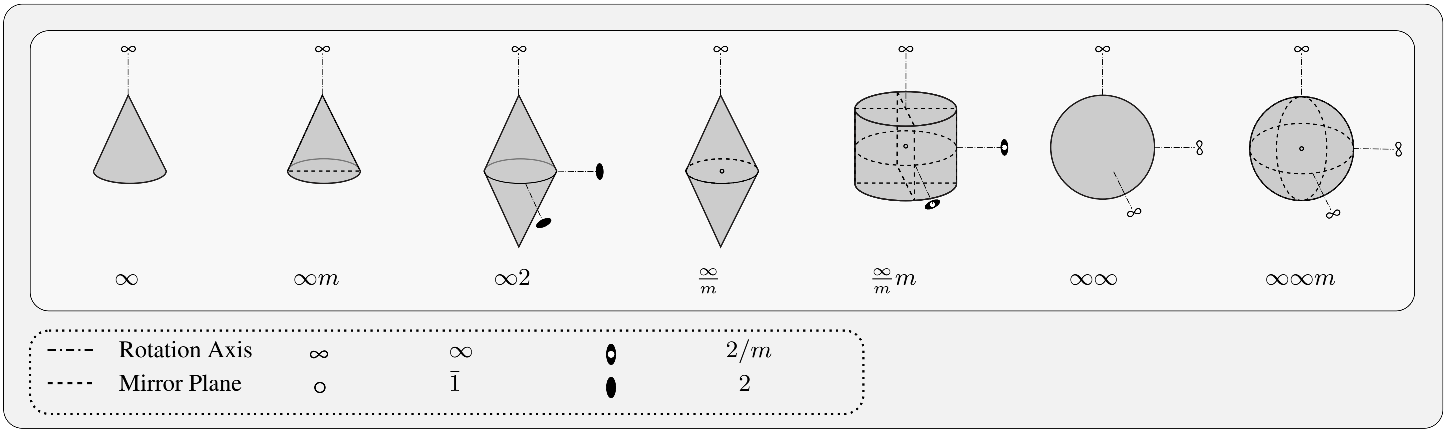

Figure S9: The Seven Curie symmetry groups.

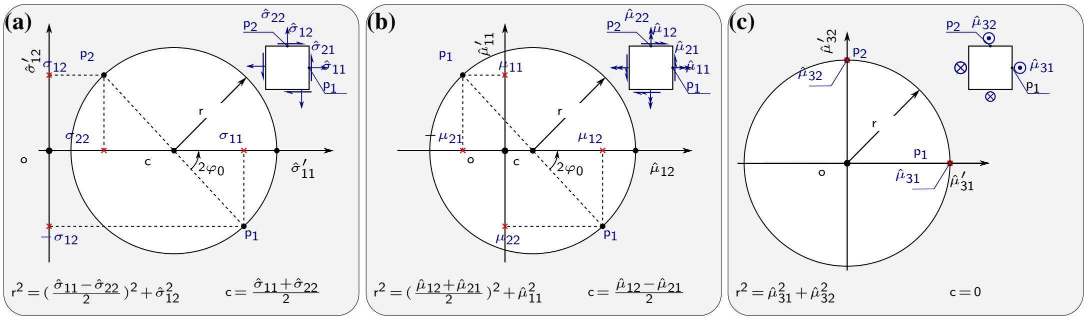

Figure S10: Mohr's circles associated with (a) force-stress components, (b) out-of-plane couple-stress components, and (c) in-plane couple-stress components.

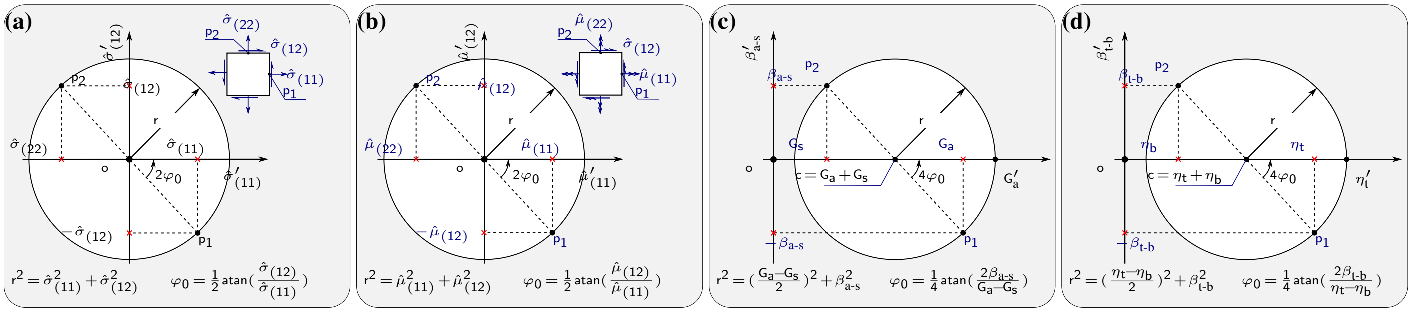

Figure S11: Mohr's circles associated with (a) symmetric-deviatoric force-stress components, (b) symmetric-deviatoric couple-stress components, (c) axial-shear coupling material properties, and (d) twist-bending coupling material properties.

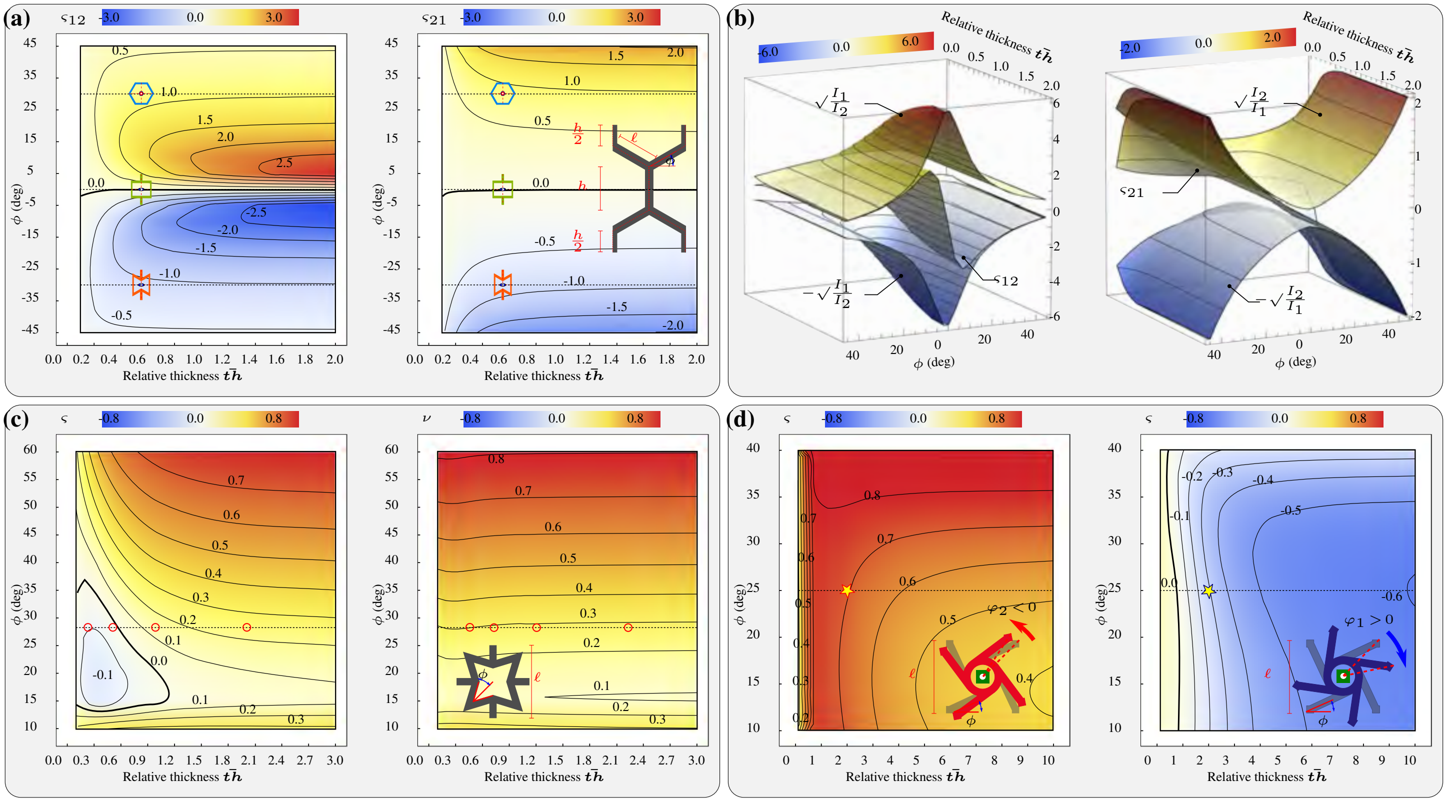

Figure S12: Mindlin's ratio in CSPT, (a) Variation of Mindlin's ratio in two directions (ς_{12} and ς_{21}) with the relative thickness and characteristic angle of the honeycomb, (b) Variation of Mindlin's ratio in two directions (ς_{12} and ς_{21}) with the relative thickness and characteristic angle of the honeycomb compared to their lower and upzper limits, (c) Variation of Mindlin and Poisson's ratios with the relative thickness and characteristic angle of the auxetic star-shaped ($4/mmm$) unit cell, (d) Variation of Mindlin's ratio in two principal orientations with the relative thickness and characteristic angle of tetra-chiral unit cell.

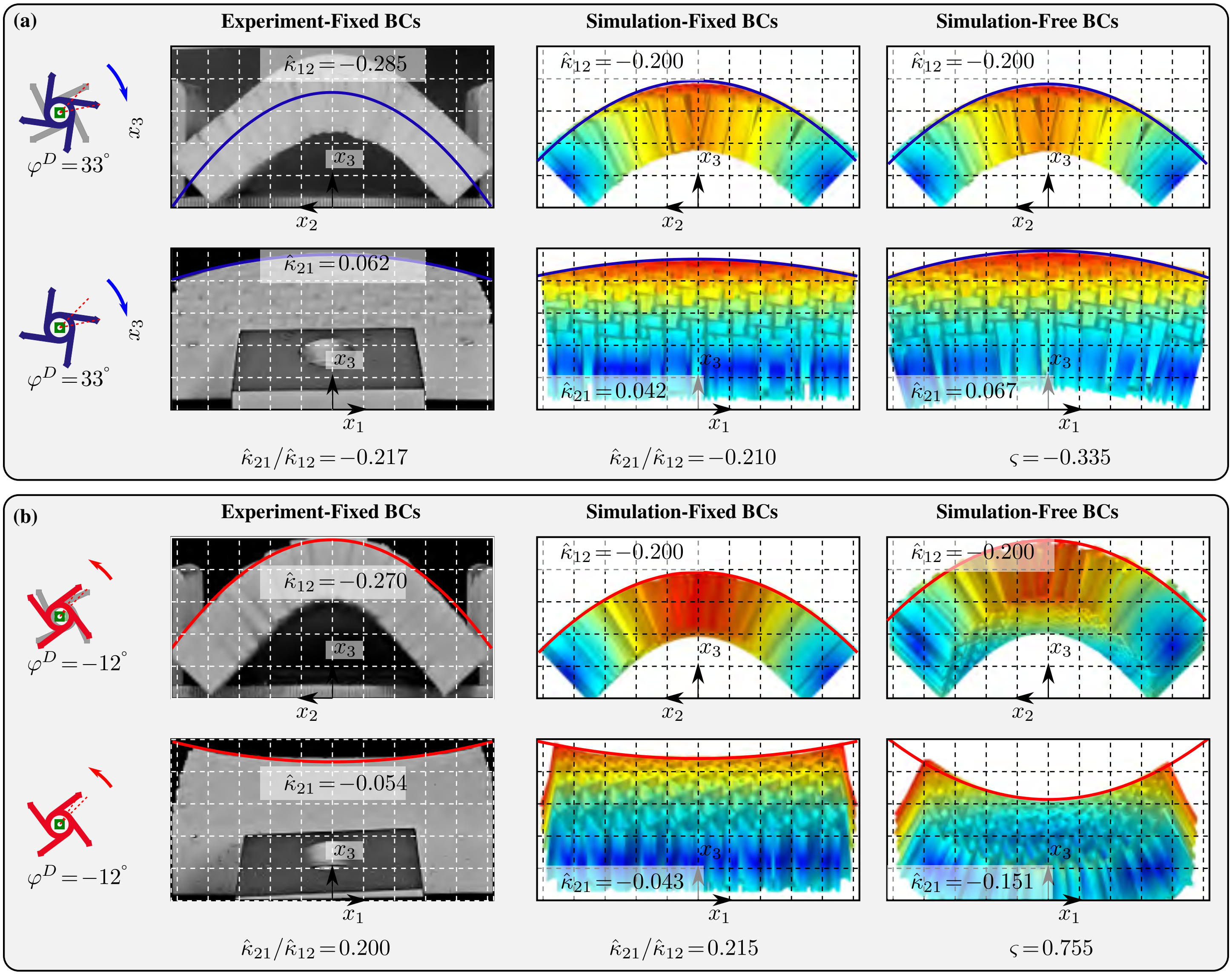

Figure S13: Comparative Analysis of the Double Curvature Ratio in 2D-Tetrachiral Metaplates: Utilizing Experimental and Detailed FEM Simulations for Configurations of 10×10 2D-Tetrachiral Unit Cells at Two Distinct Orientations, (a) $\varphi^D = 33^o$ and (b) $\varphi^D = -12^o$

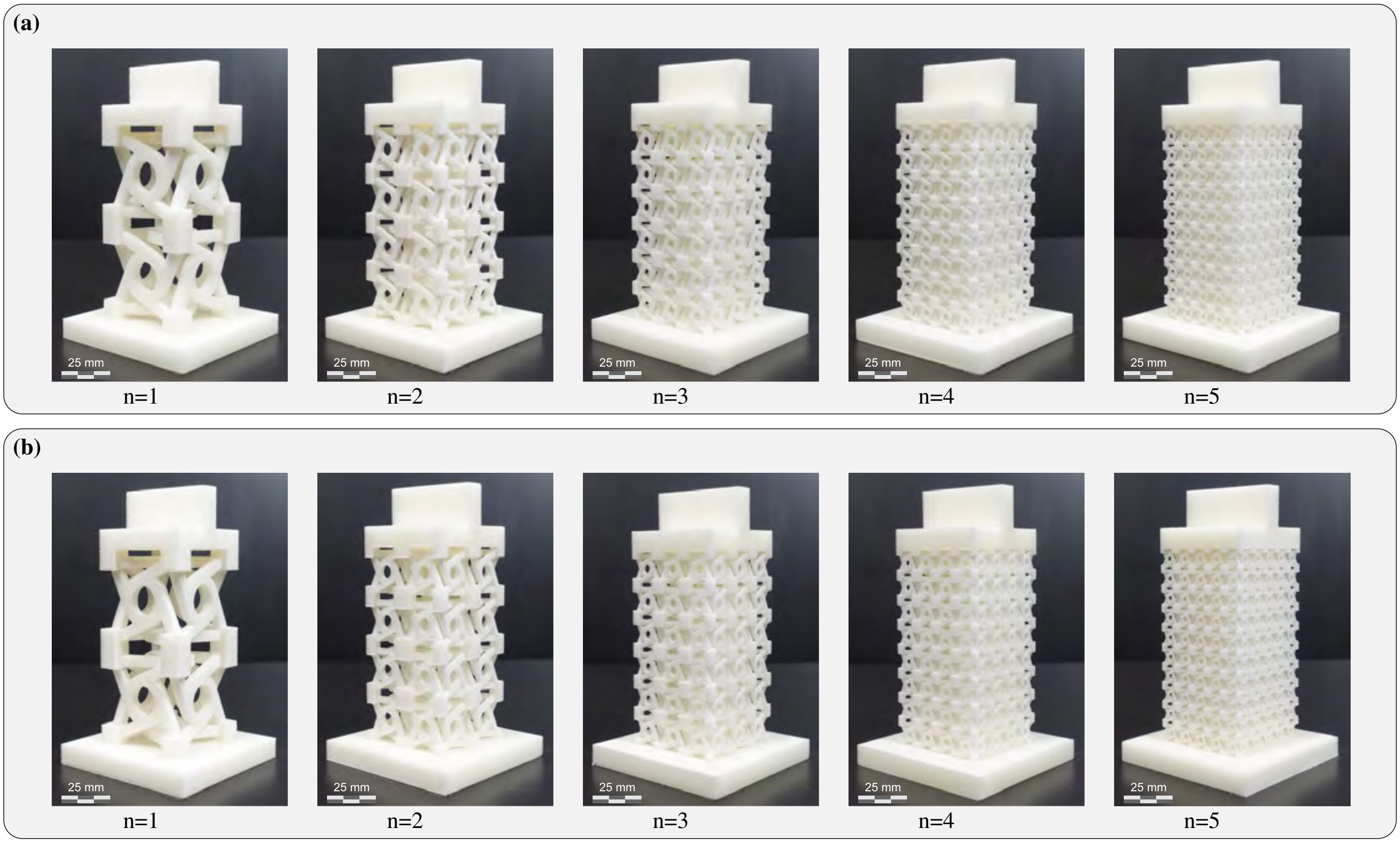

Figure S14: 3D printed (a) left-handed, and (b) right-handed chiral beam samples with $N = 2$ and $n = 1, 2, \cdots, 5$.

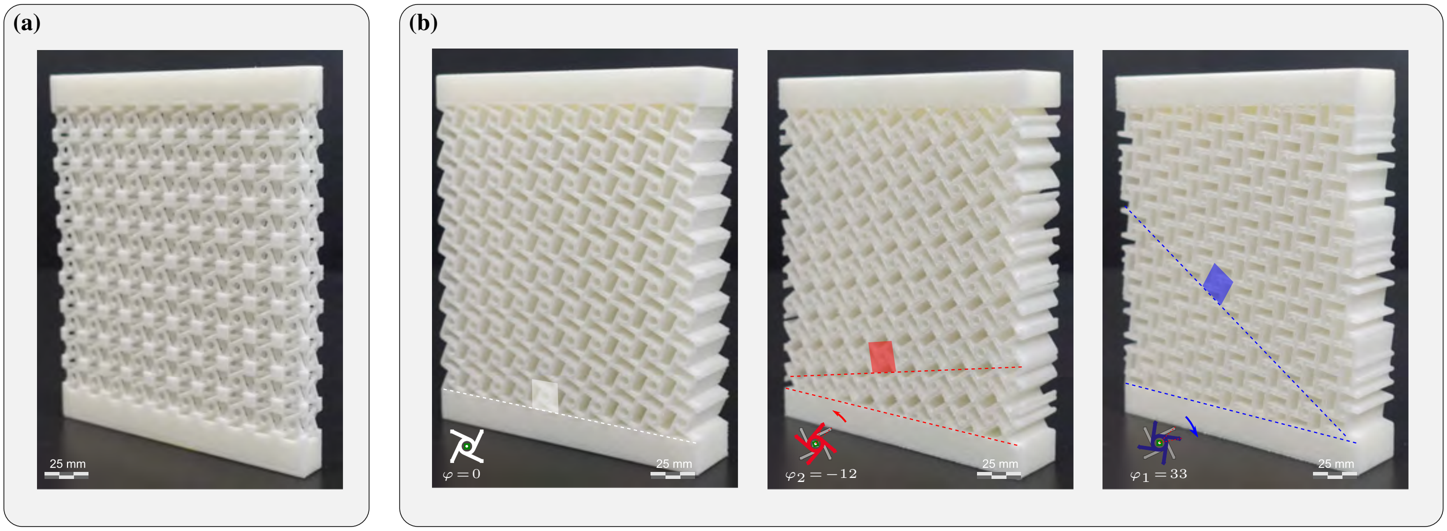

Figure S15: 3D printed plate samples (a) chiral plate made of 10×10 unit cells with side length of $1cm$, 2D-tetrachiral plates made of 10×10 unit cells with side length of $1cm$ and thickness of $2cm$ at different orientations (b) $\varphi = 0\,\mathrm{deg}$, (c) $\varphi = -12\,\mathrm{deg}$, and (d) tetra-chiral $\varphi = 33\,\mathrm{deg}$.

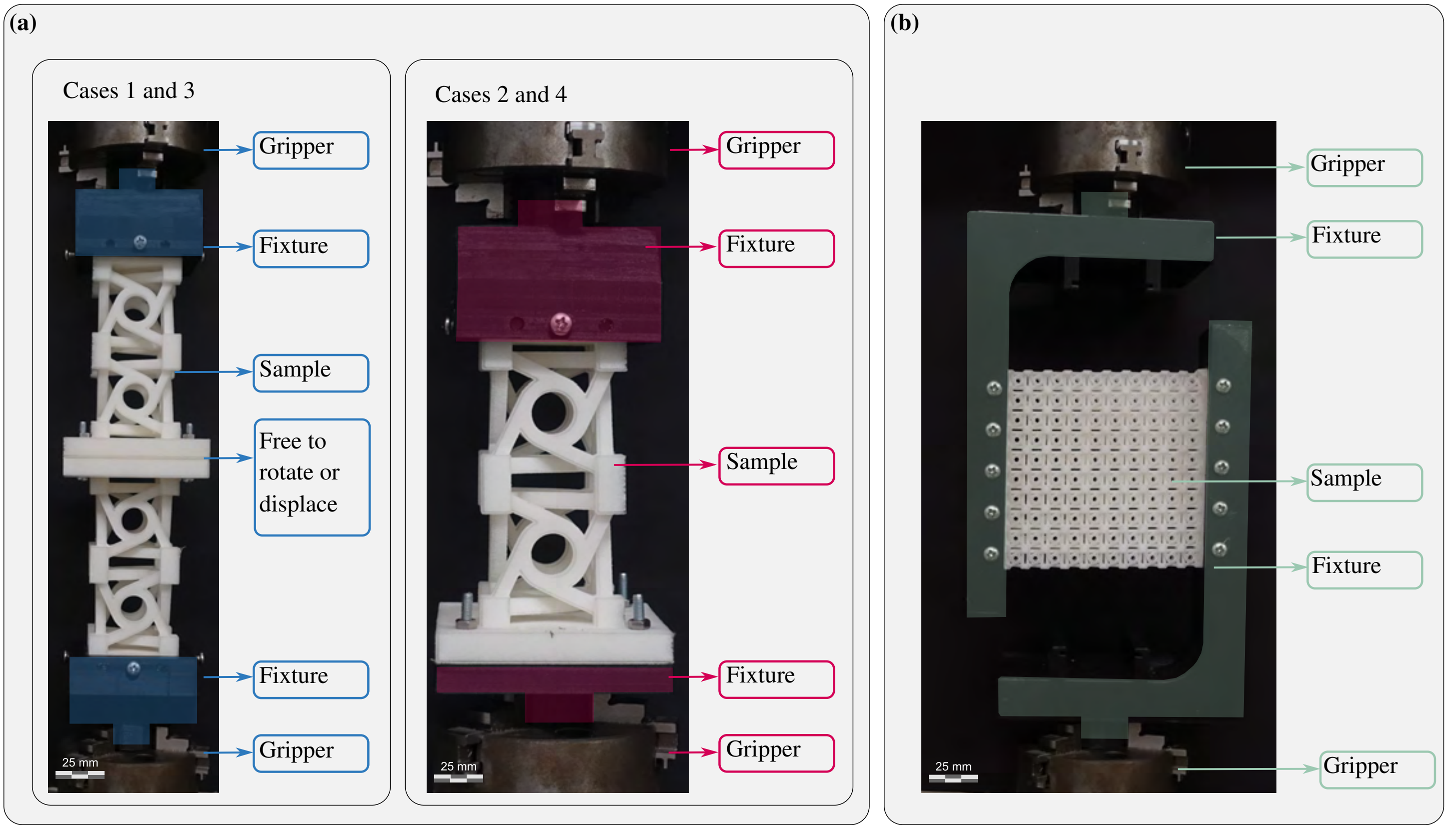

Figure S16: Experimental setup for (a) beam and (b) plate samples.

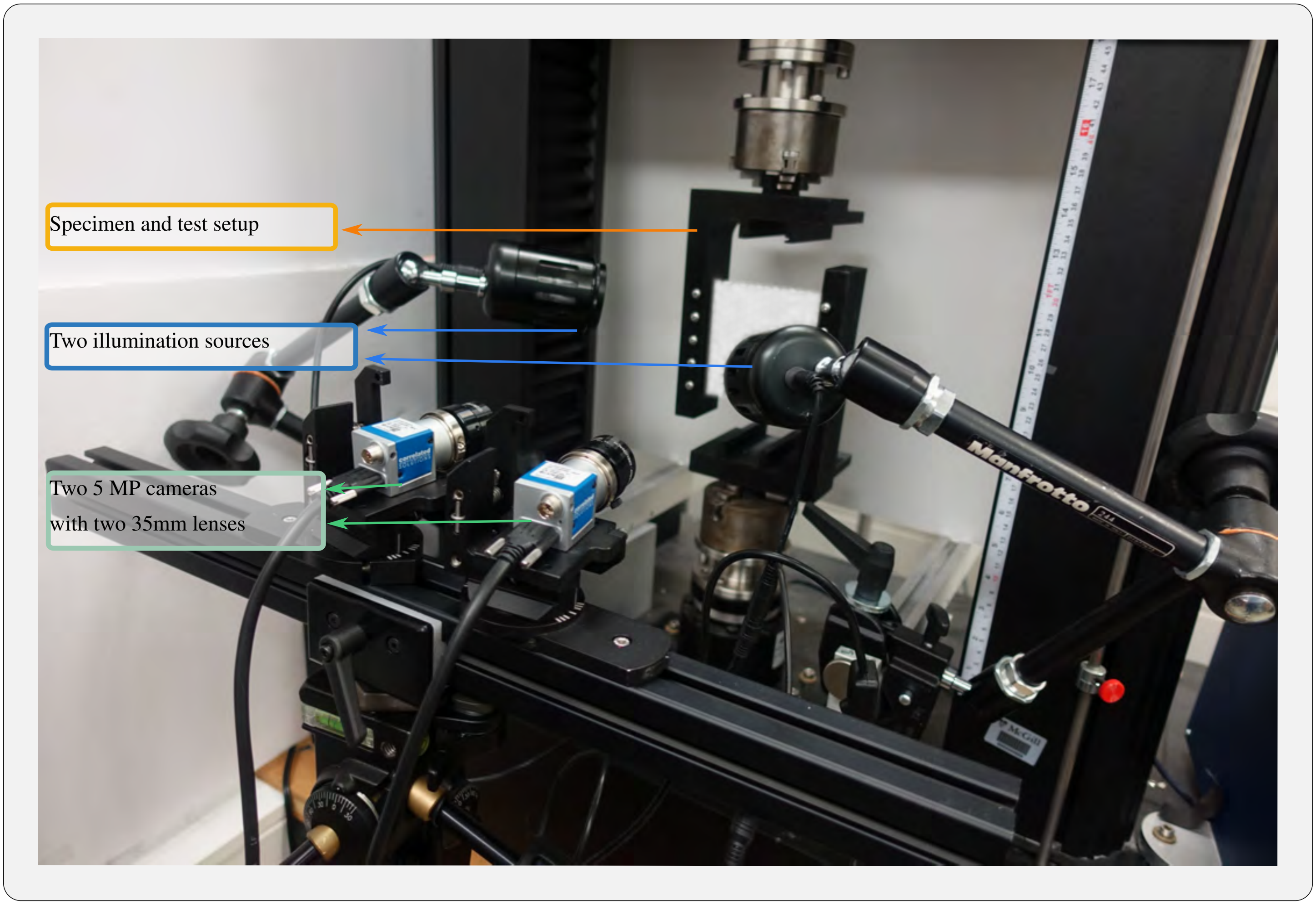

Figure S17: Test setup and positioning of the cameras, illuminating sources, and specimen.

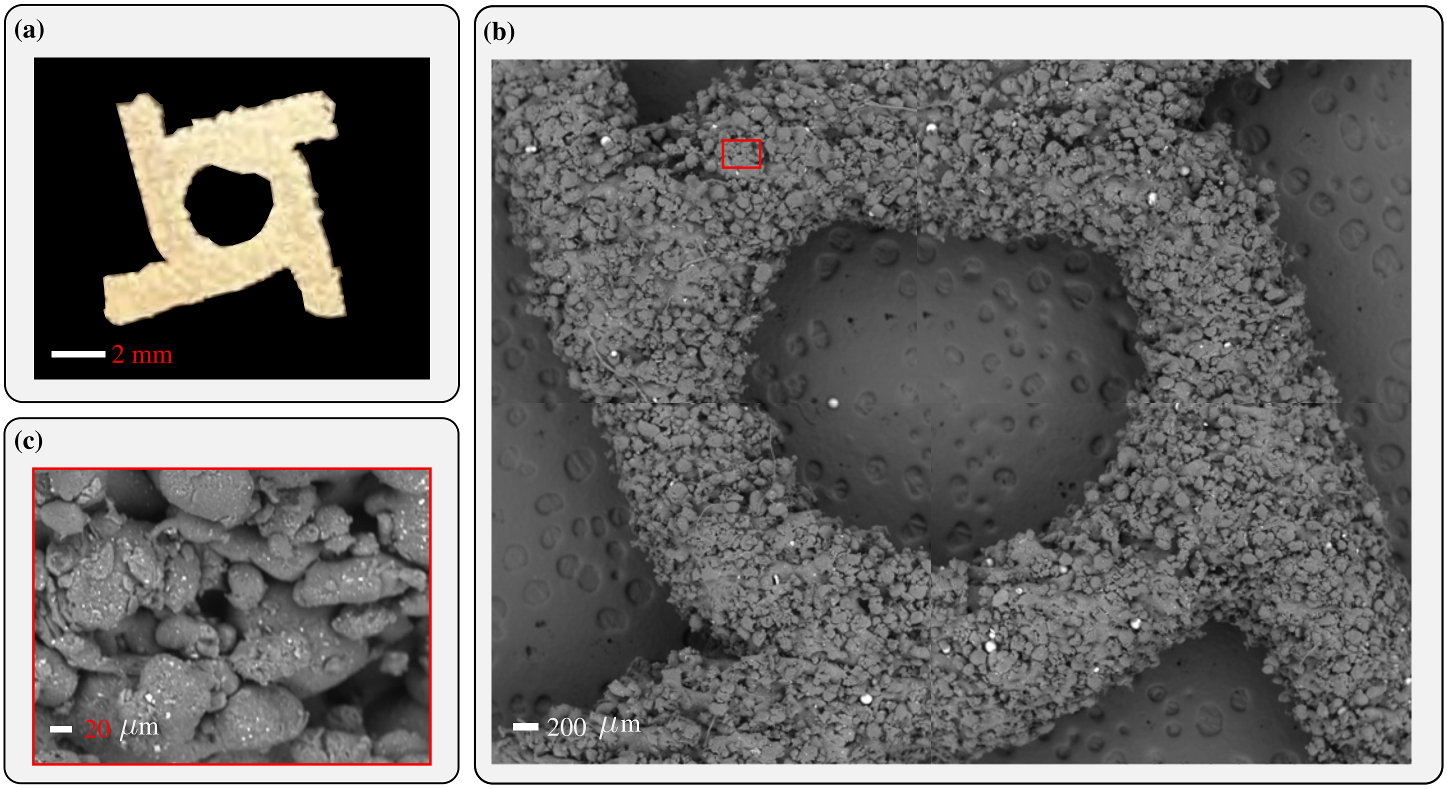

Figure S18: Defects in the 3D-printed sample. (a) chiral unit cell. (b) SEM imaging of the entire unit cell. (c) high-resolution SEM imaging, 10 times greater magnification than (b).

Supplementary Tables

Table S1: Thirty-two crystal classes with their Voigt class number, Schönflies and Hermann-Maugin notations, point, axes, and planes of symmetries.

Voigt	Schönflies	Hermann-Maugin		Point symmetry	Axes				Planes	Center
		Full	Short		2-Fold	3-Fold	4-Fold	6-Fold		
1	$C_i = S_2$	1	1	Central	-	-	-	-	-	yes
2	C_1	$\bar{1}$	$\bar{1}$	Chiral-Polar	-	-	-	-	-	-
3	C_{2h}	$\frac{2}{m}$	$2/m$	Central	1	-	-	-	1	yes
4	$C_S = C_{1h}$	m	m	Polar	-	-	-	-	1	-
5	C_2	2	2	Chiral-Polar	1	-	-	-	-	-
6	$D_{2h} = V_h$	$\frac{2}{m}\frac{2}{m}\frac{2}{m}$	mmm	Central	3	-	-	-	3	yes
7	$D_2 = V$	222	222	Chiral	3	-	-	-	-	-
8	C_{2v}	$mm2$	$mm2$	Polar	1	-	-	-	2	-
9	D_{3d}	$\bar{3}\frac{2}{m}$	$\bar{3}m$	Central	3	1	-	-	3	yes
10	D_3	32	32	Chiral	3	1	-	-	-	-
11	C_{3v}	$3m$	$3m$	Polar	-	1	-	-	3	-
12	$C_{3i} = S_6$	$\bar{3}$	$\bar{3}$	Central	-	1	-	-	-	yes
13	C_3	3	3	Chiral-Polar	-	1	-	-	-	-
14	D_{4h}	$\frac{4}{m}\frac{2}{m}\frac{2}{m}$	$4/mmm$	Central	4	-	1	-	5	yes
15	D_4	422	422	Chiral	4	-	1	-	-	-
16	C_{4v}	$4mm$	$4mm$	Polar	-	-	-	-	4	-
17	C_{4h}	$\frac{4}{m}$	$4/m$	Central	-	-	1	-	1	yes
18	C_4	4	4	Chiral-Polar	-	-	1	-	-	-
19	$D_{2d} = V_d$	$\bar{4}2m$	$\bar{4}2m$	Non-Central	3	-	-	-	2	-
20	S_4	$\bar{4}$	$\bar{4}$	Non-Central	1	-	-	-	-	-
21	D_{6h}	$\frac{6}{m}\frac{2}{m}\frac{2}{m}$	$6/mmm$	Central	6	-	-	1	7	yes
22	C_{6v}	$6mm$	$6mm$	Polar	-	-	-	1	6	-
23	D_6	622	622	Chiral	6	-	-	1	-	-
24	C_{6h}	$\frac{6}{m}$	$6/m$	Central	-	-	-	1	1	yes
25	C_6	6	6	Chiral-Polar	-	-	-	1	-	-
26	D_{3h}	$\bar{6}m2$	$\bar{6}m2$	Non-Central	3	1	-	-	4	-
27	C_{3h}	$\bar{6}$	$\bar{6}$	Non-Central	-	1	-	-	1	-
28	O_h	$\frac{4}{m}\bar{3}\frac{2}{m}$	$m\bar{3}m$	Central	6	4	3	-	9	yes
29	O	432	432	Chiral	6	4	3	-	-	-
30	T_d	$\bar{4}3m$	$\bar{4}3m$	Non-Central	3	4	-	-	6	-
31	T_h	$\frac{2}{m}\bar{3}$	$m\bar{3}$	Central	3	4	-	-	3	yes
32	T	23	23	Chiral	3	4	-	-	-	-

Table S2: Comparison of components of vectors and tensors in 3D, 2D and 1D spaces.

**Space**	**3D**	**2D**	**1D**
DF	$\hat{u}_1$, $\hat{u}_2$, $\hat{u}_3$	$\hat{u}_1$, $\hat{u}_2$, $\hat{u}_3$	$\hat{u}_1$, $\hat{u}_2$, $\hat{u}_3$, $\hat{\theta}_1$
$\mathbf{x}$	x_1, x_2, x_3	x_1, x_2	x_1
	$\bar{u}_1=\hat{u}_1+\hat{\theta}_2 y_3-\hat{\theta}_3 y_2$	$\bar{u}_1=\hat{u}_1+\hat{\theta}_2 y_3-\hat{\theta}_3 y_2$	$\bar{u}_1=\hat{u}_1+\hat{\theta}_2 y_3-\hat{\theta}_3 y_2$
$\bar{\mathbf{u}}$	$\bar{u}_2=\hat{u}_2-\hat{\theta}_1 y_3+\hat{\theta}_3 y_1$	$\bar{u}_2=\hat{u}_2-\hat{\theta}_1 y_3+\hat{\theta}_3 y_1$	$\bar{u}_2=\hat{u}_2 \qquad -\hat{\theta}_1 y_3$
	$\bar{u}_3=\hat{u}_3+\hat{\theta}_1 y_2-\hat{\theta}_2 y_1$	$\bar{u}_3=\hat{u}_3$	$\bar{u}_3=\hat{u}_3+\hat{\theta}_1 y_2$
	$\hat{\theta}_1=\frac{1}{2}\left(\hat{u}_{3,2}-\hat{u}_{2,3}\right)$	$\hat{\theta}_1=\hat{u}_{3,2}$	$\hat{\theta}_1=\hat{\theta}_1$
$\hat{\boldsymbol{\theta}}$	$\hat{\theta}_2=\frac{1}{2}\left(\hat{u}_{1,3}-\hat{u}_{3,1}\right)$	$\hat{\theta}_2=-\hat{u}_{3,1}$	$\hat{\theta}_2=-\hat{u}_{3,1}$
	$\hat{\theta}_3=\frac{1}{2}\left(\hat{u}_{2,1}-\hat{u}_{1,2}\right)$	$\hat{\theta}_3=\frac{1}{2}\left(\hat{u}_{2,1}-\hat{u}_{1,2}\right)$	$\hat{\theta}_3=\hat{u}_{2,1}$
	$\hat{\epsilon}_{11}=\hat{u}_{1,1}$	$\hat{\epsilon}_{11}=\hat{u}_{1,1}$	$\hat{\epsilon}_{11}=\hat{u}_{1,1}$
	$\hat{\epsilon}_{22}=\hat{u}_{2,2}$	$\hat{\epsilon}_{22}=\hat{u}_{2,2}$	$\hat{\epsilon}_{22}=0$
$\hat{\boldsymbol{\epsilon}}$	$\hat{\epsilon}_{33}=\hat{u}_{3,3}$	$\hat{\epsilon}_{33}=0$	$\hat{\epsilon}_{22}=0$
	$\hat{\epsilon}_{12}=\frac{1}{2}\left(\hat{u}_{1,2}+\hat{u}_{2,1}\right)$	$\hat{\epsilon}_{12}=\frac{1}{2}\left(\hat{u}_{1,2}+\hat{u}_{2,1}\right)$	$\hat{\epsilon}_{12}=0$
	$\hat{\epsilon}_{13}=\frac{1}{2}\left(\hat{u}_{1,3}+\hat{u}_{3,1}\right)$	$\hat{\epsilon}_{13}=0$	$\hat{\epsilon}_{13}=0$
	$\hat{\epsilon}_{23}=\frac{1}{2}\left(\hat{u}_{2,3}+\hat{u}_{3,2}\right)$	$\hat{\epsilon}_{23}=0$	$\hat{\epsilon}_{23}=0$
	$\hat{\kappa}_{11}=\frac{1}{2}\left(\hat{u}_{3,12}-\hat{u}_{2,13}\right)$	$\hat{\kappa}_{11}=\hat{u}_{3,12}$	$\hat{\kappa}_{11}=\hat{\theta}_{1,1}$
	$\hat{\kappa}_{12}=\frac{1}{2}\left(\hat{u}_{3,22}-\hat{u}_{2,23}\right)$	$\hat{\kappa}_{12}=\hat{u}_{3,22}$	$\hat{\kappa}_{12}=0$
	$\hat{\kappa}_{13}=\frac{1}{2}\left(\hat{u}_{3,32}-\hat{u}_{2,33}\right)$	$\hat{\kappa}_{13}=0$	$\hat{\kappa}_{13}=0$
	$\hat{\kappa}_{21}=\frac{1}{2}\left(\hat{u}_{1,13}-\hat{u}_{3,11}\right)$	$\hat{\kappa}_{21}=-\hat{u}_{3,11}$	$\hat{\kappa}_{21}=-\hat{u}_{3,11}$
$\hat{\boldsymbol{\kappa}}$	$\hat{\kappa}_{22}=\frac{1}{2}\left(\hat{u}_{1,23}-\hat{u}_{3,21}\right)$	$\hat{\kappa}_{22}=-\hat{u}_{3,21}$	$\hat{\kappa}_{22}=0$
	$\hat{\kappa}_{23}=\frac{1}{2}\left(\hat{u}_{1,33}-\hat{u}_{3,31}\right)$	$\hat{\kappa}_{23}=0$	$\hat{\kappa}_{23}=0$
	$\hat{\kappa}_{31}=\frac{1}{2}\left(\hat{u}_{2,11}-\hat{u}_{1,12}\right)$	$\hat{\kappa}_{31}=\frac{1}{2}\left(\hat{u}_{2,11}-\hat{u}_{1,12}\right)$	$\hat{\kappa}_{31}=\hat{u}_{2,11}$
	$\hat{\kappa}_{32}=\frac{1}{2}\left(\hat{u}_{2,21}-\hat{u}_{1,22}\right)$	$\hat{\kappa}_{32}=\frac{1}{2}\left(\hat{u}_{2,21}-\hat{u}_{1,22}\right)$	$\hat{\kappa}_{32}=0$
	$\hat{\kappa}_{33}=\frac{1}{2}\left(\hat{u}_{2,31}-\hat{u}_{1,32}\right)$	$\hat{\kappa}_{33}=0$	$\hat{\kappa}_{33}=0$
	$\bar{\epsilon}_{11}=\hat{\epsilon}_{11}+\left(\hat{\kappa}_{21}y_3-\hat{\kappa}_{31}y_2\right)$	$\bar{\epsilon}_{11}=\hat{\epsilon}_{11}+\left(\hat{\kappa}_{21}y_3-\hat{\kappa}_{31}y_2\right)$	$\bar{\epsilon}_{11}=\hat{\epsilon}_{11}+\left(\hat{\kappa}_{21}y_3-\hat{\kappa}_{31}y_2\right)$
	$\bar{\epsilon}_{22}=\hat{\epsilon}_{22}+\left(\hat{\kappa}_{32}y_1-\hat{\kappa}_{12}y_3\right)$	$\bar{\epsilon}_{22}=\hat{\epsilon}_{22}+\left(\hat{\kappa}_{32}y_1-\hat{\kappa}_{12}y_3\right)$	$\bar{\epsilon}_{22}=0$
$\bar{\boldsymbol{\epsilon}}$	$\bar{\epsilon}_{33}=\hat{\epsilon}_{33}+\left(\hat{\kappa}_{13}y_2-\hat{\kappa}_{23}y_1\right)$	$\bar{\epsilon}_{33}=0$	$\bar{\epsilon}_{33}=0$
	$\bar{\epsilon}_{12}=\hat{\epsilon}_{12}+\frac{1}{2}\left(\hat{\kappa}_{31}y_1-\hat{\kappa}_{32}y_2+\left(\hat{\kappa}_{22}-\hat{\kappa}_{11}\right)y_3\right)$	$\bar{\epsilon}_{12}=\hat{\epsilon}_{12}+\frac{1}{2}\left(\hat{\kappa}_{31}y_1-\hat{\kappa}_{32}y_2+\left(\hat{\kappa}_{22}-\hat{\kappa}_{11}\right)y_3\right)$	$\bar{\epsilon}_{12}=+\frac{1}{2}\left(-\hat{\kappa}_{11}y_3\right)$
	$\bar{\epsilon}_{13}=\hat{\epsilon}_{13}+\frac{1}{2}\left(\hat{\kappa}_{23}y_3-\hat{\kappa}_{21}y_1+\left(\hat{\kappa}_{11}-\hat{\kappa}_{33}\right)y_2\right)$	$\bar{\epsilon}_{13}=0$	$\bar{\epsilon}_{13}=+\frac{1}{2}\left(\hat{\kappa}_{11}y_2\right)$
	$\bar{\epsilon}_{23}=\hat{\epsilon}_{23}+\frac{1}{2}\left(\hat{\kappa}_{12}y_2-\hat{\kappa}_{13}y_3+\left(\hat{\kappa}_{33}-\hat{\kappa}_{22}\right)y_1\right)$	$\bar{\epsilon}_{23}=0$	$\bar{\epsilon}_{23}=0$

Continued on next page

Table S2 – continued from previous page

Space	3D	2D	1D
	$\hat{\sigma}_{11}=\langle\bar{\sigma}_{11}+\sigma^*_{11}\rangle_{\mathrm{Y}}$	$\hat{\sigma}_{11}=\langle\bar{\sigma}_{11}+\sigma^*_{11}\rangle_{\mathrm{Y}}$	$\hat{\sigma}_{11}=\langle\bar{\sigma}_{11}+\sigma^*_{11}\rangle_{\mathrm{Y}}$
	$\hat{\sigma}_{22}=\langle\bar{\sigma}_{22}+\sigma^*_{22}\rangle_{\mathrm{Y}}$	$\hat{\sigma}_{22}=\langle\bar{\sigma}_{22}+\sigma^*_{22}\rangle_{\mathrm{Y}}$	$\hat{\sigma}_{22}=0$
$\hat{\boldsymbol{\sigma}}$	$\hat{\sigma}_{33}=\langle\bar{\sigma}_{33}+\sigma^*_{33}\rangle_{\mathrm{Y}}$	$\hat{\sigma}_{33}=0$	$\hat{\sigma}_{33}=0$
	$\hat{\sigma}_{12}=\langle\bar{\sigma}_{12}+\sigma^*_{12}\rangle_{\mathrm{Y}}$	$\hat{\sigma}_{12}=\langle\bar{\sigma}_{12}+\sigma^*_{12}\rangle_{\mathrm{Y}}$	$\hat{\sigma}_{12}=0$
	$\hat{\sigma}_{13}=\langle\bar{\sigma}_{13}+\sigma^*_{13}\rangle_{\mathrm{Y}}$	$\hat{\sigma}_{13}=0$	$\hat{\sigma}_{13}=0$
	$\hat{\sigma}_{23}=\langle\bar{\sigma}_{23}+\sigma^*_{23}\rangle_{\mathrm{Y}}$	$\hat{\sigma}_{23}=0$	$\hat{\sigma}_{23}=0$
	$\hat{\mu}_{11}=\langle(\bar{\sigma}_{13}+\sigma^*_{13})\,y_2-(\bar{\sigma}_{12}+\sigma^*_{12})\,y_3\rangle_{\mathrm{Y}}$	$\hat{\mu}_{11}=\langle\quad-(\bar{\sigma}_{12}+\sigma^*_{12})\,y_3\rangle_{\mathrm{Y}}$	$\hat{\mu}_{11}=\langle(\bar{\sigma}_{13}+\sigma^*_{13})\,y_2-(\bar{\sigma}_{12}+\sigma^*_{12})\,y_3\rangle_{\mathrm{Y}}$
	$\hat{\mu}_{22}=\langle(\bar{\sigma}_{12}+\sigma^*_{12})\,y_3-(\bar{\sigma}_{23}+\sigma^*_{23})\,y_1\rangle_{\mathrm{Y}}$	$\hat{\mu}_{22}=\langle(\bar{\sigma}_{12}+\sigma^*_{12})\,y_3\quad\rangle_{\mathrm{Y}}$	$\hat{\mu}_{22}=0$
	$\hat{\mu}_{33}=\langle(\bar{\sigma}_{23}+\sigma^*_{23})\,y_1-(\bar{\sigma}_{13}+\sigma^*_{13})\,y_2\rangle_{\mathrm{Y}}$	$\hat{\mu}_{33}=0$	$\hat{\mu}_{33}=0$
	$\hat{\mu}_{12}=\langle(\bar{\sigma}_{23}+\sigma^*_{23})\,y_2-(\bar{\sigma}_{22}+\sigma^*_{22})\,y_3\rangle_{\mathrm{Y}}$	$\hat{\mu}_{12}=\langle\quad-(\bar{\sigma}_{22}+\sigma^*_{22})\,y_3\rangle_{\mathrm{Y}}$	$\hat{\mu}_{12}=0$
$\hat{\boldsymbol{\mu}}$	$\hat{\mu}_{21}=\langle(\bar{\sigma}_{11}+\sigma^*_{11})\,y_3-(\bar{\sigma}_{13}+\sigma^*_{13})\,y_1\rangle_{\mathrm{Y}}$	$\hat{\mu}_{21}=\langle(\bar{\sigma}_{11}+\sigma^*_{11})\,y_3\quad\rangle_{\mathrm{Y}}$	$\hat{\mu}_{21}=\langle(\bar{\sigma}_{11}+\sigma^*_{11})\,y_3\quad\rangle_{\mathrm{Y}}$
	$\hat{\mu}_{13}=\langle(\bar{\sigma}_{33}+\sigma^*_{33})\,y_2-(\bar{\sigma}_{23}+\sigma^*_{23})\,y_3\rangle_{\mathrm{Y}}$	$\hat{\mu}_{13}=0$	$\hat{\mu}_{13}=0$
	$\hat{\mu}_{31}=\langle(\bar{\sigma}_{12}+\sigma^*_{12})\,y_1-(\bar{\sigma}_{11}+\sigma^*_{11})\,y_2\rangle_{\mathrm{Y}}$	$\hat{\mu}_{31}=\langle(\bar{\sigma}_{12}+\sigma^*_{12})\,y_1-(\bar{\sigma}_{11}+\sigma^*_{11})\,y_2\rangle_{\mathrm{Y}}$	$\hat{\mu}_{31}=\langle\quad-(\bar{\sigma}_{11}+\sigma^*_{11})\,y_2\rangle_{\mathrm{Y}}$
	$\hat{\mu}_{23}=\langle(\bar{\sigma}_{13}+\sigma^*_{13})\,y_3-(\bar{\sigma}_{33}+\sigma^*_{33})\,y_1\rangle_{\mathrm{Y}}$	$\hat{\mu}_{23}=0$	$\hat{\mu}_{23}=0$
	$\hat{\mu}_{32}=\langle(\bar{\sigma}_{22}+\sigma^*_{22})\,y_1-(\bar{\sigma}_{12}+\sigma^*_{12})\,y_2\rangle_{\mathrm{Y}}$	$\hat{\mu}_{32}=\langle(\bar{\sigma}_{22}+\sigma^*_{22})\,y_1-(\bar{\sigma}_{12}+\sigma^*_{12})\,y_2\rangle_{\mathrm{Y}}$	$\hat{\mu}_{32}=0$
	$\hat{S}_{11}=\hat{\sigma}_{11}$	$\hat{S}_{11}=\hat{\sigma}_{11}$	$\hat{S}_{11}=\hat{\sigma}_{11}$
	$\hat{S}_{22}=\hat{\sigma}_{22}$	$\hat{S}_{22}=\hat{\sigma}_{22}$	$\hat{S}_{22}=0$
	$\hat{S}_{33}=\hat{\sigma}_{33}$	$\hat{S}_{33}=0$	$\hat{S}_{33}=0$
	$\hat{S}_{12}=\hat{\sigma}_{12}-\hat{\tau}_3$	$\hat{S}_{12}=\hat{\sigma}_{12}-\hat{\tau}_3$	$\hat{S}_{12}=0$
$\hat{\mathbf{S}}$	$\hat{S}_{21}=\hat{\sigma}_{12}+\hat{\tau}_3$	$\hat{S}_{21}=\hat{\sigma}_{12}+\hat{\tau}_3$	$\hat{S}_{21}=\hat{\tau}_3$
	$\hat{S}_{13}=\hat{\sigma}_{13}+\hat{\tau}_2$	$\hat{S}_{13}=0$	$\hat{S}_{13}=0$
	$\hat{S}_{31}=\hat{\sigma}_{13}-\hat{\tau}_2$	$\hat{S}_{31}=-\hat{\tau}_2$	$\hat{S}_{31}=-\hat{\tau}_2$
	$\hat{S}_{23}=\hat{\sigma}_{23}-\hat{\tau}_1$	$\hat{S}_{23}=0$	$\hat{S}_{23}=0$
	$\hat{S}_{32}=\hat{\sigma}_{23}+\hat{\tau}_1$	$\hat{S}_{32}=\hat{\tau}_1$	$\hat{S}_{32}=0$
	$\hat{\sigma}_{11}=\hat{S}_{11}$	$\hat{\sigma}_{11}=\hat{S}_{11}$	$\hat{\sigma}_{11}=\hat{S}_{11}$
	$\hat{\sigma}_{22}=\hat{S}_{22}$	$\hat{\sigma}_{22}=\hat{S}_{22}$	$\hat{\sigma}_{22}=0$
$\hat{\boldsymbol{\sigma}}$	$\hat{\sigma}_{33}=\hat{S}_{33}$	$\hat{\sigma}_{33}=0$	$\hat{\sigma}_{33}=0$
	$\hat{\sigma}_{12}=\frac{1}{2}\left(\hat{S}_{12}+\hat{S}_{21}\right)$	$\hat{\sigma}_{12}=\frac{1}{2}\left(\hat{S}_{12}+\hat{S}_{21}\right)$	$\hat{\sigma}_{12}=0$
	$\hat{\sigma}_{13}=\frac{1}{2}\left(\hat{S}_{13}+\hat{S}_{31}\right)$	$\hat{\sigma}_{13}=0$	$\hat{\sigma}_{13}=0$
	$\hat{\sigma}_{23}=\frac{1}{2}\left(\hat{S}_{23}+\hat{S}_{32}\right)$	$\hat{\sigma}_{23}=0$	$\hat{\sigma}_{23}=0$

Continued on next page

Table S2 – continued from previous page

Space	3D	2D	1D
	$\hat{\tau}_1 = \frac{1}{2}\left(\hat{S}_{32} - \hat{S}_{23}\right)$	$\hat{\tau}_1 = \hat{S}_{32}$	$\hat{\tau}_1 = 0$
$\hat{\boldsymbol{\tau}}$	$\hat{\tau}_2 = \frac{1}{2}\left(\hat{S}_{13} - \hat{S}_{31}\right)$	$\hat{\tau}_2 = -\hat{S}_{31}$	$\hat{\tau}_2 = -\hat{S}_{31}$
	$\hat{\tau}_3 = \frac{1}{2}\left(\hat{S}_{21} - \hat{S}_{12}\right)$	$\hat{\tau}_3 = \frac{1}{2}\left(\hat{S}_{21} - \hat{S}_{12}\right)$	$\hat{\tau}_3 = \hat{S}_{21}$

Table S3: Comparison of conservation laws in 3D, 2D and 1D spaces.

Space	3D	2D	1D
Conservation of Linear Momentum (force-stress equilibrium)	$\hat{S}_{11,1} + \hat{S}_{12,2} + \hat{S}_{13,3} + \hat{b}_1 = \dot{\hat{L}}_1$<br>$\hat{S}_{21,1} + \hat{S}_{22,2} + \hat{S}_{23,3} + \hat{b}_2 = \dot{\hat{L}}_2$<br>$\hat{S}_{31,1} + \hat{S}_{32,2} + \hat{S}_{33,3} + \hat{b}_3 = \dot{\hat{L}}_3$	$\hat{S}_{11,1} + \hat{S}_{12,2} + \hat{b}_1 = \dot{\hat{L}}_1$<br>$\hat{S}_{21,1} + \hat{S}_{22,2} + \hat{b}_2 = \dot{\hat{L}}_2$<br>$\hat{S}_{31,1} + \hat{S}_{32,2} + \hat{b}_3 = \dot{\hat{L}}_3$	$\hat{S}_{11,1} + \hat{b}_1 = \dot{\hat{L}}_1$<br>$\hat{S}_{21,1} + \hat{b}_2 = \dot{\hat{L}}_2$<br>$\hat{S}_{31,1} + \hat{b}_3 = \dot{\hat{L}}_3$
Conservation of Angular Momentum (Couple-stress equilibrium)	$\hat{\mu}_{11,1} + \hat{\mu}_{12,2} + \hat{\mu}_{13,3} + \hat{c}_1 + 2\hat{\tau}_1 = \dot{\hat{J}}_1$<br>$\hat{\mu}_{21,1} + \hat{\mu}_{22,2} + \hat{\mu}_{23,3} + \hat{c}_2 + 2\hat{\tau}_2 = \dot{\hat{J}}_2$<br>$\hat{\mu}_{31,1} + \hat{\mu}_{32,2} + \hat{\mu}_{33,3} + \hat{c}_3 + 2\hat{\tau}_3 = \dot{\hat{J}}_3$	$\hat{\mu}_{11,1} + \hat{\mu}_{12,2} + \hat{c}_1 + \hat{\tau}_1 = \dot{\hat{J}}_1$<br>$\hat{\mu}_{21,1} + \hat{\mu}_{22,2} + \hat{c}_2 + \hat{\tau}_2 = \dot{\hat{J}}_2$<br>$\hat{\mu}_{31,1} + \hat{\mu}_{32,2} + \hat{c}_3 + 2\hat{\tau}_3 = \dot{\hat{J}}_3$	$\hat{\mu}_{11,1} + \hat{c}_1 = \dot{\hat{J}}_1$<br>$\hat{\mu}_{21,1} + \hat{c}_2 + \hat{\tau}_2 = \dot{\hat{J}}_2$<br>$\hat{\mu}_{31,1} + \hat{c}_3 + \hat{\tau}_3 = \dot{\hat{J}}_3$

Table S4: Comparison of governing in 3D, 2D and 1D spaces.

3D	$\hat{\sigma}_{11,1} + \hat{\sigma}_{12,2} + \hat{\sigma}_{13,3} + \frac{1}{2}\left[(\hat{\mu}_{31,1} + \hat{\mu}_{32,2} + \hat{\mu}_{33,3})_{,2} - (\hat{\mu}_{21,1} + \hat{\mu}_{22,2} + \hat{\mu}_{23,3})_{,3}\right] + \hat{b}_1 + \frac{1}{2}(\hat{c}_{3,2} - \hat{c}_{2,3}) = \dot{\hat{L}}_1 + \frac{1}{2}(\dot{\hat{J}}_{3,2} - \dot{\hat{J}}_{2,3})$
	$\hat{\sigma}_{21,1} + \hat{\sigma}_{22,2} + \hat{\sigma}_{23,3} + \frac{1}{2}\left[(\hat{\mu}_{11,1} + \hat{\mu}_{12,2} + \hat{\mu}_{13,3})_{,3} - (\hat{\mu}_{31,1} + \hat{\mu}_{32,2} + \hat{\mu}_{33,3})_{,1}\right] + \hat{b}_2 + \frac{1}{2}(\hat{c}_{1,3} - \hat{c}_{3,1}) = \dot{\hat{L}}_2 + \frac{1}{2}(\dot{\hat{J}}_{1,3} - \dot{\hat{J}}_{3,1})$
	$\hat{\sigma}_{31,1} + \hat{\sigma}_{32,2} + \hat{\sigma}_{33,3} + \frac{1}{2}\left[(\hat{\mu}_{21,1} + \hat{\mu}_{22,2} + \hat{\mu}_{23,3})_{,1} - (\hat{\mu}_{11,1} + \hat{\mu}_{12,2} + \hat{\mu}_{13,3})_{,2}\right] + \hat{b}_3 + \frac{1}{2}(\hat{c}_{2,1} - \hat{c}_{1,2}) = \dot{\hat{L}}_3 + \frac{1}{2}(\dot{\hat{J}}_{2,1} - \dot{\hat{J}}_{1,2})$
2D	$\hat{\sigma}_{11,1} + \hat{\sigma}_{12,2} + \frac{1}{2}\left[(\hat{\mu}_{31,1} + \hat{\mu}_{32,2})_{,2}\right] + \hat{b}_1 + \frac{1}{2}(\hat{c}_{3,2}) = \dot{\hat{L}}_1 + \frac{1}{2}(\dot{\hat{J}}_{3,2})$
	$\hat{\sigma}_{21,1} + \hat{\sigma}_{22,2} + \frac{1}{2}\left[- (\hat{\mu}_{31,1} + \hat{\mu}_{32,2})_{,1}\right] + \hat{b}_2 + \frac{1}{2}(- \hat{c}_{3,1}) = \dot{\hat{L}}_2 + \frac{1}{2}(- \dot{\hat{J}}_{3,1})$
	$+ \left[(\hat{\mu}_{21,1} + \hat{\mu}_{22,2})_{,1} - (\hat{\mu}_{11,1} + \hat{\mu}_{12,2})_{,2}\right] + \hat{b}_3 + (\hat{c}_{2,1} - \hat{c}_{1,2}) = \dot{\hat{L}}_3 + (\dot{\hat{J}}_{2,1} - \dot{\hat{J}}_{1,2})$
1D	$\hat{\sigma}_{11,1} + \hat{b}_1 = \dot{\hat{L}}_1$
	$\left[- (\hat{\mu}_{31,1})_{,1}\right] + \hat{b}_2 + (- \hat{c}_{3,1}) = \dot{\hat{L}}_2 + (- \dot{\hat{J}}_{3,1})$
	$\left[(\hat{\mu}_{21,1})_{,1}\right] + \hat{b}_3 + (\hat{c}_{2,1}) = \dot{\hat{L}}_3 + (\dot{\hat{J}}_{2,1})$
	$\hat{\mu}_{11,1} + \hat{c}_1 = \dot{\hat{J}}_1$

Table S5: Effective CSBT material properties for each value of $n = 1, 2, \cdots, 15$.

	$n = th/\ell$	1	2	3	4	5	6	7	8	9	10	11	12	13	14	15	∞
$\not\propto th$	$E^{1D}(\%E^0)$	1.411	1.761	1.887	1.948	1.984	2.007	2.024	2.031	2.044	2.050	2.059	2.060	2.066	2.070	2.074	2.116
	$\rho^{1D}(\%\rho^0)$	20.998															
$\propto th$	$\beta^{1D}_{a\text{-}t}(\%E^0 th)$	−0.124	−0.089	−0.071	−0.057	−0.048	−0.041	−0.036	−0.032	−0.028	−0.026	−0.024	−0.022	−0.020	−0.019	−0.017	0.000
$\propto th^2$	$I^{1D}(\%E^0 th^2)$	0.154	0.145	0.150	0.154	0.158	0.160	0.163	0.164	0.165	0.166	0.167	0.168	0.169	0.169	0.170	0.176
	$\eta^{1D}_t(\%E^0 th^2)$	0.166	0.157	0.152	0.150	0.148	0.147	0.146	0.146	0.146	0.145	0.145	0.145	0.145	0.145	0.145	0.145
	$\iota^{1D}_{11}(\%\rho^0 th^2)$	4.303	3.700	3.589	3.550	3.532	3.522	3.516	3.512	3.510	3.508	3.506	3.505	3.504	3.504	3.503	3.500
	$\iota^{1D}_{22}(\%\rho^0 th^2)$	4.303	2.388	2.034	1.909	1.852	1.821	1.802	1.790	1.781	1.775	1.771	1.768	1.765	1.763	1.761	1.750

Table S6: Effective CSPT material properties for each value of $n = 1, 2, \cdots, 15$.

	n	1	2	3	4	5	6	7	8	9	10	11	12	13	14	15	∞
$\not\propto th$	$K^{2D}(\%E^0)$	0.908	1.038	1.093	1.121	1.138	1.149	1.157	1.163	1.167	1.171	1.175	1.178	1.180	1.181	1.183	1.205
	$G_a^{2D}(\%E^0)$	0.906	0.925	0.931	0.934	0.935	0.936	0.937	0.938	0.938	0.939	0.939	0.940	0.940	0.940	0.941	0.943
	$G_s^{2D}(\%E^0)$	1.026	1.026	1.027	1.027	1.027	1.027	1.027	1.027	1.027	1.027	1.027	1.027	1.027	1.027	1.027	1.027
	$\rho^{2D}(\%\rho^0)$	20.998															
$\propto th$	$\beta_{a\text{-}t}^{2D}(\%E^0 th)$	−0.124	−0.061	−0.040	−0.030	−0.024	−0.020	−0.017	−0.015	−0.013	−0.012	−0.011	−0.010	−0.009	−0.009	−0.008	0.000
	$\beta_{s\text{-}b}^{2D}(\%E^0 th)$	0.154	0.077	0.051	0.038	0.031	0.025	0.022	0.019	0.017	0.015	0.014	0.013	0.012	0.011	0.010	0.000
$\propto th^2$	$\eta_t^{2D}(\%E^0 th^2)$	0.155	0.103	0.093	0.090	0.088	0.087	0.087	0.087	0.086	0.086	0.086	0.086	0.086	0.086	0.086	0.086
	$\eta_b^{2D}(\%E^0 th^2)$	0.093	0.081	0.079	0.078	0.078	0.078	0.078	0.078	0.078	0.078	0.078	0.078	0.078	0.078	0.078	0.078
	$\gamma^{2D}(\%E^0 th^2)$	0.098	0.081	0.083	0.085	0.087	0.089	0.090	0.091	0.092	0.093	0.093	0.094	0.094	0.095	0.095	0.100
	$I_3^{2D}(\%E^0 th^2)$	0.537	0.137	0.061	0.035	0.022	0.015	0.011	0.009	0.007	0.006	0.005	0.004	0.003	0.003	0.002	0.000
	$\iota_{11}^{2D}(\%\rho^0 th^2)$	4.303	2.388	2.034	1.909	1.852	1.821	1.802	1.790	1.781	1.775	1.771	1.768	1.765	1.763	1.761	1.750
	$\iota_{33}^{2D}(\%\rho^0 th^2)$	4.303	1.076	0.478	0.269	0.172	0.120	0.088	0.067	0.053	0.043	0.036	0.030	0.025	0.022	0.019	0.000

Table S7: The geometrical parameters and weight of the metabeams for both the as-fabricated and as-designed states.

n	Chirality	L_f (cm)	L_d (cm)	t_f (mm)	t_d (mm)	W_f (g)	W_d (g)
1	LH	9.95	10.00	5.41	5.00	155	162
1	RH	9.95	10.00	5.33	5.00	153	162
2	LH	9.90	10.00	2.97	2.50	163	162
2	RH	9.90	10.00	2.94	2.50	159	162
3	LH	9.85	10.00	2.15	1.67	175	162
3	RH	9.85	10.00	2.01	1.67	178	162
4	LH	9.95	10.00	1.73	1.25	195	162
4	RH	9.95	10.00	1.72	1.25	188	162
5	LH	9.80	10.00	1.42	1.00	207	162
5	RH	9.80	10.00	1.42	1.00	199	162

Table S8: The geometrical parameters and weight of the metaplates for both the as-fabricated and as-designed states.

Sample	L_f (cm)	L_d (cm)	t_f (mm)	t_d (mm)	th_f (mm)	th_d (mm)	W_f (g)	W_d (g)
Chiral $n = 1$, $N = 10$	9.90	10.00	1.42	1.00	9.93	10.00	51	45
Tetra-chiral $\varphi^D = 0$	9.85	10.00	1.45	1.00	19.93	20.00	134	117
Tetra-chiral $\varphi^D = 33$	10.39	10.00	1.45	1.00	20.02	20.00	124	117
Tetra-chiral $\varphi^D = -12$	10.19	10.00	1.35	1.00	20.15	20.00	123	117

Table S9: Comparison of the Double Curvature Ratio for 2D-Tetrachiral Metaplates: A Contrast between 2D-AAH and Detailed FEM Simulations under Free Boundary Conditions, and between Experimental and Simulational Data under Fixed Boundary Conditions.

	**Free-Boundary Conditions**			**Fixed-Boundary Conditions**		
Orientation	Theory	Simulation	Error (%)	Experiment	Simulation	Error (%)
$\varphi^D = 33^o$	-0.31	-0.335	8.0%	-0.217	-0.210	3.2%
$\varphi^D = -12^o$	0.71	0.755	6.3%	0.200	0.215	7.5%

Supplementary Movies

Movie S1. Case 1 - Induced twisting curvature due to application of axial strain (n=1)

Movie S2. Case 1 - Induced twisting curvature due to application of axial strain (n=2)

Movie S3. Case 1 - Induced twisting curvature due to application of axial strain (n=3)

Movie S4. Case 1 - Induced twisting curvature due to application of axial strain (n=4)

Movie S5. Case 1 - Induced twisting curvature due to application of axial strain (n=5)

Movie S6. Case 3 - Induced axial strain due to application of twisting curvature (n=1)

Movie S7. Case 3 - Induced axial strain due to application of twisting curvature (n=2)

Movie S8. Case 3 - Induced axial strain due to application of twisting curvature (n=3)

Movie S9. Case 3 - Induced axial strain due to application of twisting curvature (n=4)

Movie S10. Case 3 - Induced axial strain due to application of twisting curvature (n=5)

Movie S11. Induced bending curvature due to application of shear strain (n=1)

References

[1] Andrew Alderson, Kim L Alderson, Daphne Attard, Kenneth E Evans, Ruben Gatt, Joseph N Grima, William Miller, N Ravirala, CW Smith, and K Zied. Elastic constants of 3-, 4-and 6-connected chiral and anti-chiral honeycombs subject to uniaxial in-plane loading. *Composites Science and Technology*, 70(7):1042–1048, 2010.

[2] Oriol Arteaga, Jordi Sancho-Parramon, Shane Nichols, Ben M Maoz, Adolf Canillas, Salvador Bosch, Gil Markovich, and Bart Kahr. Relation between 2d/3d chirality and the appearance of chiroptical effects in real nanostructures. *Optics express*, 24(3): 2242–2252, 2016.

[3] A Benoussan, Jacques-Louis Lions, and G Papanicolau. *Asymptotic analysis for periodic structures*. North-Holland, 1978.

[4] AB Comsol. Comsol multiphysics® v. 6.1. *Stockholm, Sweden*, 2022.

[5] Walter Friedrich, Paul Knipping, and Max Laue. Interferenzerscheinungen bei roentgenstrahlen. *Annalen der Physik*, 346(10): 971–988, 1913.

[6] Joseph N Grima, Ruben Gatt, and Pierre-Sandre Farrugia. On the properties of auxetic meta-tetrachiral structures. *physica status solidi (b)*, 245(3):511–520, 2008.

[7] Andrew Gross, Panos Pantidis, Katia Bertoldi, and Simos Gerasimidis. Correlation between topology and elastic properties of imperfect truss-lattice materials. *Journal of the Mechanics and Physics of Solids*, 124:577–598, 2019.

[8] JoséMiranda Guedes and Noboru Kikuchi. Preprocessing and postprocessing for materials based on the homogenization method with adaptive finite element methods. *Computer methods in applied mechanics and engineering*, 83(2):143–198, 1990.

[9] H Haddadi and S Belhabib. Use of rigid-body motion for the investigation and estimation of the measurement errors related to digital image correlation technique. *Optics and Lasers in Engineering*, 46(2):185–196, 2008.

[10] Scott J Hollister and Noboru Kikuchi. A comparison of homogenization and standard mechanics analyses for periodic porous composites. *Computational Mechanics*, 10(2):73–95, 1992.

[11] A Gerhard Holzapfel. *Nonlinear solid mechanics II*. John Wiley & Sons, Inc., 2000.

[12] Frederick A Howes and Stephen Whitaker. The spatial averaging theorem revisited. *Chemical engineering science*, 40(8):1387–1392, 1985.

[13] David Lecompte, ASHJD Smits, Sven Bossuyt, Hugo Sol, John Vantomme, Danny Van Hemelrijck, and AM Habraken. Quality assessment of speckle patterns for digital image correlation. *Optics and lasers in Engineering*, 44(11):1132–1145, 2006.

[14] J-L Lions. Remarks on some asymptotic problems in composite materials and in perforated materials. *Variational Methods in Mechanics or Solids*, pages 3–19, 1979.

[15] J-L Lions. On some homogenization problems. *ZAMM-Journal of Applied Mathematics and Mechanics/Zeitschrift für Angewandte Mathematik und Mechanik*, 62(5):T251–T262, 1982.

[16] Lu Liu, Paul Kamm, Francisco García-Moreno, John Banhart, and Damiano Pasini. Elastic and failure response of imperfect three-dimensional metallic lattices: the role of geometric defects induced by selective laser melting. *Journal of the Mechanics and Physics of Solids*, 107:160–184, 2017.

[17] MJ McGinnis, S Pessiki, and H Turker. Application of three-dimensional digital image correlation to the core-drilling method. *Experimental Mechanics*, 45(4):359–367, 2005.

[18] RD Mindlin and HF Tiersten. Effects of couple-stresses in linear elasticity. Technical report, COLUMBIA UNIV NEW YORK, 1962.

[19] Luke Mizzi, EM Mahdi, Kirill Titov, Ruben Gatt, Daphne Attard, Kenneth E Evans, Joseph N Grima, and Jin-Chong Tan. Mechanical metamaterials with star-shaped pores exhibiting negative and zero poisson's ratio. *Materials & Design*, 146:28–37, 2018.

[20] Franz Ernst Neumann. *Vorlesungen über die Theorie der Elasticität der festen Körper und des Lichtäthers: gehalten an der Universität Königsberg*, volume 4. BG Teubner, 1885.

[21] Robert E Newnham. *Properties of materials: anisotropy, symmetry, structure*. Oxford University Press on Demand, 2005.

[22] D Prall and RS Lakes. Properties of a chiral honeycomb with a poisson's ratio of—1. *International Journal of Mechanical Sciences*, 39(3):305–314, 1997.

[23] Falk Runkel, G Ramstein, Giulio Molinari, Andres F Arrieta, and Paolo Ermanni. Mechanics of curved-ligament hexachiral metastructures under planar deformations. *Journal of the Mechanics and Physics of Solids*, 125:145–163, 2019.

[24] Enrique Sánchez-Palencia. Non-homogeneous media and vibration theory. *Lecture notes in physics*, 127, 1980.

[25] Jiahao Shi, Hossein Mofatteh, Armin Mirabolghasemi, Gilles Desharnais, and Abdolhamid Akbarzadeh. Programmable multistable perforated shellular. *Advanced Materials*, 33(42):2102423, 2021.

[26] Thomas Tancogne-Dejean, Nikolaos Karathanasopoulos, and Dirk Mohr. Stiffness and strength of hexachiral honeycomb-like metamaterials. *Journal of Applied Mechanics*, 86(11), 2019.

[27] Woldemar Voigt et al. *Lehrbuch der kristallphysik*, volume 962. Teubner Leipzig, 1928.

[28] Sun Yaofeng and John HL Pang. Study of optimal subset size in digital image correlation of speckle pattern images. *Optics and lasers in engineering*, 45(9):967–974, 2007.
